# Synthesis and In Vitro Cytotoxicity and Antibacterial Activity of Novel 1,2,3-Triazol-5-yl-Phosphonates

**DOI:** 10.3390/molecules25112643

**Published:** 2020-06-06

**Authors:** Anna Tripolszky, Emese Tóth, Pál Tamás Szabó, László Hackler, Beáta Kari, László G. Puskás, Erika Bálint

**Affiliations:** 1 Department of Organic Chemistry and Technology, Budapest University of Technology and Economics, H-1521 Budapest, Hungary; tripolszky.anna@mail.bme.hu (A.T.); emesetoth92@gmail.com (E.T.); 2 MS Metabolomics Laboratory, Instrumentation Center, Research Centre for Natural Sciences, Hungarian Academy of Sciences, Magyar tudósok krt. 2., 1117 Budapest, Hungary; szabo.pal@ttk.mta.hu; 3 Avidin Ltd., Alsó kikötő sor 11/D, H-6726 Szeged, Hungary; l.hackler@avidinbiotech.com (L.H.J.); b.kari@avidinbiotech.com (B.K.)

**Keywords:** 1,2,3-triazoles, triazolyl phosphonates, copper(I) catalyst, domino reaction, multicomponent reaction, anticancer activity

## Abstract

Novel 1,2,3-triazol-5-yl-phosphonates were prepared by the copper(I)-catalyzed domino reaction of phenylacetylene, organic azides and dialkyl phosphites. The process was optimized on the synthesis of the dibutyl (1-benzyl-4-phenyl-1*H*-1,2,3-triazol-5-yl)phosphonate in respect of the catalyst, the base and the solvent, as well as of the reaction parameters (molar ratio of the starting materials, atmosphere, temperature and reaction time). The method elaborated could be applied to a range of organic azides and dialkyl phosphites, which confirmed the large scope and the functional group tolerance. The in vitro cytotoxicity on different cell lines and the antibacterial activity of the synthesized 1,2,3-triazol-5-yl-phosphonates was explored. According to the IC_50_ values determined, only modest antibacterial effect was detected, while some derivatives showed moderate activity against human promyelocytic leukemia HL-60 cells.

## 1. Introduction

Heterocyclic scaffolds bearing phosphonate moieties have been attracting continuous interest due to their importance in synthetic- and medicinal chemistry, as well as in the agriculture and plastic industry [1,2,3,4]. One of the most convenient and efficient tools for the preparation of heterocyclic phosphonates are multicomponent reactions [5], which usually utilize simple starting materials, and meet several criteria of a theoretical “ideal synthesis”, such as high atom economy, fast and simple accomplishment, ability to save time and energy and being as environmentally-friendly as possible [6,7]. They may also be feasible for the creation of large libraries of structurally related compounds.

In the last decades, 1,2,3-triazoles have received significant attention due to the wide range of their possible applications in materials science, biochemistry or even in drug design [8,9,10]. Compounds containing the 1,2,3-triazole scaffold may have versatile biological activities, such as antibacterial, antiviral, antifungal, anticancer or anti-inflammatory effects [11,12,13]. Some novel triazole-based heterocycles showed activity against bacterial strains (*Bacillus subtilis* and *Escherichia coli*) [14,15,16], and in vitro cytotoxicity against tumor cell lines HL-60 (myeloid leukemia), MCF-7 (breast cancer), A549 (lung cancer) and HCT-116 (colon cancer) [17,18,19]. Furthermore, the unique structural properties of 1,2,3-triazoles enable the introduction of various functional groups obtaining new biologically active molecules.

1,2,3-Triazolyl phosphonates combine the advantages of the triazole- and the phosphonate moieties [20]. Several derivatives showed anti-HIV effect [21,22], were investigated in protein binding assays [23] or could be appropriate for bioconjugation [24,25].

The Huisgen 1,3-dipolar cycloaddition [26,27] between azides and alkynyl phosphonates is one of the most common synthetic routes towards 1,2,3-triazol-5-yl-phosphonates (Table 1). Hall and Trippett studied the cycloaddition of ethyl (diethoxyphosphinyl)propynoate and methyl azidoacetate in the absence of any catalyst in boiling toluene (Table 1, Entry 1) [28]. The reaction was not selective; two regioisomers, 1,2,3-triazol-5-yl-phosphonate (**1**) and 1,2,3-triazol-4-yl-phosphonate (**2**), were formed in a ratio of ca. 2:1, respectively. Trifluoromethylated triazolyl phosphonates were also synthesized by the catalyst-free addition of *tert*-butyl azidoacetate to diisopropyl 3,3,3-trifluoroprop-1-ynylphosphonate at room temperature (Table 1, Entry 2) [29]. The click reaction of benzyl azide and diethyl (ethynyl)phosphonates was also carried out in the presence of copper(II) sulfate pentahydrate and sodium ascorbate in DMF (Table 1, Entry 3) [30]. The corresponding 1,2,3-triazol-5-yl-phosphonates (**1**) were obtained selectively at 170 °C after 12 h. Odinets and co-workers performed the cycloaddition of ω-phosphorylalkyl azide to tetramethoxy acetylenediphosphonate in water (Table 1, Entry 4) [31]. A microwave-assisted reaction was also reported, in which diethyl (azidomethyl)phosphonate was reacted with diethyl prop-1-ynylphosphonate under catalyst- and solvent-free conditions (Table 1, Entry 5) [32].

Although the 1,3-dipolar cycloaddition is an important synthetic possibility of triazolyl phosphonates, the preparation of the starting alkynyl phosphonate derivatives is a real challenge. By the development of multicomponent approaches for this compound family, utilization of simple building blocks as starting materials may become available. 

**Scheme 1:**
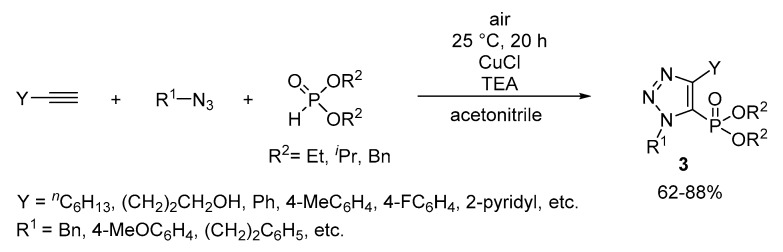
Synthesis of 1,2,3-triazol-5-yl-phosphonates (**3**).

Several examples were reported on the preparation of 1,2,3-triazol-4-yl-phosphonates by domino or three-component reactions [33,34], however, to the best of our knowledge, only one example mentions the multicomponent synthesis of 1,2,3-triazol-5-yl-phosphonates [20]. Li and co-workers carried out the domino reaction of terminal alkynes, azides, and diethyl-, diisopropyl- or dibenzyl phosphite in the presence of copper(I) chloride as the catalyst (Scheme 1).

**Scheme 2:**
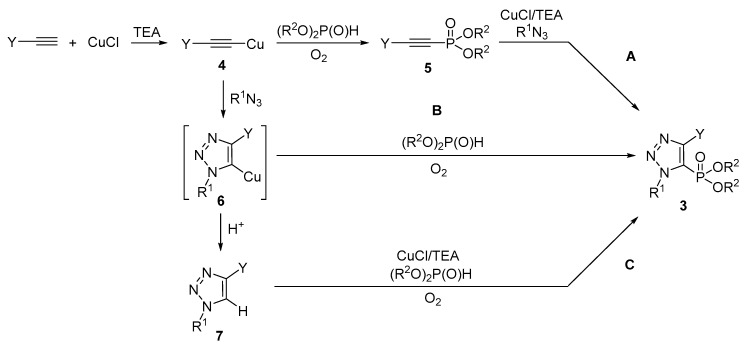
Proposed reaction mechanism for the Cu(I)-catalyzed domino reaction [20].

The mechanism of the three-component reaction has also been investigated [20]. A detailed proposal in Scheme 2 indicates the three possible pathways (**A**–**C**) of the reaction. According to pathway **A**, the acetylene reacts with the dialkyl phosphite by an oxidative coupling to give alkynylphosphonate **5**, which may react with the azide by click reaction to generate the 1,2,3-triazol-5-yl-phosphonate (**3**). Based on pathway **B**, the aerobic oxidative coupling of the dialkyl phosphite with the in situ-formed organocopper intermediate (**6**) may lead to triazol-5-yl-phosphonate (**3**). While in pathway **C**, the click reaction of acetylene with azide provides triazole **7**, which undergoes an oxidative coupling with the dialkyl phosphite resulting in product **3**. It was concluded that the reaction took place through pathway **B**.

In this paper, we report on the synthesis of novel 1,2,3-triazol-5-yl-phosphonates by the copper(I)-catalyzed three-component domino reaction of phenylacetylene, various organic azides and dialkyl phosphites. The biological activity of the compounds synthesized was also studied in antibacterial activity and in vitro cytotoxicity assays. 

## 2. Results and Discussion

In our prior work, the copper(I)-catalyzed domino reaction of phenylacetylene, benzyl azide and diethyl phosphite was investigated [35]. The results reported herein include a comprehensive optimization of the domino reaction using dibutyl phosphite (DBP) as a phosphorus reagent, in respect of the molar ratio of the starting materials, the atmosphere, the additives (catalyst, base and solvent), and of the temperature and the reaction time as well (Table 2).

At first, the multicomponent reaction was performed in the presence of 10 mol% of CuCl as a catalyst and two equivalents of TEA (triethylamine) as a base at room temperature in acetonitrile under different atmospheres. Reacting the three components at a molar ratio of 1:1.1:1.1 under nitrogen atmosphere, the 1-benzyl-4-phenyl-1*H*-1,2,3-triazole (**9**) {[M + H]^+^_found_ = 236.1165, C_15_H_14_N_3_ requires 236.1188} was obtained as the main product instead of the desired triazol-5-yl-phosphonate (**8**) (Table 2, Entry 1). Repeating the reaction using continuous air bubbling, the triazol-5-yl-phosphonate (**8**) was formed in 55%, along with 42% of the “click product” (**9**) and 3% of dibutyl (phenylethynyl)phosphonate (**10**) {^31^P NMR (CDCl_3_) δ −5.8; [M + H]^+^_found_ = 295.1440, C_16_H_24_O_3_P requires 295.1463} (Table 2, Entry 2). Applying a continuous oxygen bubbling, the proportion of alkynylphosphonate (**10**) significantly increased and the 1,2,3-triazol-5-yl-phosphonate (**8**) was obtained only in 24% (Table 2, Entry 3). After that, the effect of various bases was investigated, thus the domino reaction was also carried out in the presence of DPA (dipropylamine), DIPA (diisopropylamine) or DIPEA (*N*,*N*-diisopropylethylamine) (Table 2, Entries 4–6). In terms of the formation of the desired 1,2,3-triazol-5-yl-phosphonate (**8**), TEA was proved to be the most efficient. The effect of the solvent was also studied, from among the solvents tried out (MeCN, DMF, DCM, THF and EtOH), MeCN turned out as the most suitable (Table 2, Entries 2 and 7–10). In the next step, the counterion of the copper catalyst was varied. The reaction was performed with CuCl, CuBr, CuI or CuSO_4_·5H_2_O/NaAsc, and the best result was achieved using CuCl (Table 2, Entries 2,11–13). Increasing the amount of dibutyl phosphite to two equivalents, the proportion of the desired product (**8**) could be further improved (59%) (Table 2, Entry 14). Applying a larger excess of dibutyl phosphite or more CuCl did not favor the formation of the triazol-5-yl-phosphonate (**8**) (Table 2, Entries 15 and 16). Finally, the effect of the temperature was investigated. Neither lowering (0 °C) nor raising (50 °C) the temperature caused improvement in the ratio of 1,2,3-triazol-5-yl-phosphonate (**8**) (Table 2, Entries 17 and 18). The best result was obtained using two equivalents of dibutyl phosphite in the presence of two equivalents of TEA and 10 mol% of CuCl at room temperature in acetonitrile and continuous air bubbling (Table 2, Entry 14).

**Figure 1:**
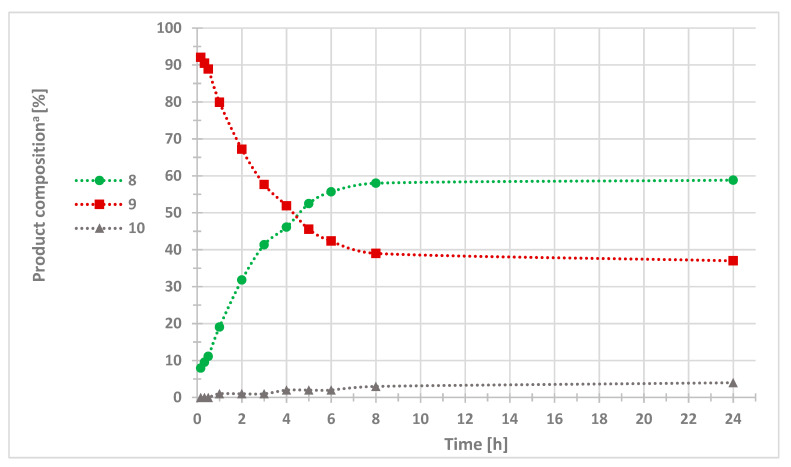
Time dependence of the product composition in the model reaction of phenylacetylene, benzyl azide and dibutyl phosphite. Conditions: phenylacetylene (1 equiv.), benzyl azide (1.1 equiv.), dibutyl phosphite (2 equiv.), CuCl (0.1 equiv.) and TEA (2 equiv.) were stirred in MeCN with continuous air bubbling at 25 °C for 24 h. ^a^ Based on HPLC (222 nm).

Applying the optimal parameters, the reaction time was also investigated. It was found that, during the first eight hours, the proportion of the desired dibutyl (1-benzyl-4-phenyl-1*H*-1,2,3-triazol-5-yl)phosphonate (**8**) increased, and the proportion of the 1-benzyl-4-phenyl-1*H*-1,2,3-triazole (**9**) continuously decreased (Figure 1). It should be noted that the dibutyl (phenylethynyl)phosphonate (**10**) did not form in a significant amount during the observed 24 h. Based on Figure 1, a reaction time of eight hours was considered as optimal for the domino reactions.

**Scheme 3:**
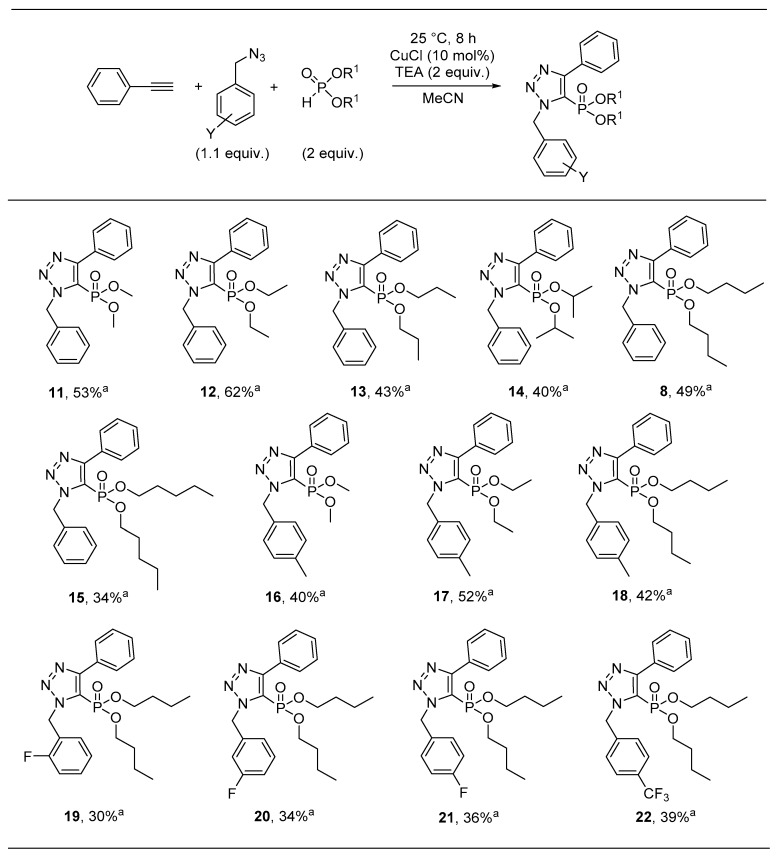
Domino reaction of phenylacetylene, substituted benzyl azides and dialkyl phosphites. ^a^ Isolated yield.

In the next series of experiments, the three-component reaction of phenylacetylene, various benzyl azides and dialkyl phosphites was investigated under the optimized conditions (Scheme 3). First, the domino reaction of phenylacetylene and benzyl azide was carried out with a wide range of dialkyl phosphites. Using dimethyl phosphite, the dimethyl (1-benzyl-4-phenyl-1,2,3-triazol-5-yl)phosphonate (**11**) was isolated in a yield of 53%. Performing the reactions with diethyl- or dipropyl phosphite, the product **12** and **13** was prepared in a yield of 62% and 43%, respectively. Changing for a dialkyl phosphite containing branched alkyl chains, such as diisopropyl phosphite, the desired triazol-5-yl-phosphonate (**14**) was obtained in a yield of 40% after column chromatography. The dibutyl (1-benzyl-4-phenyl-1*H*-1,2,3-triazol-5-yl)phosphonate (**8**) was isolated from the experiments of Table 2, Entry 14 in a yield of 49%. Applying a dialkyl phosphite incorporating long alkyl chains, such as dipentyl phosphite, the corresponding dipentyl (1-benzyl-4-phenyl-1,2,3-triazol-5-yl)phosphonate (**15**) was prepared in a yield of 34%. After that, the domino reaction was performed with substituted benzyl azides (4-methylbenzyl azide 2-, 3- or 4-fluorobenzyl azide or 4-(trifluoromethyl)benzyl azide). Carrying out the reaction of phenylacetylene and 4-methylbenzyl azide with dimethyl-, diethyl- or dibutyl phosphite, the desired triazol-5-yl-phosphonates (**16**–**18**) could be isolated in yields of 40%, 52% and 42%, respectively. Finally the domino reaction of phenylacetylene, and dibutyl phosphite was also carried out with 2-, 3- or 4-fluorobenzyl azide or 4-(trifluoromethyl)benzyl azide, and the corresponding dibutyl (1-benzyl-4-phenyl-1*H*-1,2,3-triazol-5-yl)phosphonate derivatives (**19**–**22**) were synthesized in yields of 30–39%.

**Scheme 4:**
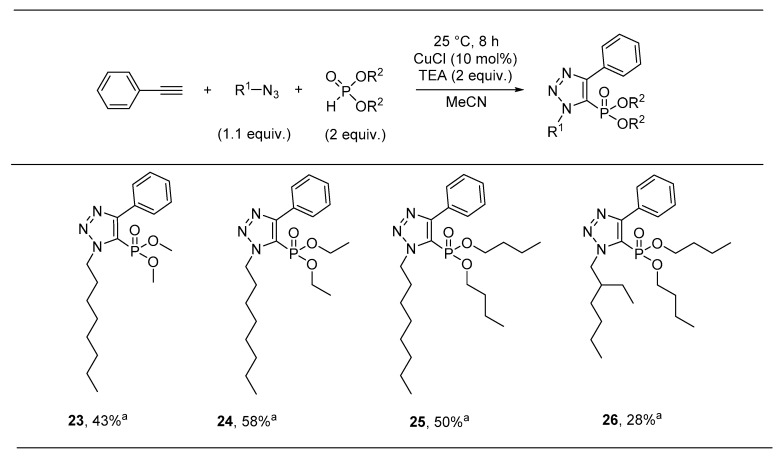
Domino reaction of phenylacetylene, aliphatic azides and dialkyl phosphites. ^a^ Isolated yield.

The CuCl-catalyzed domino reaction was also carried out with aliphatic azides, such as octyl or *iso-*octyl azide using the optimized conditions (Scheme 4). In the reactions of phenylacetylene, octyl azide and dimethyl-, diethyl- or dibutyl phosphite, the corresponding 1-octyl-4-phenyl-1*H*-1,2,3-triazolyl phosphonates (**23**–**25**) were prepared in yields of 43–58%. Finally, *iso*-octyl azide was reacted with phenylacetylene and dibutyl phosphite, and the desired product (**26**) was obtained in a yield of 28%, possibly due to the steric hindrance of the 2-ethylhexyl group.

The multicomponent approach developed is a fast and simple methodology for the synthesis of 1,2,3-triazol-5-yl-phosphonates, which utilizes simple starting materials and applies milder reaction conditions compared to the preparation of these compounds by 1,3-dipolar cycloaddition. In contrast to a previous report dealing with a similar topic [20], besides a comprehensive optimization, we have provided exact product compositions, including the amount of the “click product” and the amount of the (phenylethynyl)phosphonate, respectively. The scope of the reaction was extended for 15 novel derivatives. (Copies of ^31^P, ^1^H, and ^13^C NMR spectra for all compounds synthesized are presented in Supplementary Materials).

Finally, the biological activity of the synthesized 1,2,3-triazol-5-yl-phosphonates (**8**, **11**–**26**) was investigated. On the one hand, the antibacterial activity of the compounds was tested on GFP producing Gram-positive (*Bacillus subtilis*) and Gram-negative (*Escherichia coli*) bacterial cells. The GFP producing bacteria are an effective tool for screening for antibacterial activity since GFP signal measured via fluorimetry is proportional to the number of bacterial cells. Active compounds would kill bacterial cells, which results in the decrease in GFP fluorescence signal, therefore it is suitable for evaluating the antimicrobial effect of different agents. On the other hand, in vitro cytotoxicity assessments were performed on three different cell lines, such as human lung adenocarcinoma (A549), mouse fibroblast (NIH/3T3) as healthy cell line and human promyelocytic leukemia (HL-60). Table 3 contains the determined IC_50_ values in antimicrobial and cytotoxicity assays. According to the results, only modest antibacterial activity was detected against the more sensitive *Bacillus subtilis* strain. The IC_50_ values of triazolyl phosphonates (**8**, **13**, **15**, **16**, **19**–**22** and **26**), which showed some activity against NIH/3T3 and/or HL-60 cells fall in the range of 9.7 and 27.5 µM. Dimethyl [1-(4-methylbenzyl)-4-phenyl-1,2,3-triazol-5-yl]phosphonate (**16**) and dibutyl [1-(4-trifluoromethyl)-4-phenyl-1,2,3-triazol-5-yl]phosphonate (**22**) proved to be the most active compounds, which showed activity in the 10 micromolar range against HL-60 cells.

## 3. Materials and Methods 

### 3.1. General

The reactions under conventional heating were carried out in an oil bath. The microwave-assisted experiments were performed in a 300 W CEM Discover focused microwave reactor (CEM Microwave Technology Ltd., Buckingham, UK) equipped with a pressure controller using 5–10 W irradiation under isothermal conditions.

HPLC-MS measurements were performed with an Agilent 1200 liquid chromatography system coupled with a 6130 quadrupole mass spectrometer equipped with an ESI ion source (Agilent Technologies, Palo Alto, CA, USA). The analysis was performed at 40 °C on a Gemini C18 column (150 mm × 4.6 mm, 3 µm; Phenomenex, Torrance, CA, USA) with a mobile phase flow rate of 0.6 mL/min. The composition of eluent A was 0.1% (NH_4_)(HCOO) in water; eluent B was 0.1% (NH_4_)(HCOO) and 8% water in acetonitrile. 0–3 min. 5% B, 3–13 min. gradient, 13–20 min. 100% B. The injection volume was 5 µL. The chromatographic profile was registered at 222 nm. The MSD operating parameters were as follows: positive ionization mode, scan spectra from *m*/*z* 120 to 1200, drying gas temperature 300 °C, nitrogen flow rate 10 L/min, nebulizer pressure 60 psi, capillary voltage 4000 V.

High resolution mass spectrometric measurements were performed using a Sciex 5600+ Q-TOF mass spectrometer in positive electrospray mode.

The ^31^P, ^1^H, ^13^C, NMR spectra were taken in CDCl_3_ solution on a Bruker AV-300 spectrometer operating at 121.5, 300 and 75.5 MHz, respectively. Chemical shifts are downfield relative to 85% H_3_PO_4_ and TMS.

### 3.2. General Procedure for the Synthesis of Organic Azides 

#### 3.2.1. Method A

To a stirred solution of 10.0 mmol alkyl halides (1.19 mL of benzyl bromide, 1.85 g of 4-methylbenzyl bromide, 1.21 mL of 2-fluorobenzyl bromide, 1.23 mL of 3-fluorobenzyl bromide, 1.25 mL of 4-fluorobenzyl bromide or 1.55 mL of 4-(trifluoromethyl)benzyl bromide) in 100 mL of acetone/H_2_O 4:1 (*v*/*v*) was added 15.0 mmol (0.98 g) of sodium azide. The reaction mixture was stirred at room temperature for 24 h. After that, the reaction was extracted with Et_2_O (3 × 50 mL), dried over Na_2_SO_4_, and concentrated under reduced pressure to give benzyl azides as pale yellow oils. 

#### 3.2.2. Method B

To a stirred solution of 10.0 mmol alkyl halides (1.76 mL of octyl bromide or 1.78 mL of iso-octyl bromide) in 20 mL of DMF, 12.0 mmol (0.78 g) of sodium azide was added. The reaction mixture was stirred at 70 °C for 24 h in an oil bath. After, the reaction was extracted with Et_2_O (3 × 50 mL), dried over Na_2_SO_4_, and concentrated under reduced pressure to give alkyl azides as pale yellow oils. 

The following organic azides were prepared using method A or method B (Table 4):

### 3.3. General Procedure for the Synthesis of Dipropyl and Dipentyl Phosphite

A mixture of 5.0 mmol (0.64 mL) of diethyl phosphite and 25 equivalents, and 150.0 mmol of alcohol (10.0 mL *n*-propanol or 14.0 mL *n*-pentanol) was heated at 175 °C in a vial in the MW reactor for 120–135 min. The volatile components were removed in vacuum and the crude product was purified by column chromatography using silica gel as the solid phase and ethyl acetate as the eluent. The following dialkyl phosphites were thus prepared (Table 5):

### 3.4. General Procedure for the Synthesis of 1,2,3-Triazol-5-yl-Phosphonates

In combination, 1.0 mmol phenylacetylene (0.12 mL), 1.1 mmol organic azide (0.14 g of benzyl azide, 0.17 g of 4-methylbenzyl azide, 0.17 g of 2-fluorobenzyl azide, 0.17 g of 3-fluorobenzyl azide, 0.17 g of 4-fluorobenzyl azide, 0.22 g of 4-(trifluoromethyl)benzyl azide, 0.18 g of octyl azide or 0.18 g of *iso*-octyl azide) and 2.0 mmol dialkyl phosphite (0.18 mL dimethyl phosphite, 0.26 mL diethyl phosphite, 0.33 g dipropyl phosphite, 0.33 mL diisopropyl phosphite, 0.40 mL dibutyl phosphite, 0.44 g dipentyl phosphite) were dissolved in acetonitrile (20 mL). To this was added 2 mmol (0.28 mL) triethylamine and 0.10 mmol (0.02 g) CuCl. The mixture was stirred at room temperature with continuous air bubbling for 8 h. The resulting solution was extracted with ethyl acetate (3 × 30 mL) and the combined organic layers were dried over Na_2_SO_4_. After evaporating the solvent, the crude product was purified by column chromatography using silica gel and ethyl acetate/hexane 1:1 as the eluent and, if it was necessary, by reversed-phase column chromatography using C_18_-reversed-phase silica gel and the composition of eluent A was 0.1% (NH_4_)(HCOO) in water; eluent B was 0.1% (NH_4_)(HCOO) and 8% water in acetonitrile. The following products were thus prepared:

*Dimethyl (1-benzyl-4-phenyl-1,2,3-triazol-5-yl)phosphonate* (**11**): yield: 53% (0.18 g), colorless oil; ^31^P NMR (CDCl_3_) δ 7.5; ^1^H NMR (CDCl_3_) δ 3.40 (d, 6H, ^3^*J*_HP_ = 11.8, OCH_3_), 6.01 (s, 2H, CH_2_Ph), 7.26–7.36 (m, 3H, C_3’_H, C_4’_H), 7.36–7.45 (m, 5H, C_3_H, C_4_H, C_2’_H), 7.74 (dd, 2H, ^3^*J*_HH_ = 7.4, *J*_HH_ = 2.2, C_2_H); ^13^C NMR (CDCl_3_) δ 53.0 (d, ^2^*J*_CP_ = 5.1, OCH_3_), 54.3 (CH_2_Ph), 120.5 (d, ^1^*J*_CP_ = 219.4, P(O)C=), 128.2 (C_2_), 128.3 (C_2’_), 128.5 (C_4’_), 128.8 (C_3’_), 129.1 (C_3_), 129.2 (C_1_), 130.0 (C_4_), 135.9 (C_1’_), 153.9 (d, ^2^*J*_CP_ = 19.5, PhC=); [M + H]^+^_found_ = 344.1161, C_17_H_19_N_3_O_3_P requires 344.1164.

*Diethyl (1-benzyl-4-phenyl-1,2,3-triazol-5-yl)phosphonate* (**12**): yield: 62% (0.23 g), colorless oil; ^31^P NMR (CDCl_3_) δ 4.4; δ [20] (CDCl_3_) δ 4.53; ^1^H NMR (CDCl_3_) δ 0.98 (t, 6H, ^3^*J*_HH_ = 7.1, OCH_2_C*H*_3_), 3.58–3.72 (m, 2H, OC*H*_2_CH_3_), 3.82–3.95 (m, 2H, OC*H*_2_CH_3_), 6.03 (s, 2H, CH_2_Ph), 7.26–7.36 (m, 3H, C_3’_H, C_4’_H), 7.37–7.44 (m, 5H, C_3_H, C_4_H, C_2’_H), 7.76 (dd, 2H, ^3^*J*_HH_ = 7.4, *J*_HH_ = 2.2, C_2_H); δ [20] (CDCl_3_) 0.96 (t, 6H, *J* = 7.2 Hz), 3.57–3.67 (m, 2 H), 3.81–3.91 (m, 2 H), 6.01 (s, 2 H), 7.30–7.40 (m, 8 H), 7.73–7.75 (m, 2 H); ^13^C NMR (CDCl_3_) δ 15.9 (d, ^3^*J*_CP_ = 7.0, OCH_2_*C*H_3_), 54.2 (CH_2_Ph), 63.1 (d, ^2^*J*_CP_ = 5.2, O*C*H_2_CH_3_), 121.6 (d, ^1^*J*_CP_ = 217.5, P(O)C=), 128.2 (C_2_), 128.3 (C_2’_), 128.4(C_4’_), 128.7 (C_3’_), 129.1 (C_1_), 129.3 (C_3_), 130.3 (C_4_), 135.9 (C_1’_), 153.6 (d, ^2^*J*_CP_ = 19.0, PhC=); δ [20] (CDCl_3_) 14.8, 14.9, 53.2, 62.0, 62.1, 119.4, 121.6, 127.2, 127.3, 127.4, 127.7, 128.1, 128.3, 129.2, 134.9, 152.6; [M + H]^+^_found_ = 372.1479, C_19_H_23_N_3_O_3_P requires 372.1477.

*Dipropyl (1-benzyl-4-phenyl-1,2,3-triazol-5-yl)phosphonate* (**13**): yield: 43% (0.17 g), colorless oil; ^31^P NMR (CDCl_3_) δ 4.6; ^1^H NMR (CDCl_3_) δ 0.68 (t, 6H, ^3^*J*_HH_ = 7.4, OCH_2_CH_2_C*H*_3_), 1.27–1.42 (m, 4H, OCH_2_C*H*_2_CH_3_), 3.42–3.60 (m, 2H, OC*H*_2_CH_2_CH_3_), 3.70–3.84 (m, 2H, OC*H*_2_CH_2_CH_3_), 6.04 (s, 2H, CH_2_Ph), 7.25–7.35 (m, 3H, C_3’_H, C_4’_H), 7.35–7.45 (m, 5H, C_3_H, C_4_H, C_2’_H), 7.77 (dd, 2H, ^3^*J*_HH_ = 7.2, *J*_HH_ = 2.5, C_2_H); ^13^C NMR (CDCl_3_) δ 9.9 (OCH_2_CH_2_*C*H_3_)_,_ 23.4 (d, ^3^*J*_CP_ = 7.1, OCH_2_*C*H_2_CH_3_), 54.2 (CH_2_Ph), 68.4 (d, ^2^*J*_CP_ = 5.6, O*C*H_2_CH_2_CH_3_), 121.6 (d, ^1^*J*_CP_ = 217.9, P(O)C=), 128.2 (C_2_), 128.4 (C_2’_), 128.4(C_4’_), 128.7 (C_3’_), 129.1 (C_1_), 129.2 (C_3_), 130.3 (C_4_), 136.0 (C_1’_), 153.6 (d, ^2^*J*_CP_ = 19.0, PhC=); [M + H]^+^_found_ = 400.1778, C_21_H_27_N_3_O_3_P requires 400.1790.

*Diisopropyl (1-benzyl-4-phenyl-1,2,3-triazol-5-yl)phosphonate* (**14**): yield: 40% (0.16 g), colorless oil; ^31^P NMR (CDCl_3_) δ 2.0; δ [20] (CDCl_3_) 2.19; ^1^H NMR (CDCl_3_) δ 0.83 (d, 6H, ^3^*J*_HH_ = 6.2, OCH(C*H*_3_)_2_), 1.19 (d, 6H, ^3^*J*_HH_ = 6.2, OCH(C*H*_3_)_2_), 4.23–4.44 (m, 2H, OC*H*(CH_3_)_2_), 6.06 (s, 2H, CH_2_Ph), 7.24–7.35 (m, 3H, C_3’_H, C_4’_H), 7.36–7.45 (m, 5H, C_3_H, C_4_H, C_2’_H), 7.81 (dd, 2H, ^3^*J*_HH_ = 7.6, *J*_HH_ = 2.2, C_2_H); δ [20] (CDCl_3_) 0.80 (d, 6H, *J* = 6.4 Hz), 1.17 (d, 6H, *J* = 6.0 Hz), 4.27–4.35 (m, 2 H), 6.05 (s, 2 H), 7.27–7.39 (m, 8 H), 7.79 (d, 2 H, *J* = 5.6 Hz); ^13^C NMR (CDCl_3_) δ 23.2 (d, ^3^*J*_CP_ = 5.6, OCH(*C*H_3_)_2_), 23.9 (d, ^3^*J*_CP_ = 3.9, OCH(*C*H_3_)_2_), 54.2 (CH_2_Ph), 72.6 (d, ^2^*J*_CP_ = 5.5, O*C*H(CH_3_)_2_), 122.6 (d, ^1^*J*_CP_ = 218.6, P(O)C=), 128.1 (C_2_), 128.29 (C_4’_), 128.32 (C_2’_), 128.7 (C_3’_), 128.9 (C_1_), 129.3 (C_3_), 130.6 (C_4_), 136.1 (C_1’_), 153.2 (d, ^2^*J*_CP_ = 18.9, PhC=); δ [20] (CDCl_3_) 22.1, 22.2, 22.8, 22.9, 53.1, 71.6, 120.5, 122.7, 127.1, 127.3, 127.6, 127.9, 128.3, 129.5, 135.1, 152.2; [M + H]^+^_found_ = 400.1777, C_21_H_27_N_3_O_3_P requires 400.1790.

*Dibutyl (1-benzyl-4-phenyl-1,2,3-triazol-5-yl)phosphonate* (**8**): yield: 49% (0.20 g), colorless oil; ^31^P NMR (CDCl_3_) δ 2.2; ^1^H NMR (CDCl_3_) δ 0.76 (t, 6H, ^3^*J*_HH_ = 7.3, OCH_2_CH_2_CH_2_C*H*_3_), 0.99–1.19 (m, 4H, OCH_2_CH_2_C*H*_2_CH_3_), 1.22–1.38 (m, 4H, OCH_2_C*H*_2_CH_2_CH_3_), 3.46–3.62 (m, 2H, OC*H*_2_CH_2_CH_2_CH_3_), 3.72–3.91 (m, 2H, OC*H*_2_CH_2_CH_2_CH_3_), 6.03 (s, 2H, CH_2_Ph), 7.25–7.34 (m, 3H, C_3’_H, C_4’_H), 7.36–7.43 (m, 5H, C_3_H, C_4_H, C_2’_H), 7.76 (dd, 2H, ^3^*J*_HH_ = 6.6, *J*_HH_ = 2.9, C_2_H); ^13^C NMR (CDCl_3_) δ 13.5 (OCH_2_CH_2_CH_2_*C*H_3_), 18.5 (OCH_2_CH_2_*C*H_2_CH_3_), 32.0 (d, ^3^*J*_CP_ = 7.0, OCH_2_*C*H_2_CH_2_CH_3_), 54.2 (CH_2_Ph), 66.7 (d, ^2^*J*_CP_ = 5.5, O*C*H_2_CH_2_CH_2_CH_3_), 121.6 (d, ^1^*J*_CP_ = 217.8, P(O)C=), 128.2 (C_2_), 128.3 (C_2’_), 128.4 (C_4’_), 128.7 (C_3’_), 129.1 (C_1_), 129.3 (C_3_), 130.4 (C_4_), 136.0 (C_1’_), 153.6 (d, ^2^*J*_CP_ = 19.1, PhC=); [M + H]^+^_found_ = 428.2086, C_23_H_31_N_3_O_3_P requires 428.2103.

*Dipentyl (1-benzyl-4-phenyl-1,2,3-triazol-5-yl)phosphonate* (**15**): yield: 34% (0.15 g), colorless oil; ^31^P NMR (CDCl_3_) δ 2.4; ^1^H NMR (CDCl_3_) δ 0.80 (t, 6H, ^3^*J*_HH_ = 7.1, OCH_2_CH_2_CH_2_CH_2_C*H*_3_), 1.00–1.13 (m, 4H, OCH_2_CH_2_CH_2_C*H*_2_CH_3_), 1.13–1.25 (m, 4H, OCH_2_CH_2_C*H*_2_CH_2_CH_3_), 1.25–1.40 (m, 4H, OCH_2_C*H*_2_CH_2_CH_2_CH_3_), 3.47–3.62 (m, 2H, OC*H*_2_CH_2_CH_2_CH_3_), 3.74–3.89 (m, 2H, OC*H*_2_CH_2_CH_2_CH_3_), 6.03 (s, 2H, CH_2_Ph), 7.27–7.37 (m, 3H, C_3’_H, C_4’_H), 7.37–7.47 (m, 5H, C_3_H, C_4_H, C_2’_H), 7.76 (dd, 2H, ^3^*J*_HH_ = 6.6, *J*_HH_ = 3.0, C_2_H); ^13^C NMR (CDCl_3_) δ 14.0 (OCH_2_CH_2_CH_2_CH_2_*C*H_3_), 22.2 (OCH_2_CH_2_CH_2_*C*H_2_CH_3_), 27.4 (OCH_2_CH_2_*C*H_2_CH_2_CH_3_), 29.7 (d, ^3^*J*_CP_ = 7.1, OCH_2_*C*H_2_CH_2_CH_2_CH_3_), 54.2 (CH_2_Ph), 67.0 (d, ^2^*J*_CP_ = 5.5, O*C*H_2_CH_2_CH_2_CH_2_CH_3_), 121.6 (d, ^1^*J*_CP_ = 217.8, P(O)C=), 128.2 (C_2_), 128.3 (C_2’_), 128.4 (C_4’_), 128.7 (C_3’_), 129.1 (C_1_), 129.3 (C_3_), 130.3 (C_4_), 136.0 (C_1’_), 153.6 (d, ^2^*J*_CP_ = 19.1, PhC=); [M + H]^+^_found_ = 456.2398, C_25_H_35_N_3_O_3_P requires 456.2416.

*Dimethyl [1-(4-methylbenzyl)-4-phenyl-1,2,3-triazol-5-yl]phosphonate* (**16**): yield: 40% (0.14 g), colorless oil; ^31^P NMR (CDCl_3_) δ 7.5; ^1^H NMR (CDCl_3_) δ 2.32 (s, 3H, PhCH_3_), 3.43 (d, 6H, ^3^*J*_HP_ = 11.7, OCH_3_), 5.96 (s, 2H, CH_2_Ph), 7.15 (d, 2H, ^3^*J*_HH_ = 7.8, C_3’_H), 7.30 (d, 2H, ^3^*J*_HH_ = 7.9, C_2’_H), 7.40–7.44 (m, 3H, C_3_H, C_4_H), 7.73 (dd, 2H, ^3^*J*_HH_ = 7.3, *J*_HH_ = 2.4, C_2_H); ^13^C NMR (CDCl_3_) δ 21.3 (CH_3_Ph), 53.0 (d, ^2^*J*_CP_ = 5.3, OCH_3_), 54.1 (CH_2_Ph), 120.4 (d, ^1^*J*_CP_ = 219.5, P(O)C=), 128.3 (C_2_, C_2’_), 129.15 (C_3’_), 129.17 (C_1_), 129.4 (C_3_), 130.1 (C_4_), 132.8 (C_1’_), 138.3 (C_4’_), 153.8 (d, ^2^*J*_CP_ = 19.4, PhC=); [M + H]^+^_found_ = 358.1315, C_18_H_21_N_3_O_3_P requires 358.1315.

*Diethyl [1-(4-methylbenzyl)-4-phenyl-1,2,3-triazol-5-yl]phosphonate* (**17**): yield: 52% (0.20 g), colorless oil; ^31^P NMR (CDCl_3_) δ 4.3; ^1^H NMR (CDCl_3_) δ 0.99 (t, 6H, ^3^*J*_HH_ = 7.0, OCH_2_C*H*_3_), 2.32 (s, 3H, PhCH_3_), 3.58–3.72 (m, 2H, OC*H*_2_CH_3_), 3.85–3.95 (m, 2H, OC*H*_2_CH_3_), 6.00 (s, 2H, CH_2_Ph), 7.14 (d, 2H, ^3^*J*_HH_ = 7.8, C_3’_H), 7.30 (d, 2H, ^3^*J*_HH_ = 8.0, C_2’_H), 7.38–7.44 (m, 3H, C_3_H, C_4_H), 7.74 (dd, 2H, ^3^*J*_HH_ = 7.7, *J*_HH_ = 2.0, C_2_H); ^13^C NMR (CDCl_3_) δ 15.9 (d, ^3^*J*_CP_ =7.0, OCH_2_*C*H_3_), 21.2 (CH_3_Ph), 54.0 (CH_2_Ph), 63.1 (d, ^2^*J*_CP_ =5.1, O*C*H_2_CH_3_), 121.5 (d, ^1^*J*_CP_ = 217.7, P(O)C=), 128.2 (C_2_), 128.3 (C_2’_), 129.0 (C_1_), 129.3 (C_3’_), 129.4 (C_3_), 130.4 (C_4_), 132.9 (C_1’_), 138.2 (C_4’_), 153.5 (d, ^2^*J*_CP_ = 19.1, PhC=); [M + H]^+^_found_ = 386.1633, C_20_H_25_N_3_O_3_P requires 386.1628.

*Dibutyl [1-(4-methylbenzyl)-4-phenyl-1,2,3-triazol-5-yl]phosphonate* (**18**): yield: 42% (0.19 g), colorless oil; ^31^P NMR (CDCl_3_) δ 3.1; ^1^H NMR (CDCl_3_) δ 0.69 (t, 6H, ^3^*J*_HH_ = 7.3, OCH_2_CH_2_CH_2_C*H*_3_), 0.93–1.10 (m, 4H, OCH_2_CH_2_C*H*_2_CH_3_), 1.11–1.13 (m, 4H, OCH_2_C*H*_2_CH_2_CH_3_), 2.25 (s, 3H, PhCH_3_), 3.41–3.57 (m, 2H, OC*H*_2_CH_2_CH_2_CH_3_), 3.67–3.83 (m, 2H, OC*H*_2_CH_2_CH_2_CH_3_), 5.91 (s, 2H, CH_2_Ph), 7.06 (d, 2H, ^3^*J*_HH_ = 7.8, C_3’_H), 7.22 (d, 2H, ^3^*J*_HH_ = 7.7, C_2’_H), 7.27–7.38 (m, 3H, C_3_H, C_4_H), 7.67 (dd, 2H, ^3^*J*_HH_ = 6.6, *J*_HH_ = 3.0, C_2_H); ^13^C NMR (CDCl_3_) δ 13.6 (OCH_2_CH_2_CH_2_*C*H_3_), 18.5 (OCH_2_CH_2_*C*H_2_CH_3_), 21.3 (CH_3_Ph), 32.0 (d, ^3^*J*_CP_ = 7.1, OCH_2_*C*H_2_CH_2_CH_3_), 54.1 (CH_2_Ph), 66.7 (d, ^2^*J*_CP_ = 5.5, O*C*H_2_CH_2_CH_2_CH_3_), 121.5 (d, ^1^*J*_CP_ = 218.5, P(O)C=), 128.2 (C_2_), 128.4 (C_2’_), 129.0 (C_1_), 129.3 (C_3’_), 129.4 (C_3_), 130.4 (C_4_), 133.1 (C_1’_), 138.2 (C_4’_), 153.5 (d, ^2^*J*_CP_ = 19.3, PhC=); [M + H]^+^_found_ = 442.2242, C_24_H_33_N_3_O_3_P requires 442.2260.

*Dibutyl [1-(2-fluorobenzyl)-4-phenyl-1,2,3-triazol-5-yl]phosphonate* (**19**): yield: 30% (0.13 g), pale yellow oil; ^31^P NMR (CDCl_3_) δ 4.6; ^1^H NMR (CDCl_3_) δ 0.79 (t, 6H, ^3^*J*_HH_ = 7.3, OCH_2_CH_2_CH_2_C*H*_3_), 1.07–1.22 (m, 4H, OCH_2_CH_2_C*H*_2_CH_3_), 1.29–1.42 (m, 4H, OCH_2_C*H*_2_CH_2_CH_3_), 3.59–3.76 (m, 2H, OC*H*_2_CH_2_CH_2_CH_3_), 3.83–3.99 (m, 2H, OC*H*_2_CH_2_CH_2_CH_3_), 6.10 (s, 2H, CH_2_Ph), 7.03–7.15 (m, 3H, C_3’_H, C_5’_H, C_6’_H), 7.24–7.35 (m, 1H, C_4’_H), 7.38–7.47 (m, 3H, C_3_H, C_4_H), 7.79 (dd, 2H, ^3^*J*_HH_ = 7.2, *J*_HH_ = 2.6, C_2_H); ^13^C NMR (CDCl_3_) δ 13.5 (OCH_2_CH_2_CH_2_*C*H_3_), 18.6 (OCH_2_CH_2_*C*H_2_CH_3_), 32.0 (d, ^3^*J*_CP_ = 6.9, OCH_2_*C*H_2_CH_2_CH_3_), 48.2 (d, ^3^*J*_CP_ =4.7, CH_2_Ph), 66.8 (d, ^2^*J*_CP_ = 5.7, O*C*H_2_CH_2_CH_2_CH_3_), 115.7 (d, ^2^*J*_CF_ = 21.1, C_3’_), 122.1 (d, ^1^*J*_CP_ = 218.6, P(O)C=), 123.4 (d, ^2^*J*_CF_ = 14.3, C_1’_), 124.4 (d, ^2^*J*_CF_ = 3.7, C_5’_), 128.2 (C_2_), 129.1 (C_1_), 129.3 (C_3_), 129.4 (d, ^3^*J*_CF_ = 3.2, C_4’_), 130.1 (d, ^3^*J*_CF_ = 8.1, C_6’_), 130.2 (C_4_), 153.5 (d, ^2^*J*_CP_ = 19.2, PhC=), 160.4 (d, ^1^*J*_CF_ = 248.8, C_2’_); [M + H]^+^_found_ = 446.1975, C_23_H_30_N_3_O_3_FP requires 446.2008.

*Dibutyl [1-(3-fluorobenzyl)-4-phenyl-1,2,3-triazol-5-yl]phosphonate* (**20**): yield: 34% (0.15 g), pale yellow oil; ^31^P NMR (CDCl_3_) δ 2.1; ^1^H NMR (CDCl_3_) δ 0.77 (t, 6H, ^3^*J*_HH_ = 7.3, OCH_2_CH_2_CH_2_C*H*_3_), 1.03–1.22 (m, 4H, OCH_2_CH_2_C*H*_2_CH_3_), 1.22–1.42 (m, 4H, OCH_2_C*H*_2_CH_2_CH_3_), 3.53–3.69 (m, 2H, OC*H*_2_CH_2_CH_2_CH_3_), 3.81–3.94 (m, 2H, OC*H*_2_CH_2_CH_2_CH_3_), 6.03 (s, 2H, CH_2_Ph), 6.95–7.05 (m, 1H, C_4’_H), 7.06–7.22 (m, 2H, C_2’_H, C_6’_H), 7.25–7.34 (m, 1H, C_5’_H), 7.36–7.47 (m, 3H, C_3_H, C_4_H), 7.76 (dd, 2H, ^3^*J*_HH_ = 6.4, *J*_HH_ = 3.4, C_2_H); ^13^C NMR (CDCl_3_) δ 13.5 (OCH_2_CH_2_CH_2_*C*H_3_), 18.5 (OCH_2_CH_2_*C*H_2_CH_3_), 32.0 (d, ^3^*J*_CP_ = 7.0, OCH_2_*C*H_2_CH_2_CH_3_), 53.5 (CH_2_Ph), 66.8 (d, ^2^*J*_CP_ = 5.6, O*C*H_2_CH_2_CH_2_CH_3_), 115.3 (d, ^2^*J*_CF_ = 21.8, C_2’_, C_4’_), 121.6 (d, ^1^*J*_CP_ = 218.0, P(O)C=), 124.0 (d, *J*_CF_ = 3.1, C_6’_), 128.2 (C_2_), 129.1 (C_1_), 129.2 (C_3_), 130.2 (C_4_), 130.3 (d, ^3^*J*_CF_ = 8.1, C_5’_), 138.3 (d, ^3^*J*_CF_ = 7.5, C_1’_), 153.5 (d, ^2^*J*_CP_ = 18.9, PhC=), 162.3 (d, ^1^*J*_CF_ = 247.0, C_3’_); [M + H]^+^_found_ = 446.1969, C_23_H_30_N_3_O_3_FP requires 446.2008.

*Dibutyl [1-(4-fluorobenzyl)-4-phenyl-1,2,3-triazol-5-yl]phosphonate* (**21**): yield: 36% (0.16 g), pale yellow oil; ^31^P NMR (CDCl_3_) δ 2.3; ^1^H NMR (CDCl_3_) δ 0.77 (t, 6H, ^3^*J*_HH_ = 7.3, OCH_2_CH_2_CH_2_C*H*_3_), 1.04–1.19 (m, 4H, OCH_2_CH_2_C*H*_2_CH_3_), 1.22–1.41 (m, 4H, OCH_2_C*H*_2_CH_2_CH_3_), 3.49–3.68 (m, 2H, OC*H*_2_CH_2_CH_2_CH_3_), 3.77–3.96 (m, 2H, OC*H*_2_CH_2_CH_2_CH_3_), 6.00 (s, 2H, CH_2_Ph), 7.03 (t, 2H, ^3^*J*_HH_ = ^3^*J*_HF_ = 8.7, C_2’_H), 7.34–7.47 (m, 5H, C_3_H, C_4_H, C_3’_H), 7.75 (dd, 2H, ^3^*J*_HH_ = 6.7, *J*_HH_ = 3.0, C_2_H); ^13^C NMR (CDCl_3_) δ 13.5 (OCH_2_CH_2_CH_2_*C*H_3_), 18.5 (OCH_2_CH_2_*C*H_2_CH_3_), 32.0 (d, ^3^*J*_CP_ = 7.0, OCH_2_*C*H_2_CH_2_CH_3_), 53.4 (CH_2_Ph), 66.7 (d, ^2^*J*_CP_ = 5.6, O*C*H_2_CH_2_CH_2_CH_3_), 115.6 (d, ^2^*J*_CF_ = 21.7, C_3’_), 121.4 (d, ^1^*J*_CP_ = 218.0, P(O)C=), 128.2 (C_2_), 129.1 (C_1_), 129.2 (C_3_), 130.4 (d, ^3^*J*_CF_ = 8.3, C_2’_), 130.2 (C_4_), 131.7 (d, *J*_CF_ = 3.1, C_1’_), 153.5 (d, ^2^*J*_CP_ = 19.0, PhC=), 162.8 (d, ^1^*J*_CF_ = 247.4, C_4’_); [M + H]^+^_found_ = 446.1972, C_23_H_30_N_3_O_3_FP requires 446.2008.

*Dibutyl [1-(4-trifluoromethyl)-4-phenyl-1,2,3-triazol-5-yl]phosphonate* (**22**): yield: 39% (0.19 g), pale yellow oil; ^31^P NMR (CDCl_3_) δ 2.0; ^1^H NMR (CDCl_3_) δ 0.76 (t, 6H, ^3^*J*_HH_ = 7.3, OCH_2_CH_2_CH_2_C*H*_3_), 0.97–1.18 (m, 4H, OCH_2_CH_2_C*H*_2_CH_3_), 1.20–1.40 (m, 4H, OCH_2_C*H*_2_CH_2_CH_3_), 3.53–3.67 (m, 2H, OC*H*_2_CH_2_CH_2_CH_3_), 3.77–3.95 (m, 2H, OC*H*_2_CH_2_CH_2_CH_3_), 6.10 (s, 2H, CH_2_Ph), 7.37–7.46 (m, 3H, C_3_H, C_4_H), 7.51 (d, 2H, ^3^*J*_HH_ = 8.0, C_2’_H), 7.61 (d, 2H, ^3^*J*_HH_ = 8.1, C_3’_H), 7.76 (dd, 2H, ^3^*J*_HH_ = 6.8, *J*_HH_ = 2.9, C_2_H); ^13^C NMR (CDCl_3_) δ 13.4 (OCH_2_CH_2_CH_2_*C*H_3_), 18.5 (OCH_2_CH_2_*C*H_2_CH_3_), 31.9 (d, ^3^*J*_CP_ = 7.0, OCH_2_*C*H_2_CH_2_CH_3_), 53.6 (CH_2_Ph), 66.8 (d, ^2^*J*_CP_ = 5.7, O*C*H_2_CH_2_CH_2_CH_3_), 124.0 (q, ^1^*J*_CF_ = 272.2, CF_3_), 121.7 (d, ^1^*J*_CP_ = 218.2, P(O)C=), 125.7 (q, ^3^*J*_CF_ = 3.8, C_3’_), 128.2 (C_2_), 128.7 (C_3_), 129.2 (C_1_, C_2’_), 130.1 (C_4_), 130.7 (q, ^2^*J*_CF_ = 32.6, C_4’_), 139.9 (C_1’_), 153.6 (d, ^2^*J*_CP_ = 18.9, PhC=); [M + H]^+^_found_ = 496.1936, C_24_H_30_N_3_O_3_F_3_P requires 496.1976.

*Dimethyl (1-octyl-4-phenyl-1,2,3-triazol-5-yl)phosphonate* (**23**): yield: 43% (0.16 g), colorless oil; ^31^P NMR (CDCl_3_) δ 8.0; ^1^H NMR (CDCl_3_) δ 0.81 (t, 3H, ^3^*J*_HH_ = 6.9, NCH_2_(CH_2_)_6_C*H*_3_), 1.12–1.37 (m, 10H, NCH_2_CH_2_(C*H*_2_)_5_C*H*_3_), 1.82–2.02 (m, 2H, NCH_2_C*H*_2_(CH_2_)_5_CH_3_), 3.58 (d, 6H, ^3^*J*_HP_ = 11.6, OCH_3_), 4.68 (t, 2H, ^3^*J*_HH_ = 7.5, NC*H*_2_(CH_2_)_6_CH_3_), 7.29–7.45 (m, 3H, C_3_H, C_4_H), 7.65 (dd, 2H, ^3^*J*_HH_ = 6.5, *J*_HH_ = 3.3, C_2_H); ^13^C NMR (CDCl_3_) δ 14.2 (NCH_2_(CH_2_)_6_*C*H_3_), 22.7 (N(CH_2_)_6_*C*H_2_CH_3_), 26.7 (N(CH_2_)_5_*C*H_2_CH_2_CH_3_), 29.16 (N(CH_2_)_3_CH_2_*C*H_2_(CH_2_)_2_CH_3_), 29.20 (N(CH_2_)_3_*C*H_2_CH_2_(CH_2_)_2_CH_3_), 31.1 (N(CH_2_)_2_*C*H_2_(CH_2_)_4_CH_3_), 31.8 (NCH_2_*C*H_2_(CH_2_)_5_CH_3_), 51.4 (N*C*H_2_CH_2_(CH_2_)_5_CH_3_), 53.2 (d, ^2^*J*_CP_ = 5.6, OCH_3_), 120.6 (d, ^1^*J*_CP_ = 221.5, P(O)C=), 128.2 (C_2_), 129.1 (C_1_), 129.3 (C_3_), 130.2 (C_4_), 153.5 (d, ^2^*J*_CP_ = 20.1, PhC=); [M + H]^+^_found_ = 366.1930, C_18_H_29_N_3_O_3_P requires 366.1946.

*Diethyl (1-octyl-4-phenyl-1,2,3-triazol-5-yl)phosphonate* (**24**): yield: 58% (0.23 g), colorless oil; ^31^P NMR (CDCl_3_) δ 5.0; ^1^H NMR (CDCl_3_) δ 0.88 (t, 3H, ^3^*J*_HH_ = 6.6, NCH_2_(CH_2_)_6_C*H*_3_), 1.16 (t, 6H, ^3^*J*_HH_ = 7.1, OCH_2_CH_2_CH_2_C*H*_3_), 1.22–1.47 (m, 10H, NCH_2_CH_2_(C*H*_2_)_5_C*H*_3_), 1.93–2.06 (m, 2H, NCH_2_C*H*_2_(CH_2_)_5_CH_3_), 3.83–4.01 (m, 2H, OC*H*_2_CH_3_), 4.01–4.19 (m, 2H, OC*H*_2_CH_3_), 4.76 (t, 2H, ^3^*J*_HH_ = 7.5, NC*H*_2_(CH_2_)_6_CH_3_), 7.34–7.48 (m, 3H, C_3_H, C_4_H), 7.75 (dd, 2H, ^3^*J*_HH_ = 6.4, *J*_HH_ = 2.8, C_2_H); ^13^C NMR (CDCl_3_) δ 14.2 (NCH_2_(CH_2_)_6_*C*H_3_), 16.0 (d, ^3^*J*_CP_ = 7.0, OCH_2_*C*H_3_), 22.7 (N(CH_2_)_6_*C*H_2_CH_3_), 26.7 (N(CH_2_)_5_*C*H_2_CH_2_CH_3_), 29.19 (N(CH_2_)_3_CH_2_*C*H_2_(CH_2_)_2_CH_3_), 29.21 (N(CH_2_)_3_*C*H_2_CH_2_(CH_2_)_2_CH_3_), 31.1 (N(CH_2_)_2_*C*H_2_(CH_2_)_4_CH_3_), 31.8 (NCH_2_*C*H_2_(CH_2_)_5_CH_3_), 51.3 (N*C*H_2_CH_2_(CH_2_)_5_CH_3_), 63.1 (d, ^2^*J*_CP_ = 5.5, O*C*H_2_CH_3_), 121.7 (d, ^1^*J*_CP_ = 220.2, P(O)C=), 128.1 (C_2_), 129.0 (C_1_), 129.5 (C_3_), 130.5 (C_4_), 153.2 (d, ^2^*J*_CP_ = 20.0, PhC=); [M + H]^+^_found_ = 394.2241, C_20_H_33_N_3_O_3_P requires 394.2259.

*Dibutyl (1-octyl-4-phenyl-1,2,3-triazol-5-yl)phosphonate* (**25**): yield: 50% (0.22 g), colorless oil; ^31^P NMR (CDCl_3_) δ 5.3; ^1^H NMR (CDCl_3_) δ 0.75–0.97 (m, 9H, OCH_2_CH_2_CH_2_C*H*_3_, NCH_2_(CH_2_)_6_C*H*_3_), 1.13–1.55 (m, 18H, OCH_2_C*H*_2_C*H*_2_CH_3_, NCH_2_CH_2_(C*H*_2_)_5_CH_3_), 1.89–2.07 (m, 2H, NCH_2_C*H*_2_(CH_2_)_5_CH_3_), 3.75–3.95 (m, 2H, OC*H*_2_CH_2_CH_2_CH_3_), 3.95–4.14 (m, 2H, OC*H*_2_CH_2_CH_2_CH_3_), 4.77 (t, 2H, ^3^*J*_HH_ = 7.4, NC*H*_2_(CH_2_)_6_CH_3_), 7.34–7.51 (m, 3H, C_3_H, C_4_H), 7.74 (dd, 2H, ^3^*J*_HH_ = 6.7, *J*_HH_ = 3.1, C_2_H); ^13^C NMR (CDCl_3_) δ 13.6 (OCH_2_CH_2_CH_2_*C*H_3_), 14.2 (NCH_2_(CH_2_)_6_*C*H_3_), 18.7 (OCH_2_CH_2_*C*H_2_CH_3_), 22.8 (N(CH_2_)_6_*C*H_2_CH_3_), 26.7 (N(CH_2_)_5_*C*H_2_CH_2_CH_3_), 29.3 (N(CH_2_)_3_*C*H_2_*C*H_2_(CH_2_)_2_CH_3_), 29.8 (N(CH_2_)_2_*C*H_2_(CH_2_)_4_CH_3_), 31.2 (N(CH_2_*C*H_2_(CH_2_)_5_CH_3_), 32.2 (d, ^3^*J*_CP_ = 6.9, OCH_2_*C*H_2_CH_2_CH_3_), 51.4 (N(*C*H_2_CH_2_(CH_2_)_5_CH_3_), 66.8 (d, ^2^*J*_CP_ = 5.8, O*C*H_2_CH_2_CH_2_CH_3_), 121.6 (d, ^1^*J*_CP_ = 220.2, P(O)C=), 128.2 (C_2_), 129.0 (C_1_), 129.4 (C_3_), 130.5 (C_4_), 153.2 (d, ^2^*J*_CP_ = 19.9, PhC=); [M + H]^+^_found_ = 450.2862, C_24_H_41_N_3_O_3_P requires 450.2885.

*Dibutyl (1-isooctyl-4-phenyl-1,2,3-triazol-5-yl)phosphonate* (**26**): yield: 28% (0.13 g), colorless oil; ^31^P NMR (CDCl_3_) δ 3.0; ^1^H NMR (CDCl_3_) δ 0.77–0.96 (m, 12H, OCH_2_CH_2_CH_2_C*H*_3_, NCH_2_CHCH_2_C*H*_3_, NCH_2_CH(CH_2_)_3_C*H*_3_), 1.16–1.53 (m, 16H, OCH_2_C*H*_2_C*H*_2_CH_3_, NCH_2_CHC*H*_2_CH_3_, NCH_2_CH(C*H*_2_)_3_CH_3_), 2.06–2.20 (m, 1H, NCH_2_C*H*CH_2_CH_3_), 3.77–3.94 (m, 2H, OC*H*_2_CH_2_CH_2_CH_3_), 3.97–4.11 (m, 2H, OC*H*_2_CH_2_CH_2_CH_3_), 4.69 (d, 2H, ^3^*J*_HH_ = 7.5, NC*H*_2_CHCH_2_CH_3_), 7.34–7.45 (m, 3H, C_3_H, C_4_H), 7.75 (dd, 2H, ^3^*J*_HH_ = 6.4, *J*_HH_ = 3.3, C_2_H); ^13^C NMR (CDCl_3_) δ 10.5 (NCH_2_CHCH_2_*C*H_3_), 13.5 (OCH_2_CH_2_CH_2_*C*H_3_), 14.1 (NCH_2_CH(CH_2_)_3_*C*H_3_), 18.7 (OCH_2_CH_2_*C*H_2_CH_3_), 23.0 (NCH_2_CH(CH_2_)_2_*C*H_2_CH_3_), 23.7 (NCH_2_CH*C*H_2_CH_3_), 28.5 (NCH_2_CHCH_2_*C*H_2_CH_2_CH_3_), 30.4 (NCH_2_CH*C*H_2_(CH_2_)_2_CH_3_), 32.1 (d, ^3^*J*_CP_ = 6.9, OCH_2_*C*H_2_CH_2_CH_3_), 40.5 (NCH_2_*C*HCH_2_CH_3_), 54.8 (N*C*H_2_CHCH_2_CH_3_), 66.7 (d, ^2^*J*_CP_ = 5.8, O*C*H_2_CH_2_CH_2_CH_3_), 121.9 (d, ^1^*J*_CP_ = 221.1, P(O)C=), 128.1 (C_2_), 128.9 (C_1_), 129.4 (C_3_), 130.5 (C_4_), 153.2 (d, ^2^*J*_CP_ = 19.8, PhC=); [M + H]^+^_found_ = 450.2855, C_24_H_41_N_3_O_3_P requires 450.2885.

### 3.5. Biological Evaluation 

#### 3.5.1. Cell Culture

Cells were purchased from the American Type Culture Collection (ATCC, Manassas, VA, USA). Human lung adenocarcinoma A549 cells were maintained in Dulbecco's Modified Eagle Medium (DMEM), while mouse fibroblast NIH/3T3 and human promyelocytic leukemia HL-60 cells were maintained in Roswell Park Memorial Institute 1640 medium (RPMI-1640) containing 10% FCS. Media were supplemented with 2 mM GlutaMAX, 100 U/mL penicillin, and 100 µg/mL streptomycin (Life Technologies, Carlsbad, California, USA). Cell cultures were maintained at 37 °C in a humidified incubator in an atmosphere of 5% CO_2_ (Sanyo, Japan).

#### 3.5.2. Antibacterial Activity

All reagents were purchased from Sigma-Aldrich (Hungary, Budapest). *E. coli-GFP* and *B. subtilis-GFP* bacteria were grown in Lysogeny Broth (Luria-Bertani broth, LB, 10 g tryptone, 5 g yeast extract, 10 g/L NaCl, pH 7.0) medium overnight (ON) at 37 °C in an incubator with continuous shaking [44,45]. The ON bacterial culture was diluted (*E. coli-GFP:* 1:10000, *B. subtilis-GFP*: 1:1000) in LB medium containing 10 µg/mL ampicillin. Two hundred microliters of diluted *E. coli* suspension per well were transferred to 96-well plates and different agents were added in the appropriate concentration, then incubated in a shaker (600 rpm) for 3 h at 37 °C. Test compounds were dissolved in 3-fold amounts of dimethyl sulfoxide (DMSO). Cells were treated with an increasing concentration of test compounds (1–30 µM). Doxycyclin (IC_50_ = 0.10 ± 0.02 and 0.04 ± 0.01 µM for *E. coli* and *B. subtilis*) and gentamicin (IC_50_ = 4.23 ± 0.99 and 0.49 ± 0.14 µM for *E. coli* and *B. subtilis*) were used as positive controls. For induction of the GFP expression IPTG (0.1 mM final concentration) was added to each well and incubated for 3 h at 37 °C.

The following incubation samples were centrifuged at 2750 rpm at room temperature (RT) for 5 min and washed twice with 200 µL PBS, with a centrifugation step between the two washes. After a third centrifugation step, the bacterial cells were suspended in 100 µL PBS. The number of bacteria was estimated according to the fluorimetric measurements. The fluorescence of the bacterial cells was measured by the Victor™ 1420 Multilabel Counter (Perkin Elmer, Waltham, MA, USA) at 485/535 nm for 1 sec per well controlled by Wallac 1420 Manager software. Each treatment was repeated in at least 2 wells per plate during the experiments. Percentage fluorescence intensity based on the quantity of *E. coli-GFP* cells was calculated using untreated control values as 100%. Error was represented by standard deviation (SD). IC_50_ values (50% inhibiting concentration) were calculated by GraphPad Prism^®^ 5 (La Jolla, CA, USA).

#### 3.5.3. Cytotoxicity Assay

Cytotoxicity of the synthesized molecules was determined on A549, NIH/3T3 and HL-60 cells using the fluorescent Resazurin assay as described previously [46].

Briefly, cells (A549 and NIH/3T3: 6000, HL-60: 120.000 cells/well) were seeded into 96-well plates (Corning Life Sciences) in media and incubated overnight. Test compounds were dissolved in three-fold amounts of dimethyl sulfoxide (DMSO). Cells were treated with an increasing concentration of test compounds (1–30 µM). Positive controls were doxorubicin for A549 and NIH/3T3 (IC_50_ = 0.31 ± 0.24 µM and 5.65 ± 0.81 µM, respectively), and bortezomib for HL60 (IC_50_ = 7.42 ± 2.60 nM). Cell viability was determined after 72 h incubation. Resazurin reagent (Sigma-Aldrich) was added at a final concentration of 25 µg/mL. After a 2 h incubation at 37 °C in 5% CO_2_, fluorescence (530 nm excitation/580 nm emission) was recorded on a multimode microplate reader (Cytofluor4000, PerSeptive Biosytems). Viability was calculated with relation to untreated control cells and blank wells containing media without cells. IC_50_ values (50% inhibiting concentration) were calculated by GraphPad Prism^®^ 5. A Student’s t test was performed for individual test compound concentrations with relation to untreated control cell viability values.

## 4. Conclusions

In summary, the synthesis of 1,2,3-triazol-5-yl-phosphonates was studied by the copper(I)-chloride-catalyzed three-component domino reaction of phenylacetylene, azides and dialkyl phosphites. The model reaction of phenylacetylene, benzyl azide and dibutyl phosphite was optimized in details. The effect of the atmosphere, the type of base, the catalyst and the solvent, as well as the molar ratio of the starting materials, the temperature and the reaction time were studied. Applying the approach developed, altogether 17 triazol-5-yl-phosphonate derivatives were synthesized and fully characterized, except two (**12** and **14**), all of them are new compounds. The biological activity of the compounds prepared was investigated in antibacterial activity and in vitro cytotoxicity assays. None of the synthesized 1,2,3-triazol-5-yl-phosphonates were active against selected Gram-negative bacteria, while the growth of Gram-positive bacteria was somewhat reduced. Several derivatives showed low or modest activity against the tested cell lines, two of them have the IC_50_ value in the 10 micromolar range against HL-60 cells.

## Figures and Tables

**Table 1:**
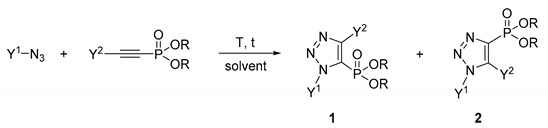
Cycloaddition of azides and alkynyl phosphonates.

Entry	Y^1^	Y^2^	R	Cat.	Solvent	T, t	Product Ratio (%)		Yield (%)	Ref.
							1	2		
**1**	CH_2_COOMe	COOEt	Et	-	toluene	110 °C	64	36	-	[28]
**2**	CH_2_COO*^t^*Bu	CF_3_	*^i^*Pr	-	DEE	25 °C, 20 h	75	25	90	[29]
**3**	Bn	Ph, 4-MeC_6_H_4_, 4-FC_6_H_4_, etc.	Et	CuSO_4_·5H_2_O/NaAsc	DMF	170 °C, 12 h	100	0	83–92	[30]
**4**	(EtO)_2_P(O)(CH_2_)_2_	(MeO)_2_P(O)	Me	-	water	25 °C, 36 h or 60 °C, 2 h	100		95	[31]
**5**	(EtO)_2_P(O)CH_2_	Me	Et	-	-	MW, 90 °C, 20 min	34	66	78	[32]

**Table 2:**
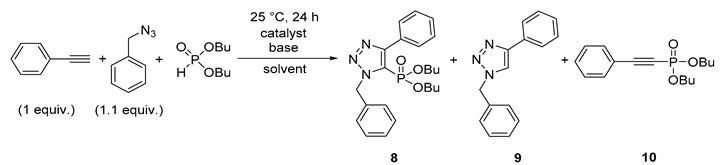
Optimization of the domino reaction of phenylacetylene, benzyl azide and dibutyl phosphite.

Entry	DBP (Equiv.)	Condition	Catalyst (10 mol%)	Base (2 Equiv.)	Solvent	Product Composition (%) ^a^		
						8	9	10
**1**	1.1	N_2_ bubbling	CuCl	TEA	MeCN	22	77	1
**2**	1.1	air bubbling	CuCl	TEA	MeCN	55	42	3
**3**	1.1	O_2_ bubbling	CuCl	TEA	MeCN	24	70	6
**4**	1.1	air bubbling	CuCl	DPA	MeCN	46	49	5
**5**	1.1	air bubbling	CuCl	DIPA	MeCN	54	44	2
**6**	1.1	air bubbling	CuCl	DIPEA	MeCN	35	63	2
**7**	1.1	air bubbling	CuCl	TEA	DMF	41	57	2
**8**	1.1	air bubbling	CuCl	TEA	DCM	43	56	1
**9**	1.1	air bubbling	CuCl	TEA	THF	21	78	1
**10**	1.1	air bubbling	CuCl	TEA	EtOH	39	59	2
**11**	1.1	air bubbling	CuBr	TEA	MeCN	42	56	2
**12**	1.1	air bubbling	CuI	TEA	MeCN	26	72	2
**13**	1.1	air bubbling	CuSO_4_·5H_2_O/NaAsc	TEA	MeCN	0	100	0
**14**	2	air bubbling	CuCl	TEA	MeCN	59	37	4
**15**	3	air bubbling	CuCl	TEA	MeCN	51	45	4
**16 ^b^**	2	air bubbling	CuCl	TEA	MeCN	49	49	2
**17 ^c^**	2	air bubbling	CuCl	TEA	MeCN	36	62	2
**18 ^d^**	2	air bubbling	CuCl	TEA	MeCN	23	75	2

^a^ Based on HPLC (222 nm). ^b^ The reaction was performed using 15 mol% of CuCl. ^c^ The reaction was carried out at 0 °C. ^d^ The reaction was carried out at 50 °C.

**Table 3:**
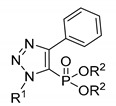
Summary of biological activity measurements.

Compound	R^1^	R^2^	Antibacterial Activity (IC_50_, µM)		In Vitro Cytotoxicity (IC_50_, µM)		
			*B. subtilis*	*E. coli*	A549	NIH/3T3	HL-60
**8**	Bn	Bu	>30	>30	>30	27.5 ± 1.4	>30
**11**	Bn	Me	>30	>30	>30	>30	>30
**12**	Bn	Et	>30	>30	>30	>30	>30
**13**	Bn	Pr	29.3 ± 1.2	>30	>30	>30	12.6 ± 1.7
**14**	Bn	*^i^*Pr	>30	>30	>30	>30	>30
**15**	Bn	Pn	23.9 ± 1.0	>30	>30	26.2 ± 1.1	15.4 ± 1.2
**16**	4-MeC_6_H_4_	Me	>30	>30	>30	19.8 ± 1.2	11.0 ± 1.2
**17**	4-MeC_6_H_4_	Et	>30	>30	>30	>30	>30
**18**	4-MeC_6_H_4_	Bu	18.2 ± 1.0	>30	>30	>30	>30
**19**	2-FC_6_H_4_	Bu	19.3 ± 1.0	>30	>30	27.5 ± 1.1	11.7 ± 1.2
**20**	3-FC_6_H_4_	Bu	>30	>30	>30	>30	15.4 ± 1.2
**21**	4-FC_6_H_4_	Bu	23.2 ± 1.1	>30	>30	>30	16.6 ± 1.3
**22**	4-CF_3_C_6_H_4_	Bu	>30	>30	>30	23.1 ± 1.2	9.7 ± 1.1
**23**	Oct	Me	>30	>30	>30	>30	>30
**24**	Oct	Et	>30	>30	>30	>30	>30
**25**	Oct	Bu	>30	>30	>30	>30	>30
**26**	*^i^*Oct	Bu	29.6 ± 1.0	>30	>30	>30	26.7 ± 1.0
Doxycycline			0.04 ± 0.01	0.10 ± 0.02	–	–	–
Gentamicin			0.49 ± 0.14	4.23 ± 0.99	–	–	–
Doxorubicin			–	–	0.31 ± 0.24	5.65 ± 0.81	–
Bortezomib			–	–	–	–	7.42 × 10^−3^ 2.60 × 10^−3^

Data were expressed as mean ± standard deviation.

**Table 4:** Yield and mass data for the organic azides prepared.

R^1^N_3_	Yield	[M + H]^+^_found_	[M + H]^+^_requires_
BnN_3_ [36]	93% (1.24 g)	134.0725	134.0718
4-Me-BnN_3_ [37]	80% (1.18 g)	148.0880	148.0874
2-F-BnN_3_ [38]	68% (1.02 g)	152.0632	152.0624
3-F-BnN_3_ [39]	76% (1.14 g)	152.0633	152.0624
4-F-BnN_3_ [37]	83% (1.25 g)	152.0632	152.0624
4-CF_3_-BnN_3_ [40]	86% (1.72 g)	202.0601	202.0592
OctN_3_ [41]	67% (1.04 g)	156.1514	156.1501
*^i^*OctN_3_ [42]	55% (0.85 g)	156.1514	156.1501

**Table 5:** Yield, ^31^P NMR and MS data for the dialkyl phosphites prepared.

(R^2^O)_2_P(O)H	Yield	δ_P_ in CDCl_3_	δ_P_ [lit.] in CDCl_3_	[M + H]^+^_found_	[M + H]^+^_requires_
(PrO)_2_P(O)H	65% (0.54 g)	7.9	7.8 [43]	167.0833	167.0837
(PentO)_2_P(O)H	68% (0.75 g)	7.8	8.1 [43]	223.1462	223.1463

## Supplementary Materials

Supplementary data associated with this article are available online. Copies of ^31^P, ^1^H, and ^13^C NMR spectra for all compounds synthesized are presented.
