# Supplementary material for: Synthesis and In Vitro Cytotoxicity and Antibacterial Activity of Novel 1,2,3-Triazol-5-yl-Phosphonates
Source: Molecules. 2020 Jun 6;25(11):2643. doi: 10.3390/molecules25112643 (PMC7321403; doi:10.3390/molecules25112643)
Supplement: Supplementary file 1 [file molecules-25-02643-s001.pdf]

Supplementary Material

for

Synthesis and anticancer cytotoxicity of 1,2,3-triazol-5-yl phosphonates

Anna Tripolszky [1], Emese Tóth [1], Pál Tamás Szabó [2], László Hackler Jr.[3], Beáta Kari [3], László G. Puskás[3]* and Erika Bálint [1]*

Address:

[1] Department of Organic Chemistry and Technology, Budapest University of Technology and Economics, H-1521 Budapest, Hungary
[2] MS Metabolomics Laboratory, Instrumentation Center, Research Centre for Natural Sciences, Hungarian Academy of Sciences, Magyar tudósok krt. 2., H-1117 Budapest, Hungary
[3] Avidin Ltd., Alsó kikötő sor 11/D, H-6726 Szeged, Hungary

E-mail:

Erika Bálint* - ebalint@mail.bme.hu

László G. Puskás* - laszlo@avidinbiotech.com

* Corresponding author

Table of contents

^{31}P NMR, ^{1}H NMR and ^{13}C NMR spectra S2–S27

^{31}P NMR, ^{13}C NMR and ^{1}H NMR spectra of compounds

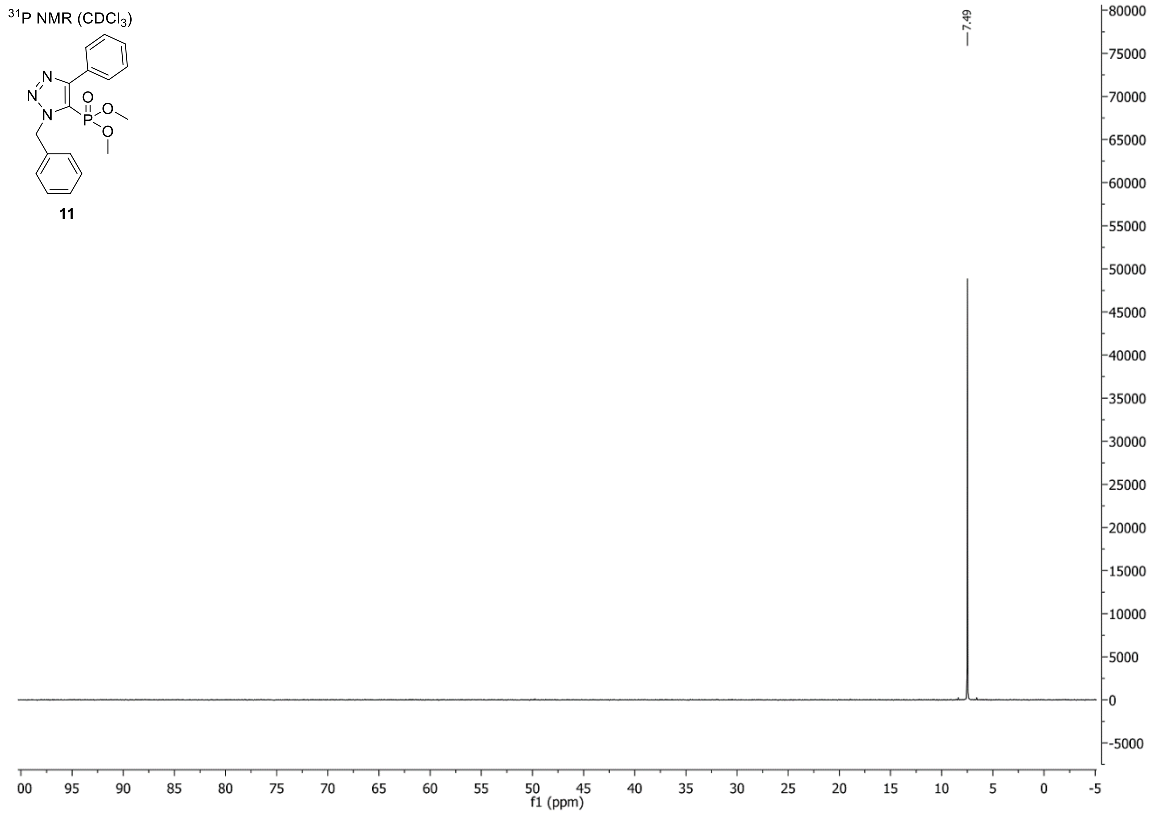

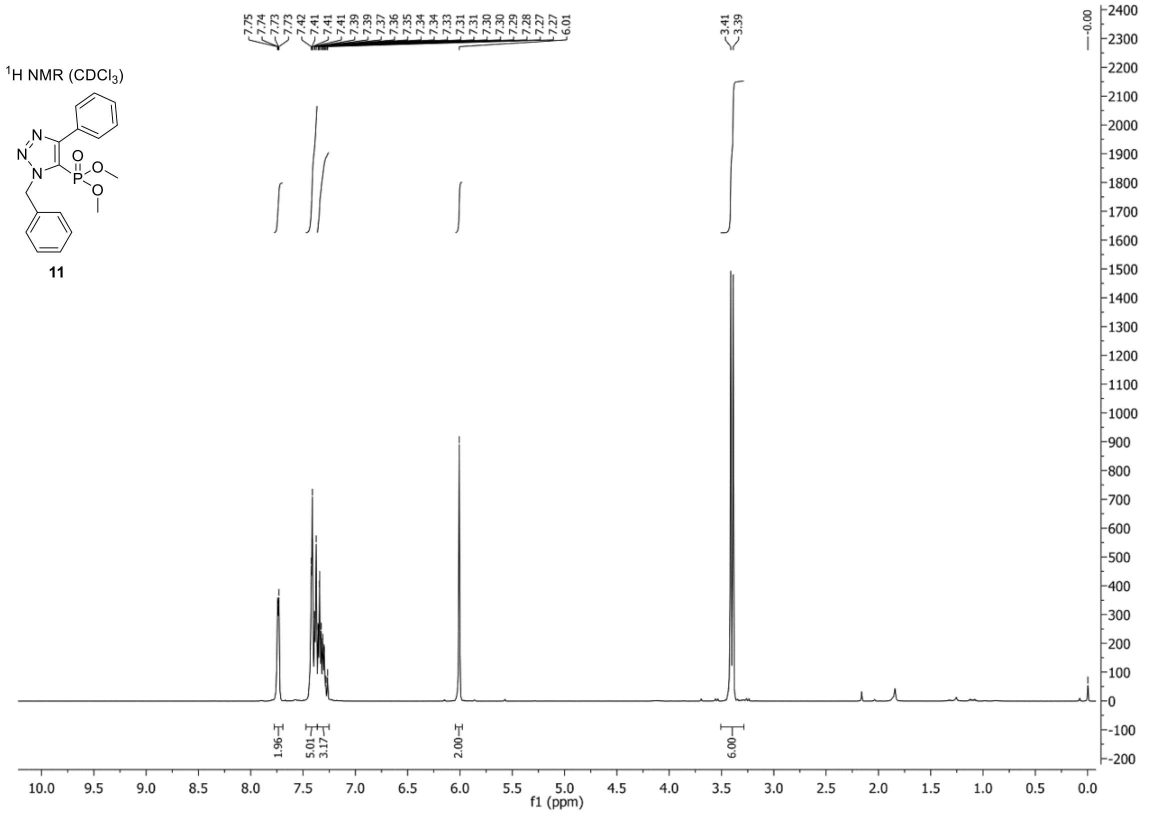

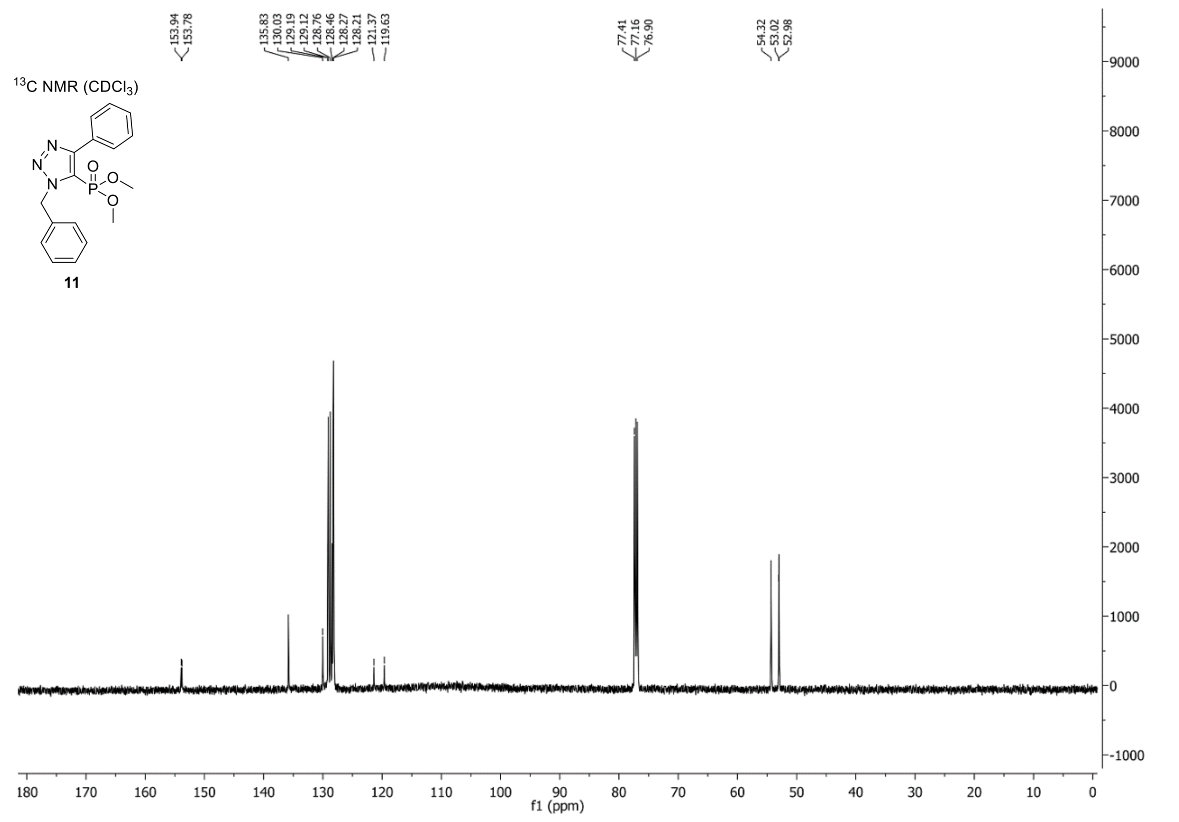

153.94
153.78
135.83
130.03
129.19
129.12
128.76
128.46
128.27
128.21
121.37
119.63
77.41
77.16
76.90
54.32
53.02
52.98
^{13}C NMR (CDCl$_3$)
11
f1 (ppm)

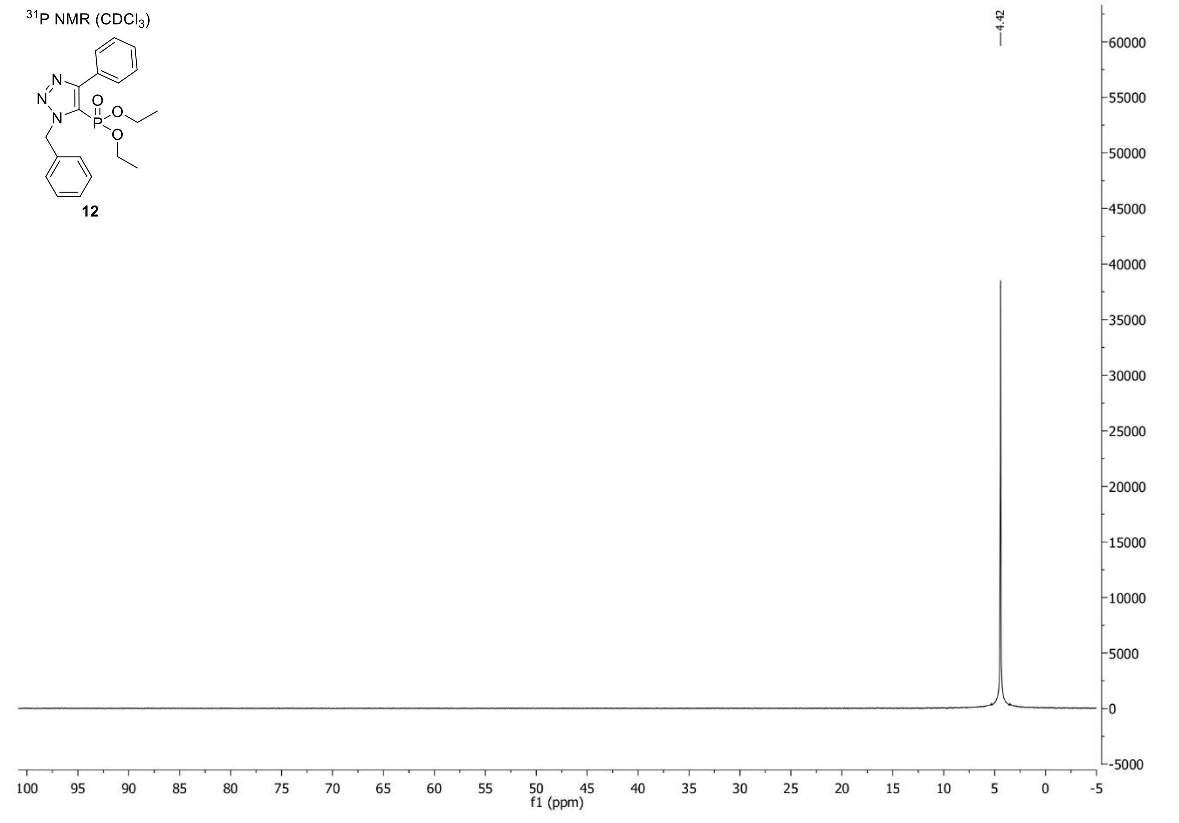

4.42
^{31}P NMR (CDCl$_3$)
12
f1 (ppm)

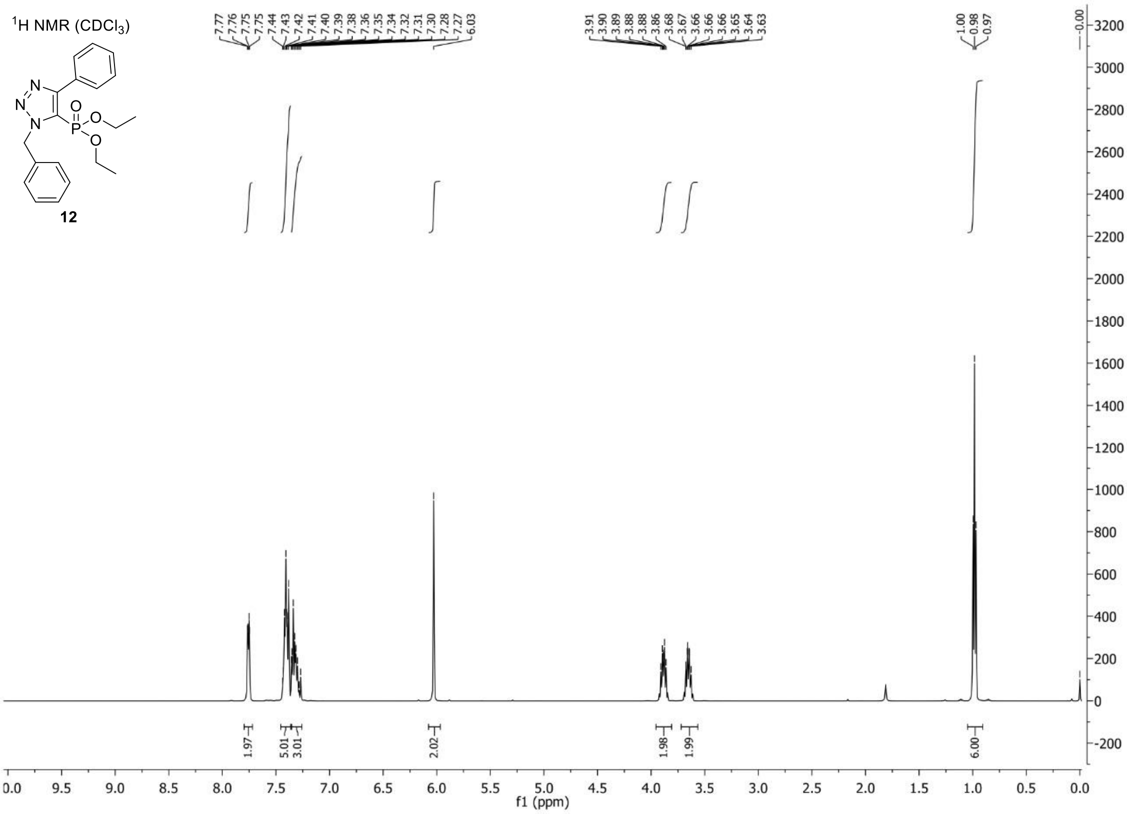

^{1}H NMR ($CDCl_3$)
12
7.77 7.76 7.75 7.75 7.44 7.43 7.42 7.41 7.40 7.39 7.38 7.36 7.35 7.34 7.32 7.31 7.30 7.28 7.27 6.03
3.91 3.90 3.89 3.88 3.88 3.86 3.68 3.67 3.66 3.66 3.65 3.64 3.63
1.00 0.98 0.97
-0.00
1.97 5.01 3.01 2.02 1.98 1.99 6.00
f1 (ppm)

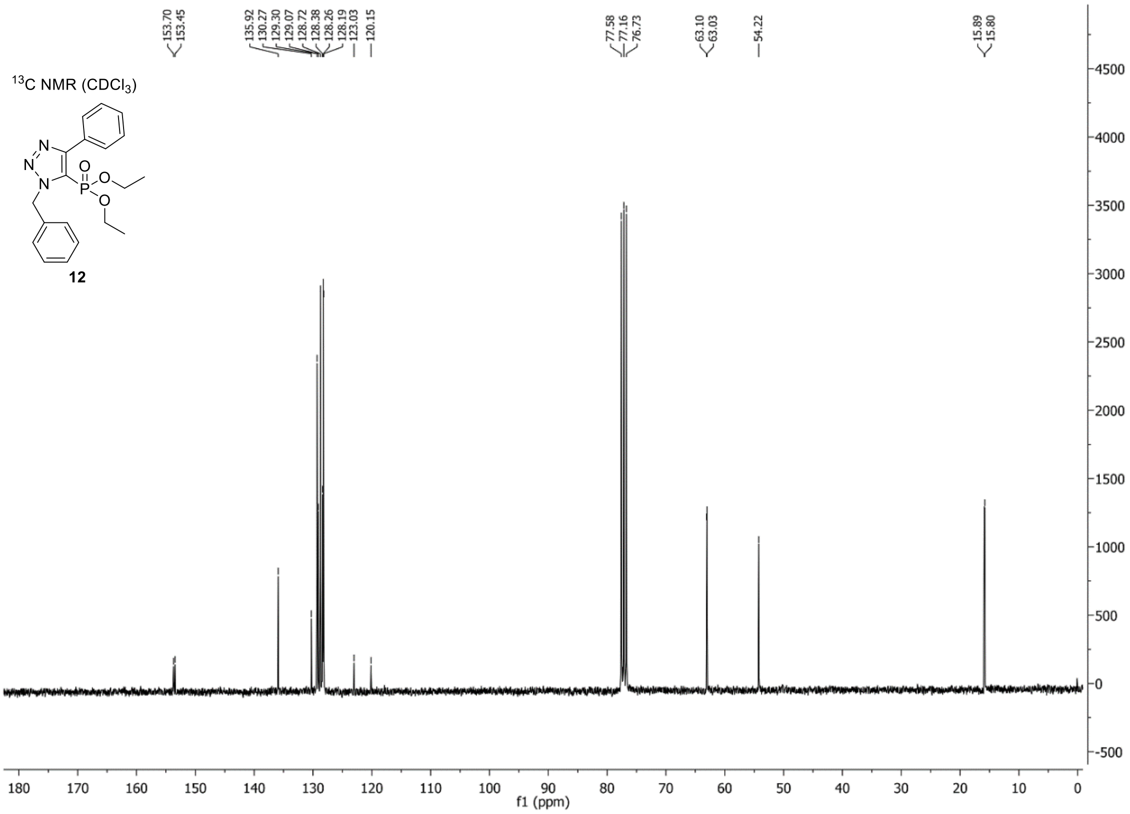

^{13}C NMR ($CDCl_3$)
12
153.70 153.45
135.92 130.27 129.30 129.07 128.72 128.38 128.26 128.19 123.03 120.15
77.58 77.16 76.73
63.10 63.03
54.22
15.89 15.80
f1 (ppm)

^{31}P NMR ($CDCl_3$)

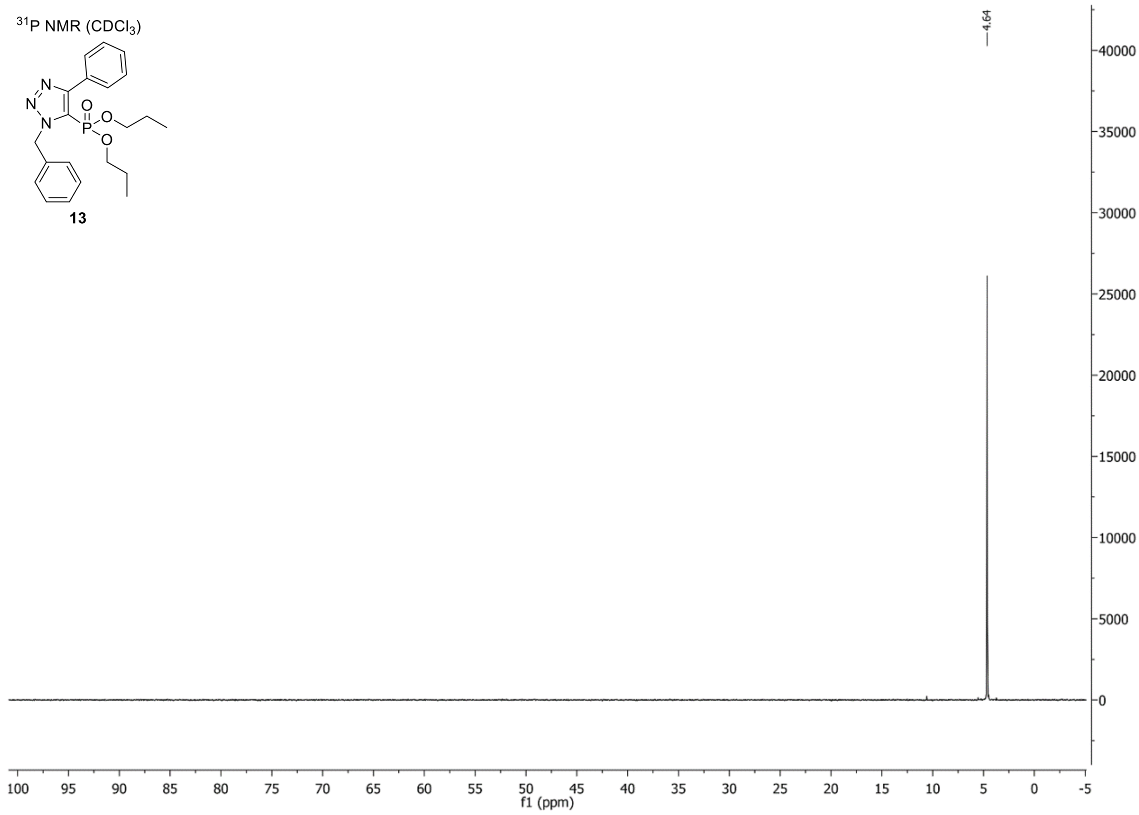

13

^{1}H NMR ($CDCl_3$)

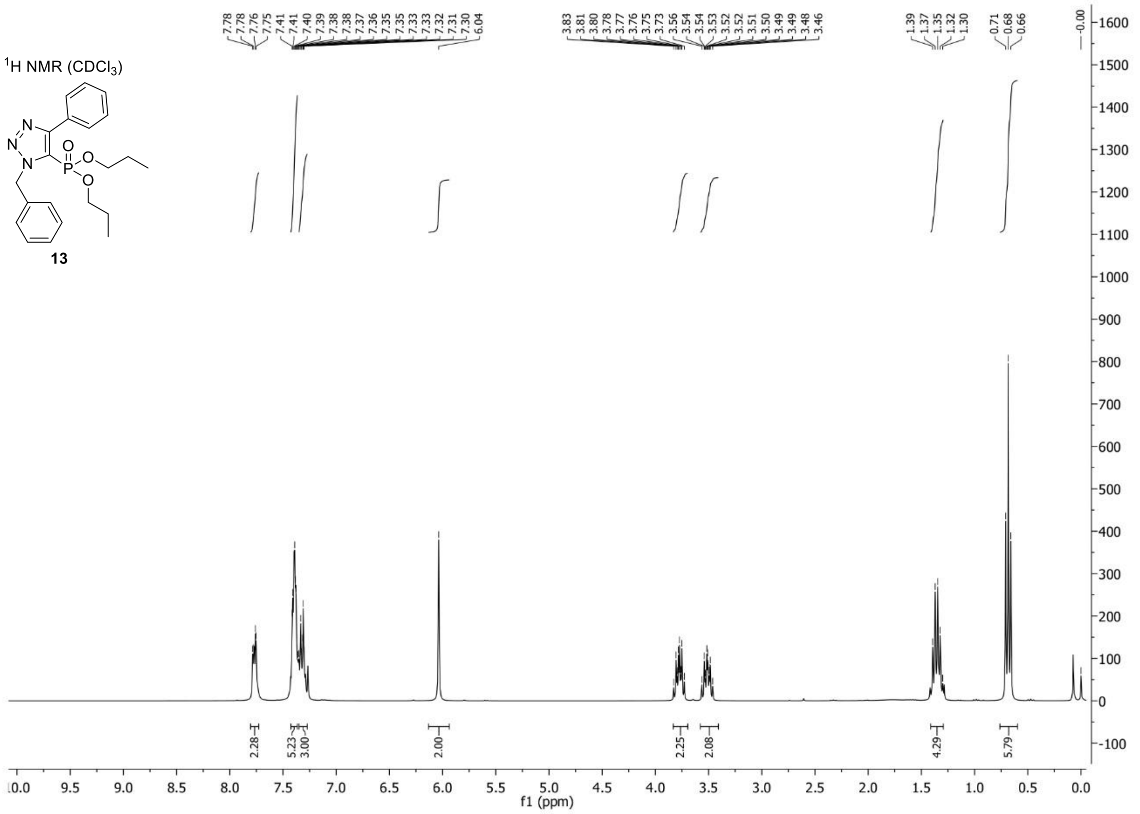

13

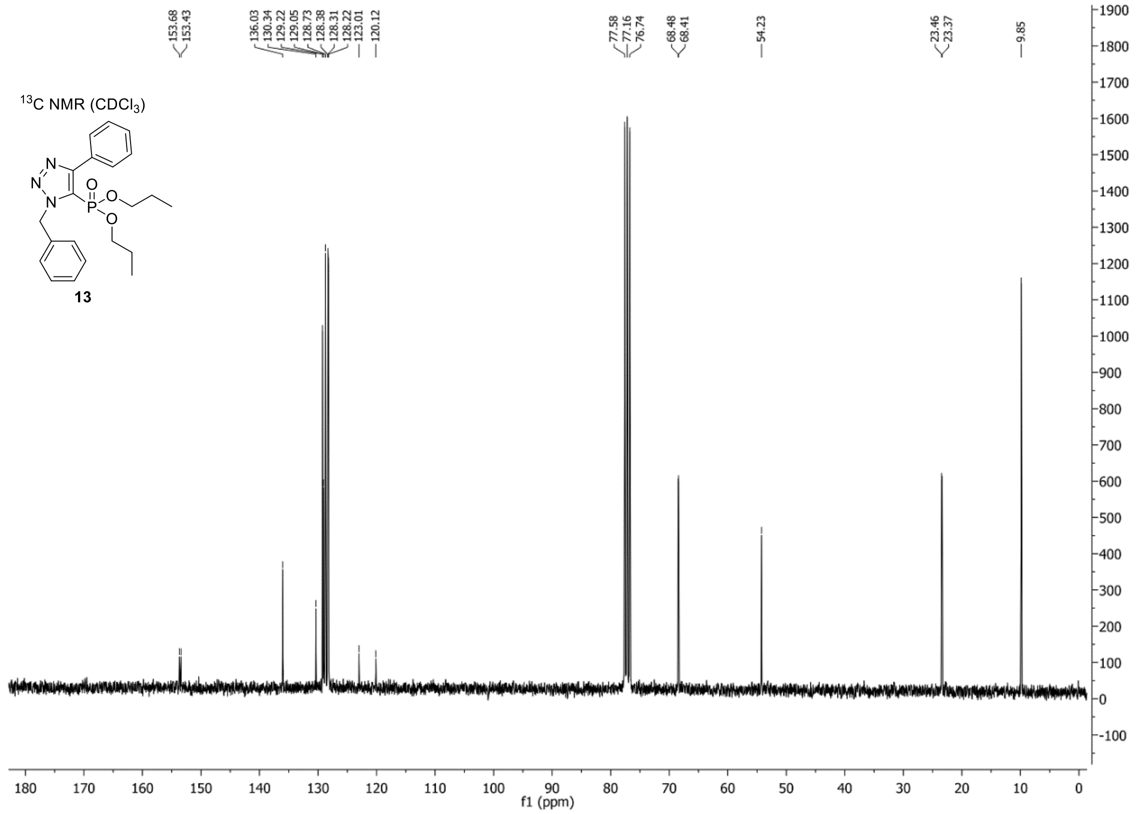
13C NMR (CDCl3)
13
153.68
153.43
136.03
130.34
129.22
129.05
128.73
128.38
128.31
128.22
123.01
120.12
77.58
77.16
76.74
68.48
68.41
54.23
23.46
23.37
9.85
f1 (ppm)

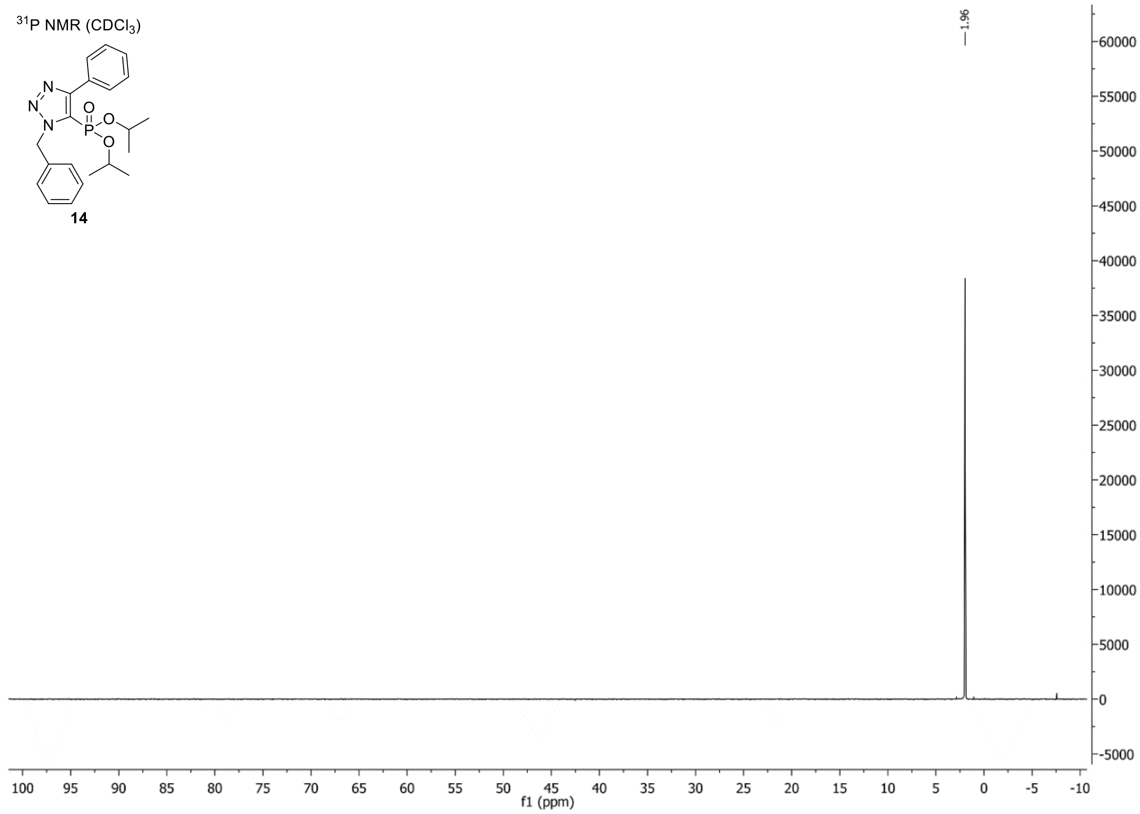
31P NMR (CDCl3)
14
1.96
f1 (ppm)

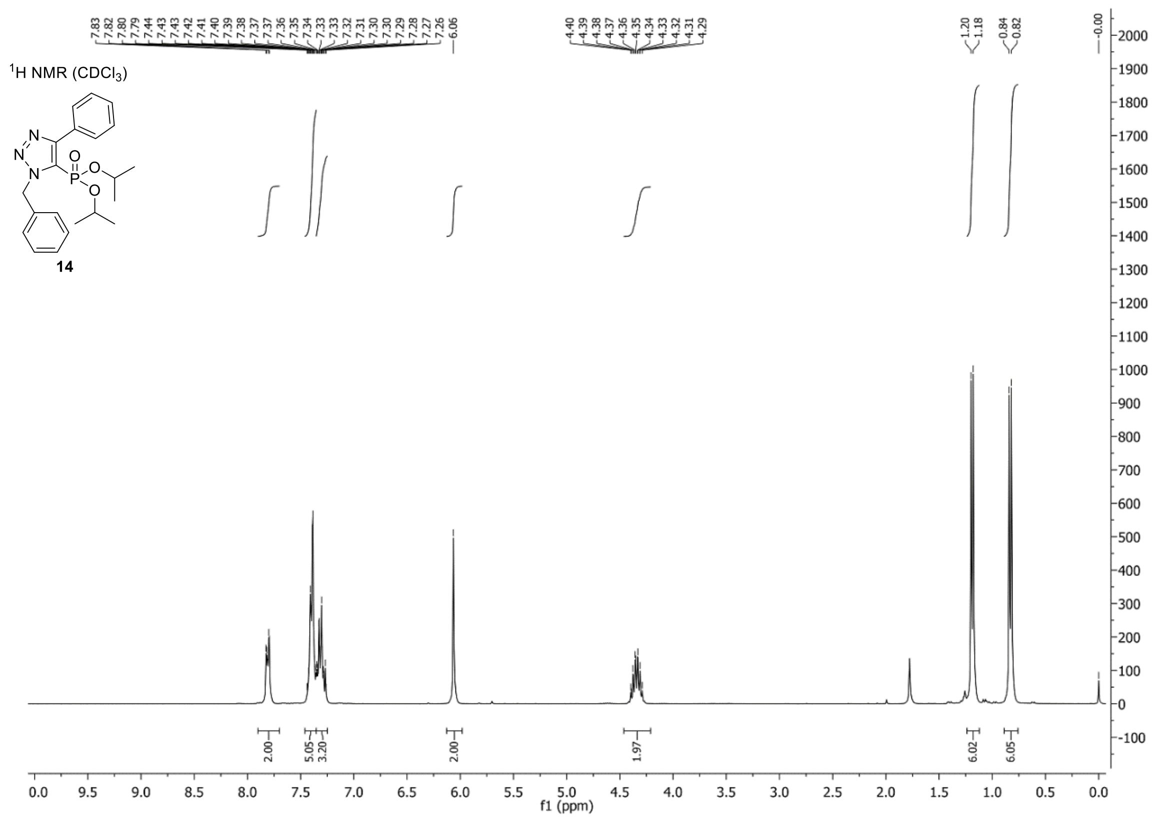

^{1}H NMR ($CDCl_3$)
14
7.83 7.82 7.80 7.79 7.44 7.43 7.43 7.42 7.41 7.40 7.39 7.38 7.37 7.37 7.36 7.35 7.34 7.33 7.33 7.33 7.32 7.31 7.30 7.30 7.29 7.28 7.27 7.26 6.06
4.40 4.39 4.38 4.37 4.36 4.35 4.34 4.33 4.32 4.31 4.29
1.20 1.18 0.84 0.82 -0.00
2.00 5.05 3.20 2.00 1.97 6.02 6.05
f1 (ppm)

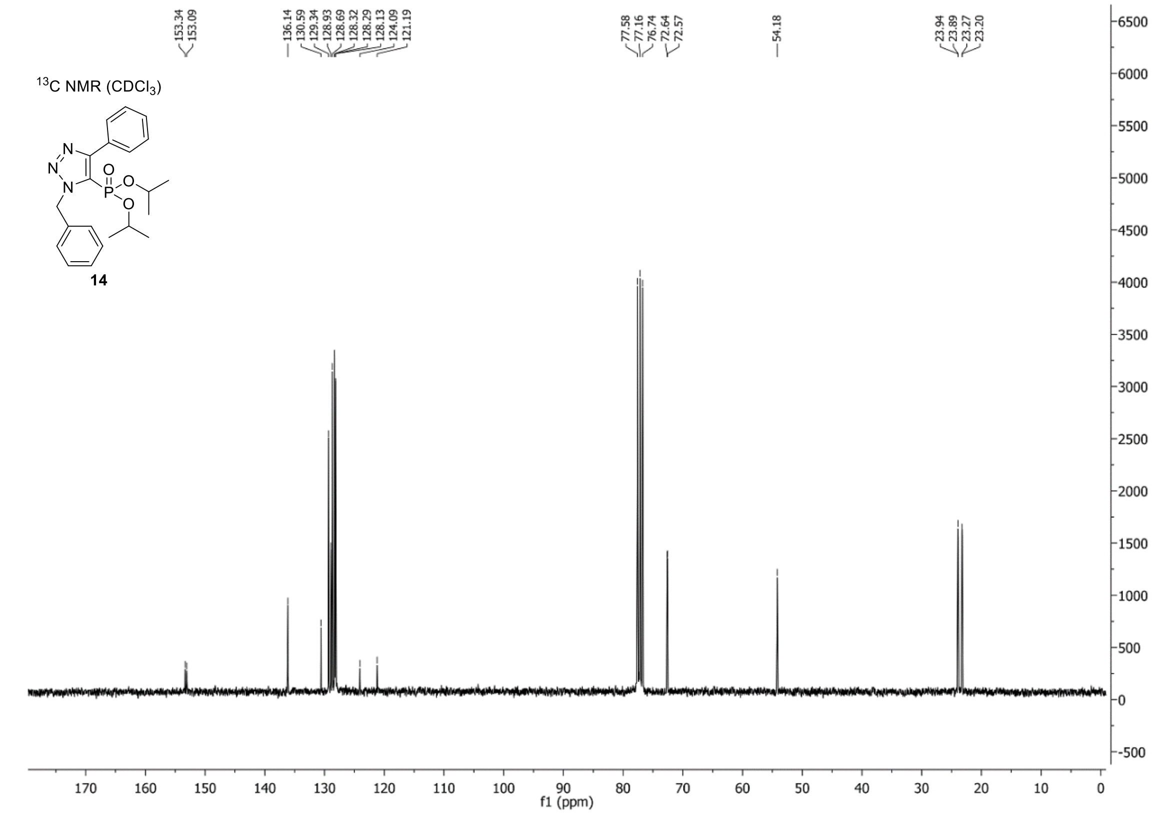

^{13}C NMR ($CDCl_3$)
14
153.34 153.09 136.14 130.59 129.34 128.93 128.69 128.32 128.29 128.13 124.09 121.19
77.58 77.16 76.74 72.64 72.57 54.18
23.94 23.89 23.27 23.20
f1 (ppm)

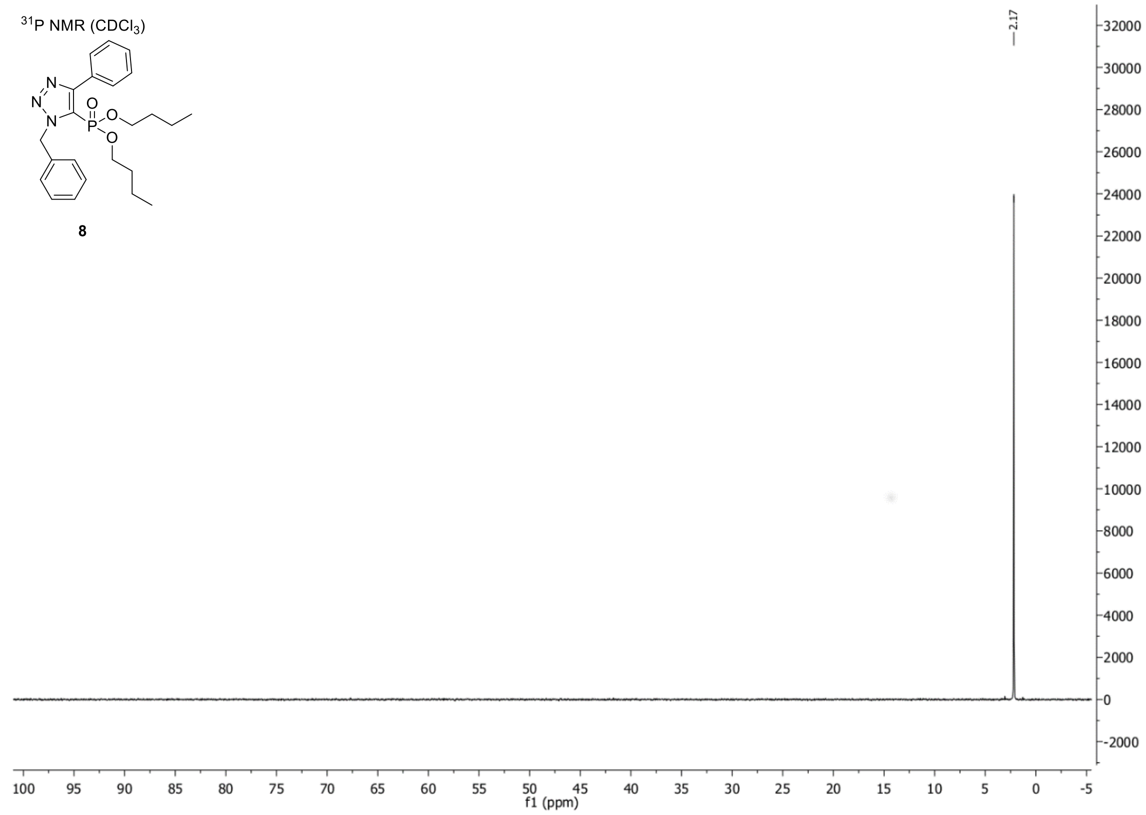

31P NMR (CDCl3)
8
2.17
f1 (ppm)

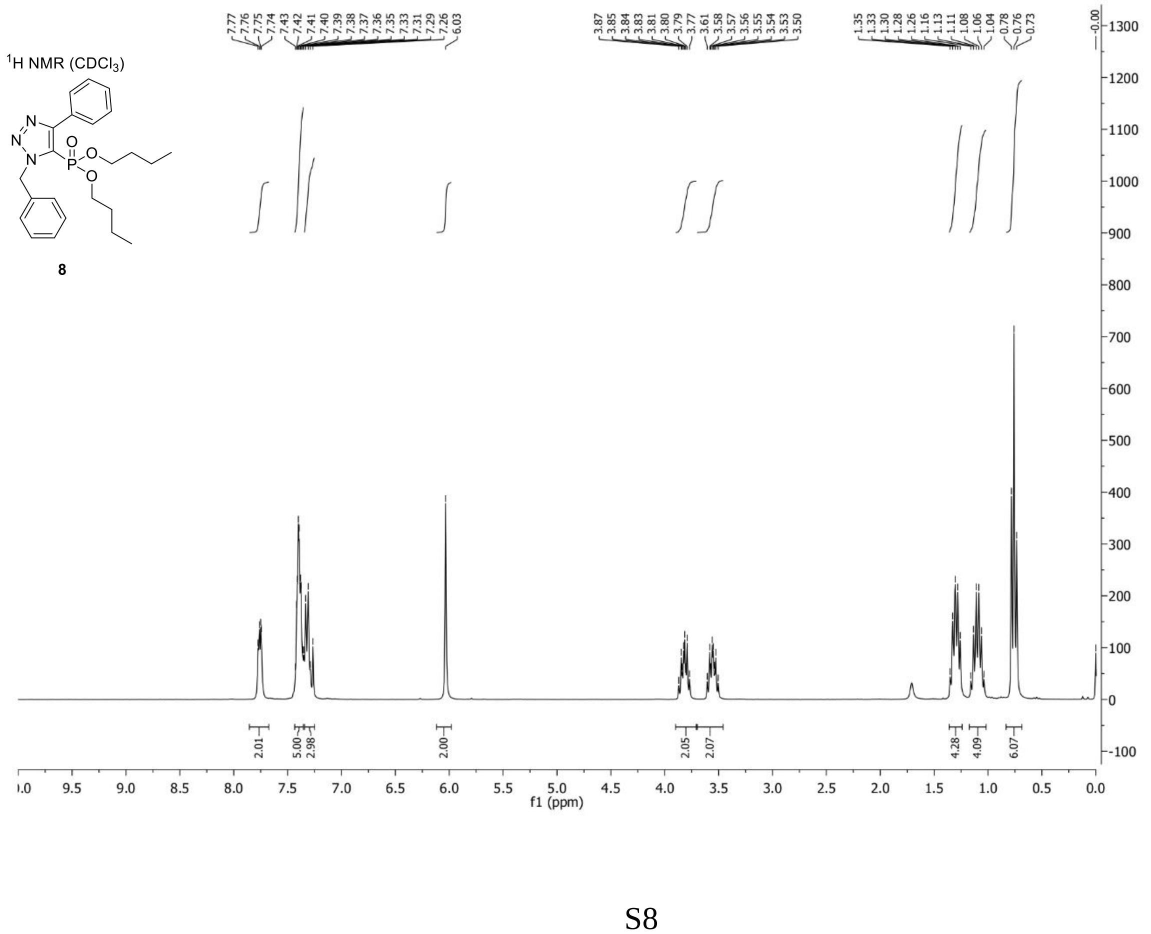

1H NMR (CDCl3)
8
f1 (ppm)

^{13}C NMR ($CDCl_3$)

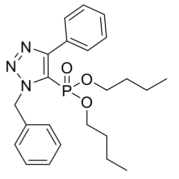

8

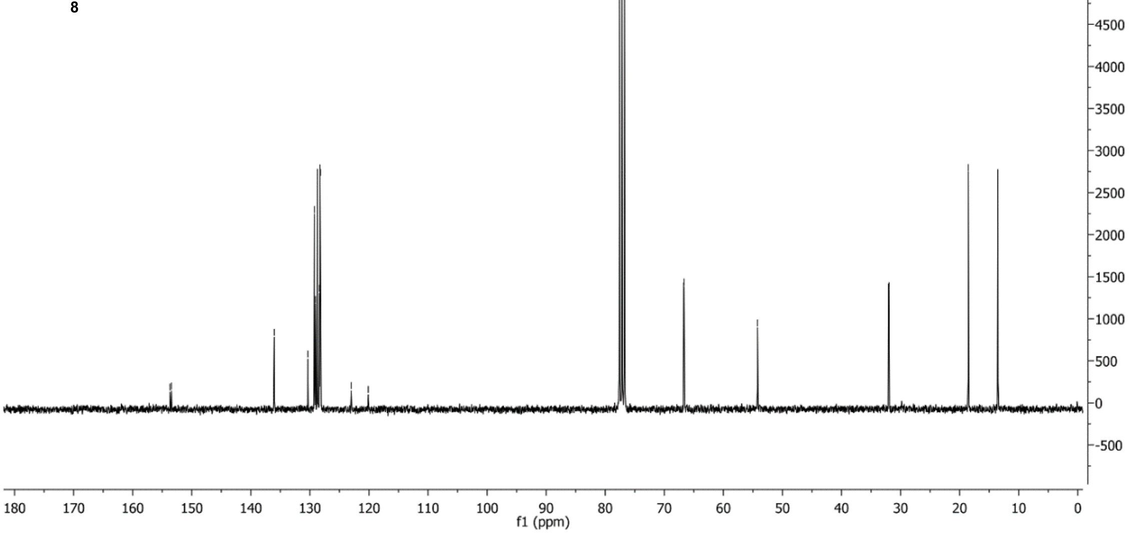

^{31}P NMR ($CDCl_3$)

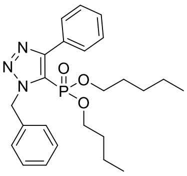

15

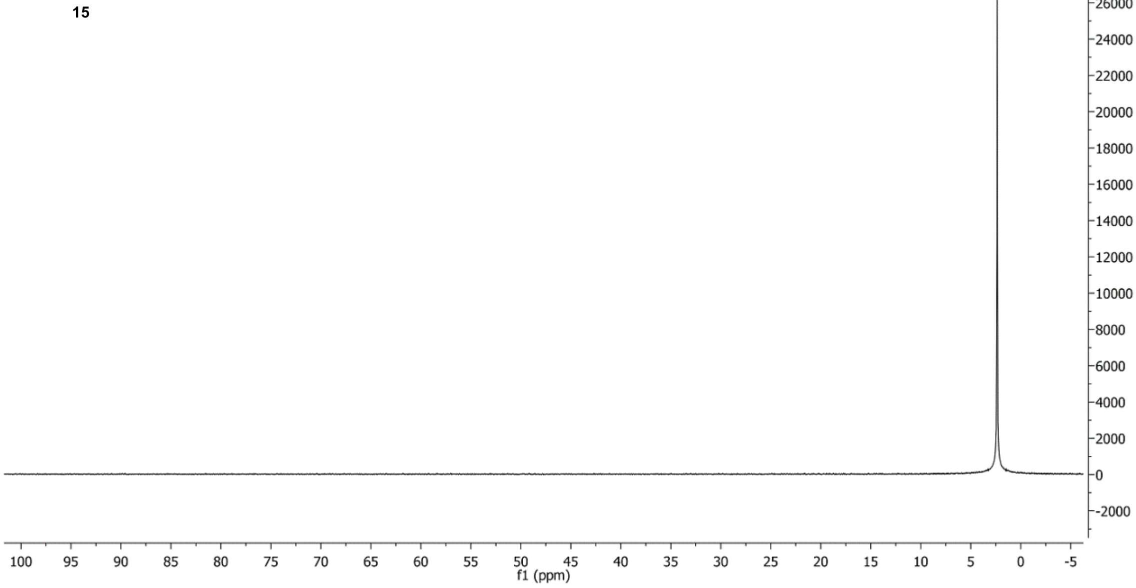

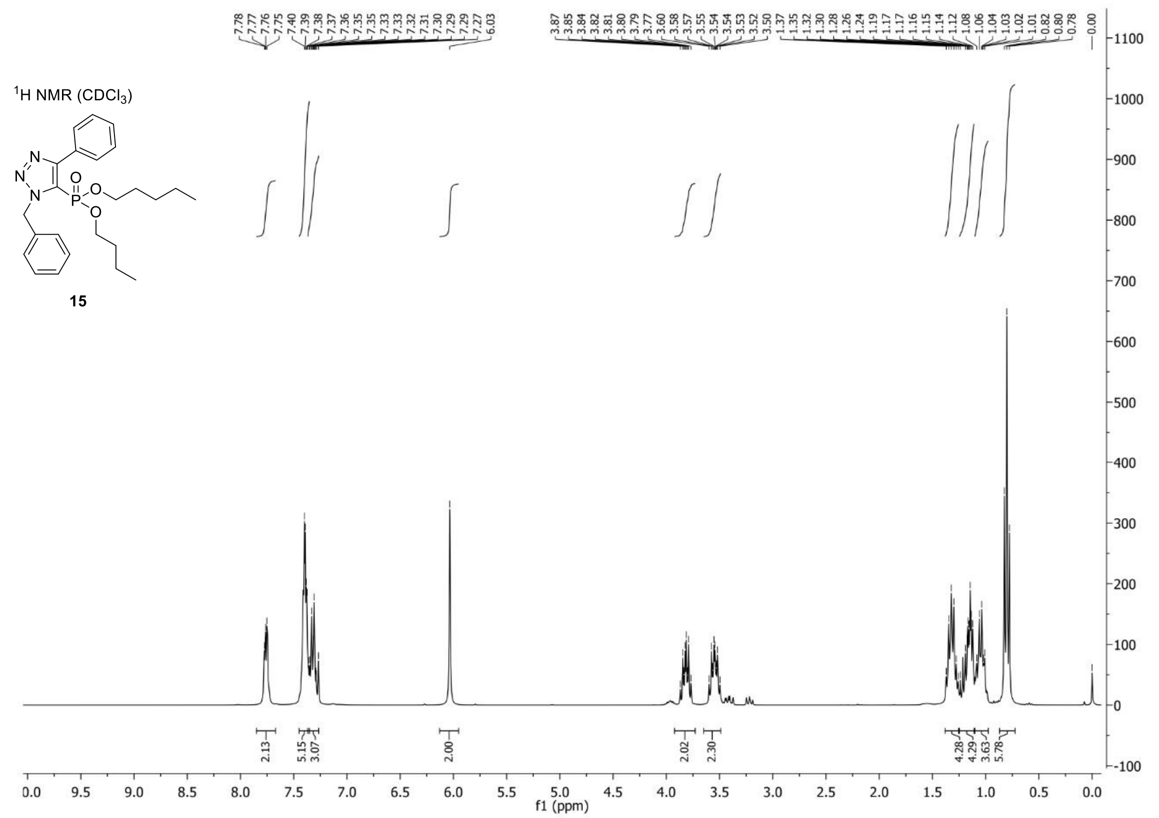
^{1}H NMR ($CDCl_3$)
15
7.78 7.77 7.76 7.75 7.40 7.39 7.38 7.37 7.36 7.35 7.35 7.33 7.32 7.31 7.30 7.29 7.29 7.27 6.03
3.87 3.85 3.84 3.82 3.81 3.80 3.79 3.77 3.60 3.58 3.57 3.55 3.54 3.54 3.53 3.52 3.50
1.37 1.35 1.32 1.30 1.28 1.26 1.24 1.19 1.17 1.17 1.16 1.15 1.14 1.12 1.08 1.06 1.04 1.03 1.02 1.01 0.82 0.80 0.78
0.00
2.13 5.15 3.07 2.00 2.02 2.30 4.28 4.29 3.63 5.78
f1 (ppm)

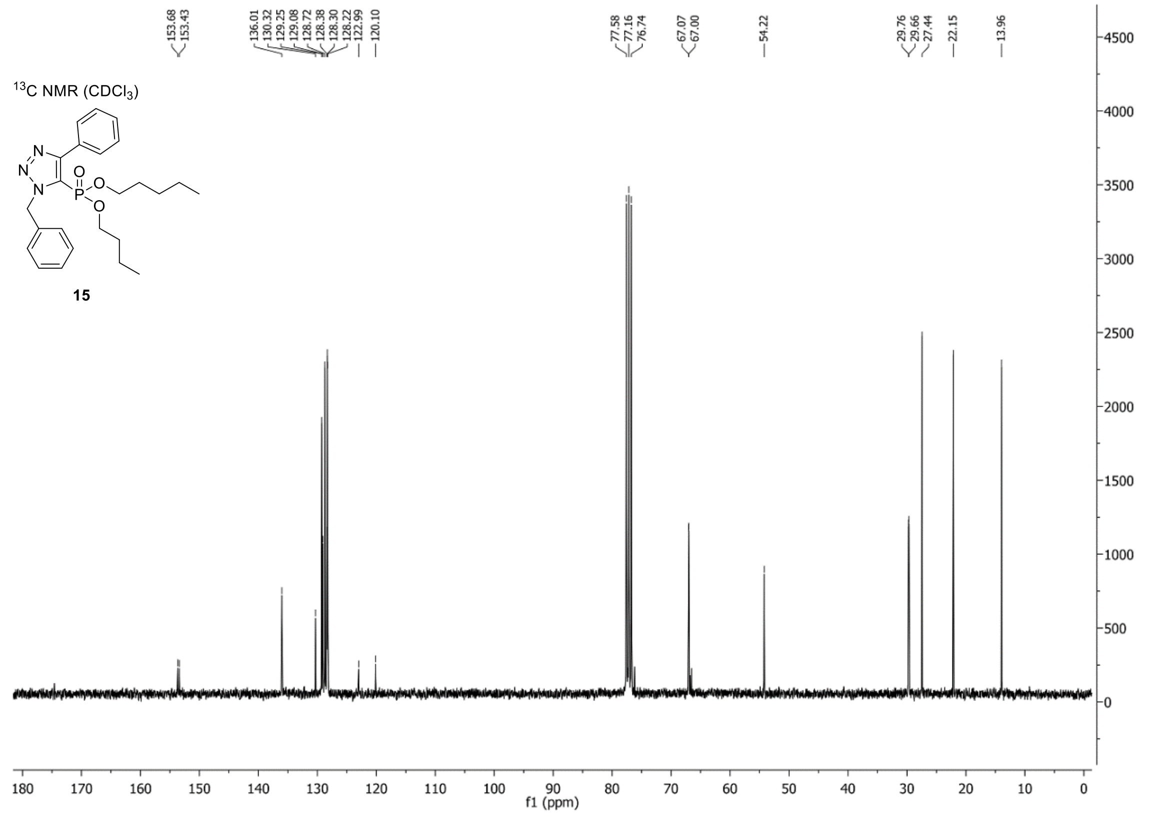
^{13}C NMR ($CDCl_3$)
15
153.68 153.43
136.01 130.32 129.25 129.08 128.72 128.38 128.30 128.22 122.99 120.10
77.58 77.16 76.74
67.07 67.00
54.22
29.76 29.66 27.44 22.15 13.96
f1 (ppm)

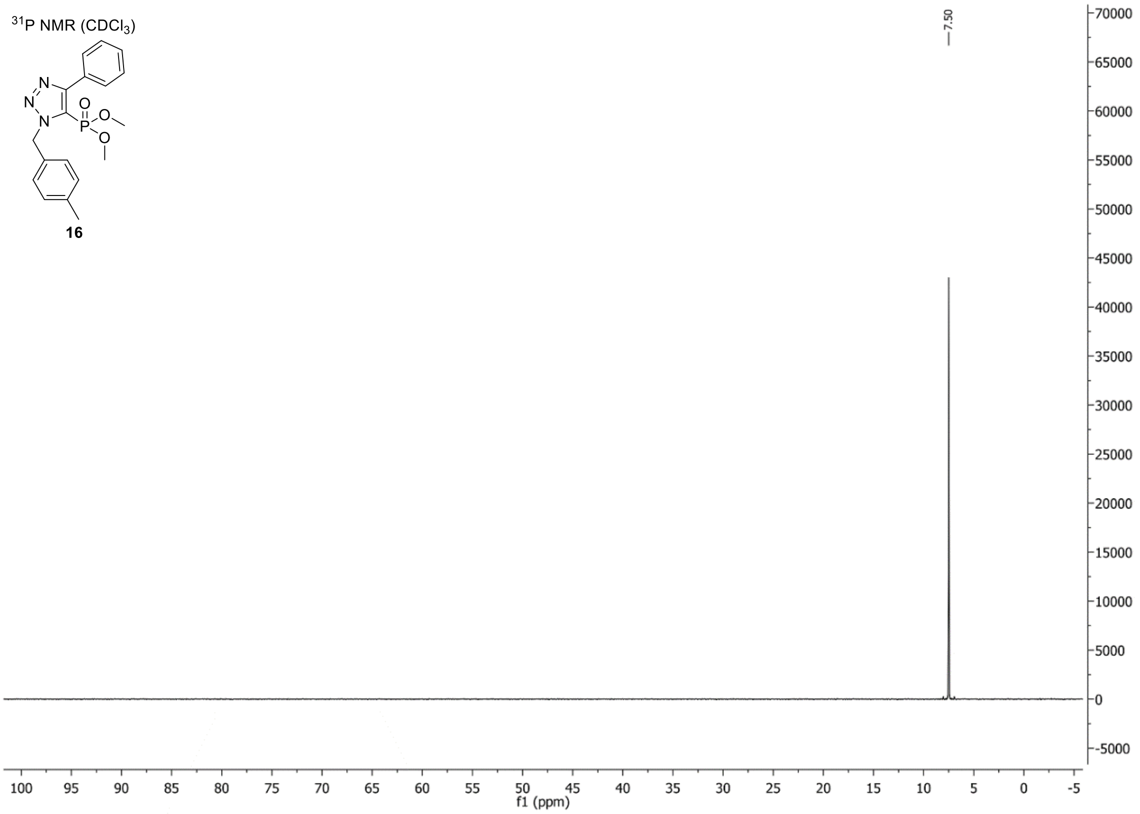
31P NMR (CDCl3)
16
7.50
f1 (ppm)

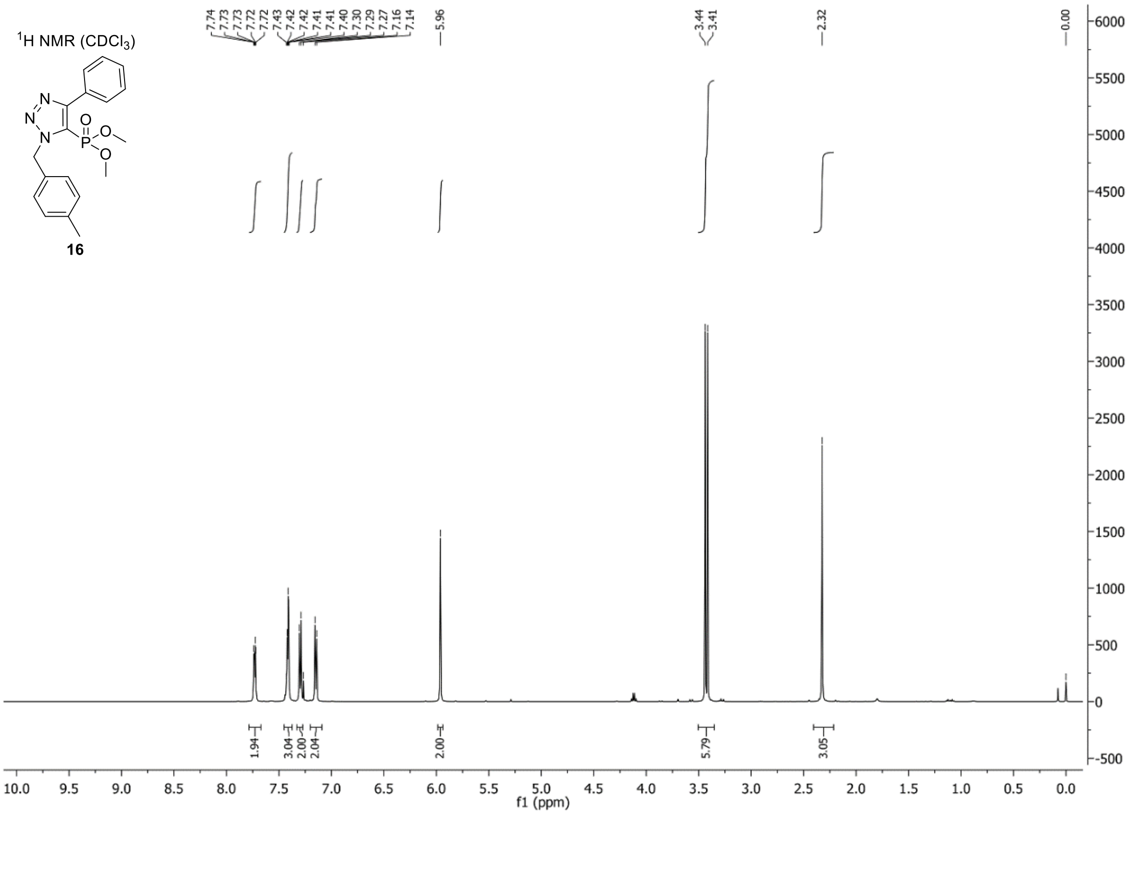
1H NMR (CDCl3)
16
7.74 7.73 7.73 7.72 7.72 7.43 7.42 7.42 7.41 7.41 7.40 7.30 7.29 7.27 7.16 7.14
5.96
3.44 3.41
2.32
0.00
1.94 3.04 2.00 2.04 2.00 5.79 3.05
f1 (ppm)

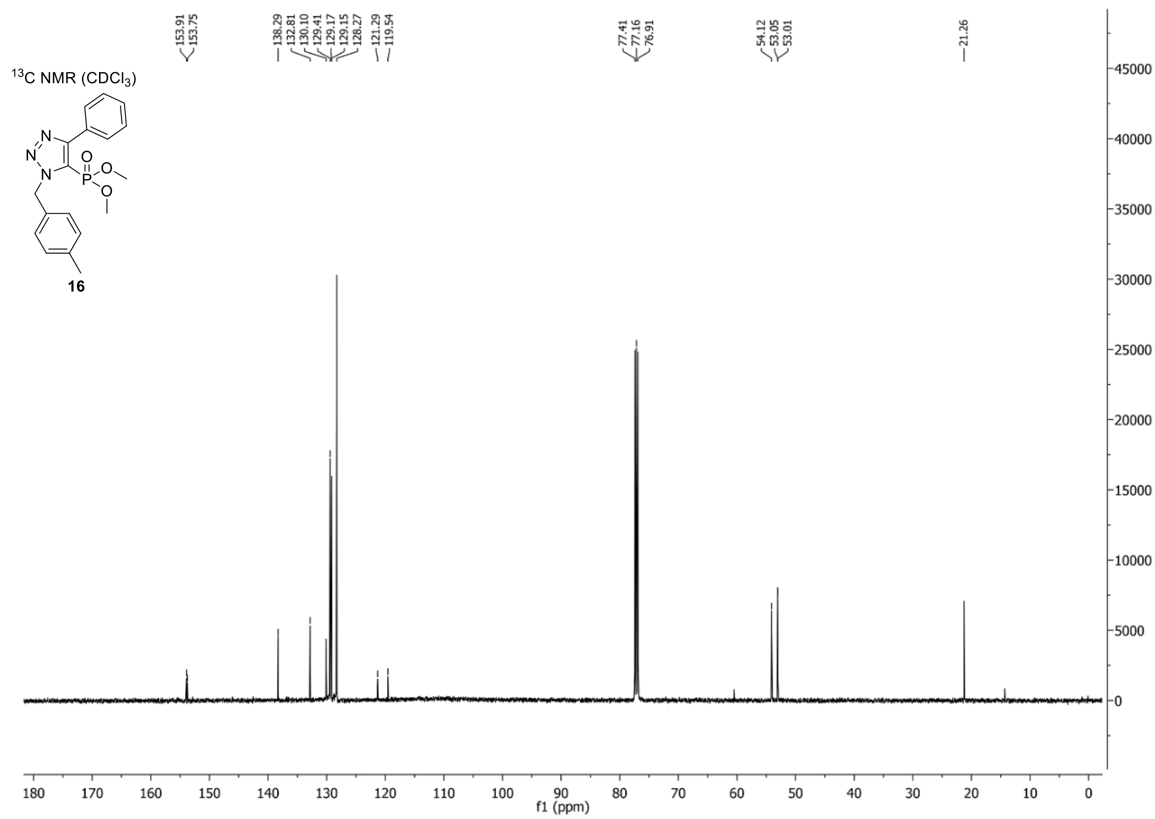

13C NMR (CDCl3)
16
153.91
153.75
138.29
132.81
130.10
129.41
129.17
129.15
128.27
121.29
119.54
77.41
77.16
76.91
54.12
53.05
53.01
21.26
f1 (ppm)

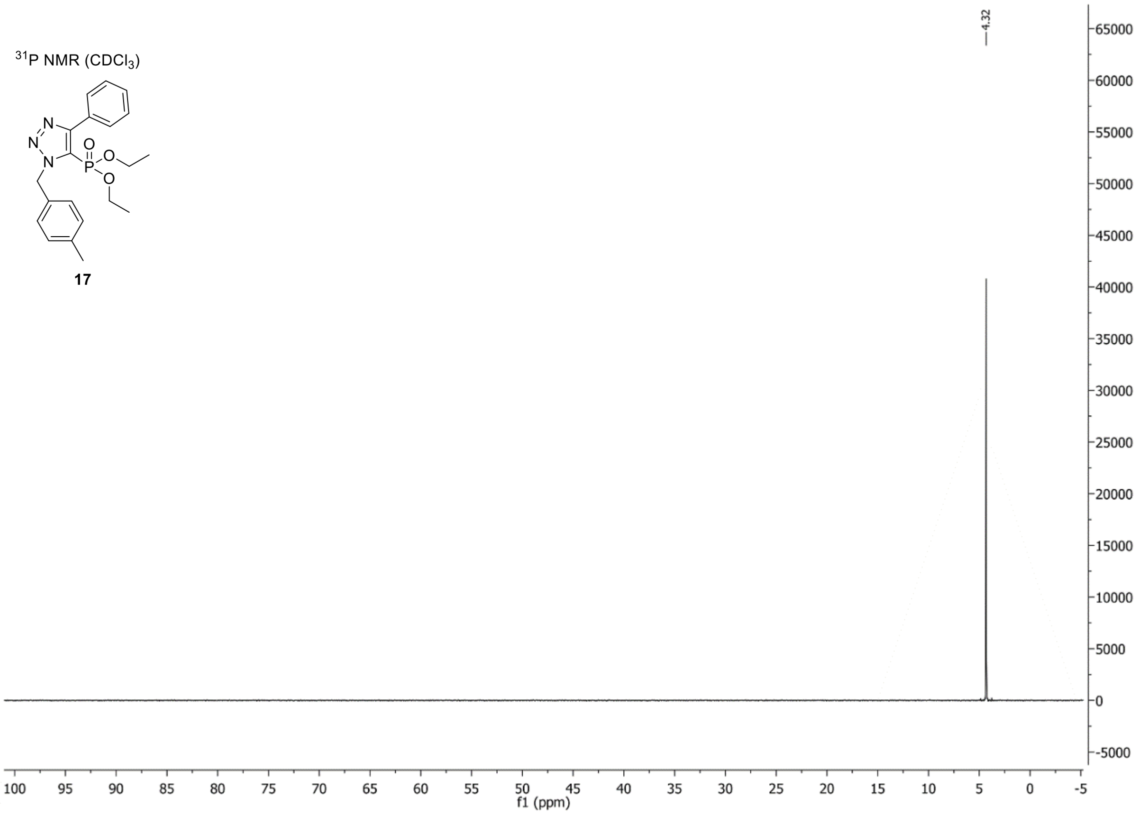

31P NMR (CDCl3)
17
4.32
f1 (ppm)

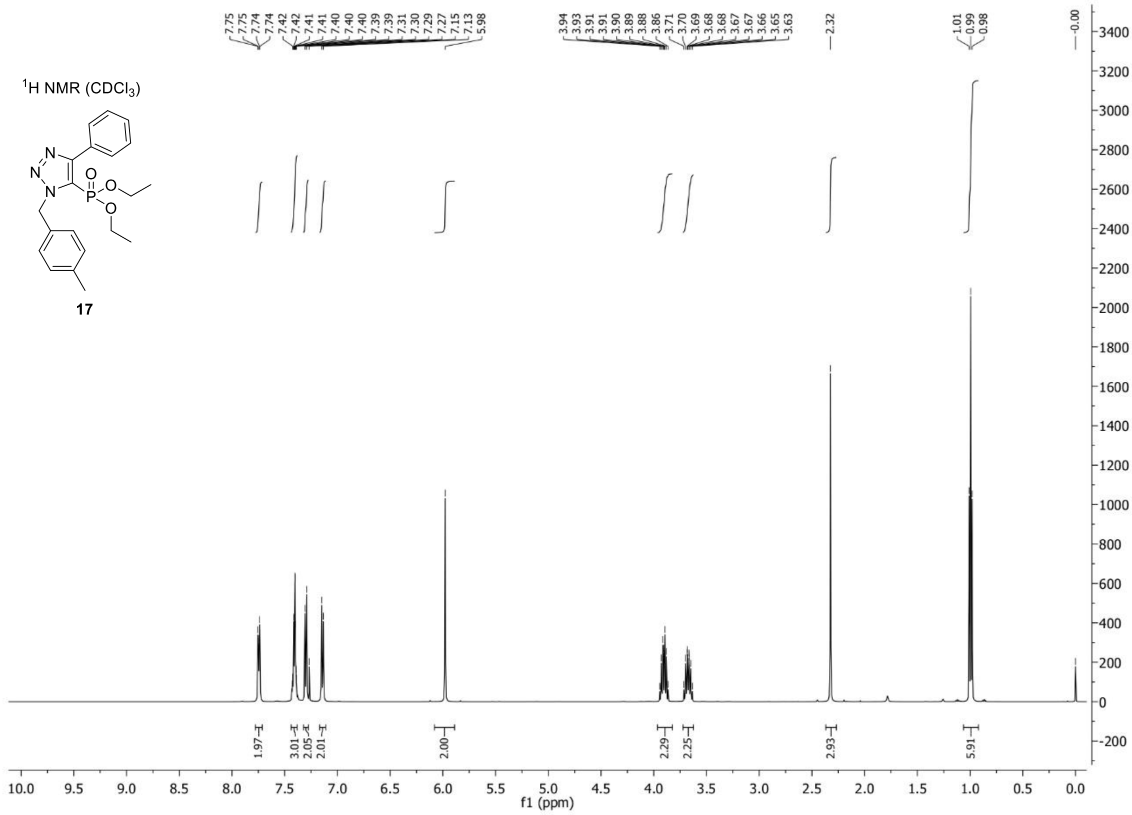

^{1}H NMR (CDCl$_3$)
17
f1 (ppm)

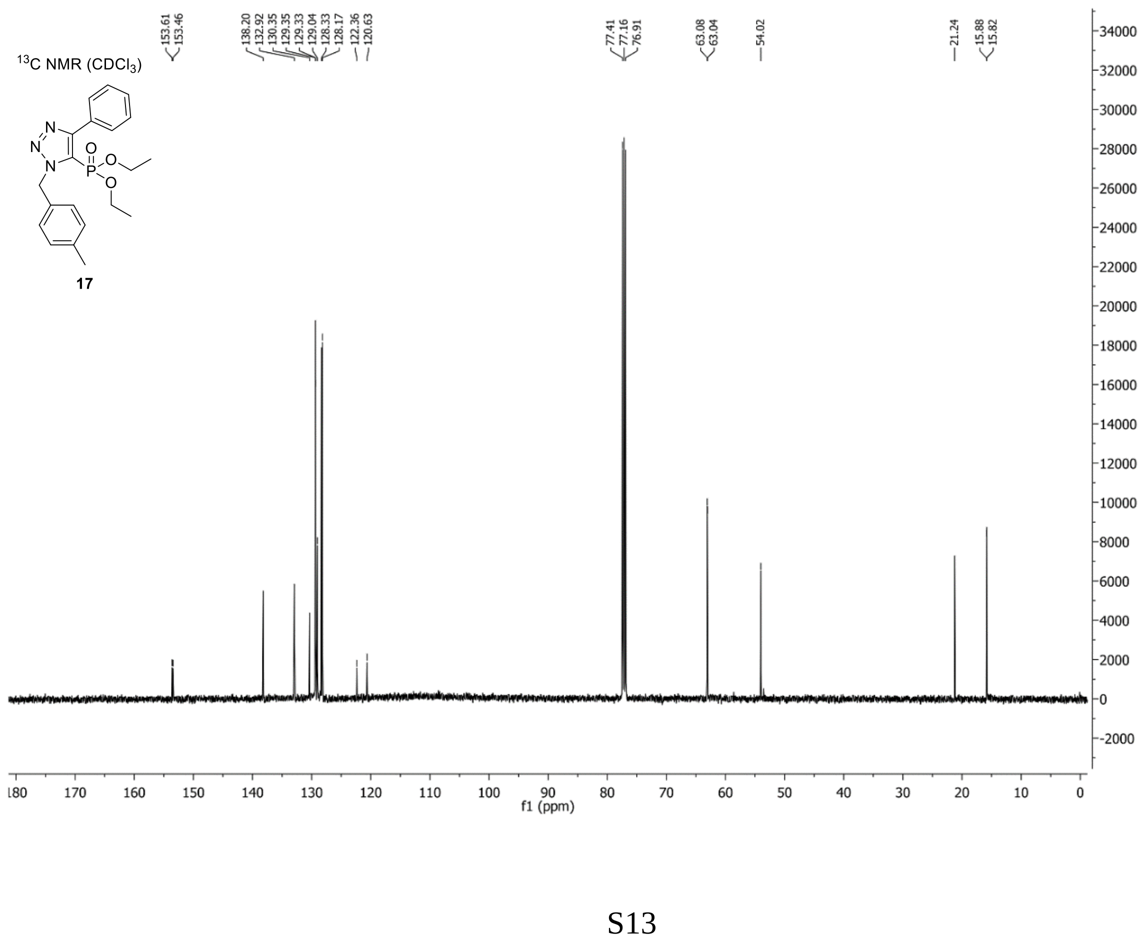

^{13}C NMR (CDCl$_3$)
17
f1 (ppm)

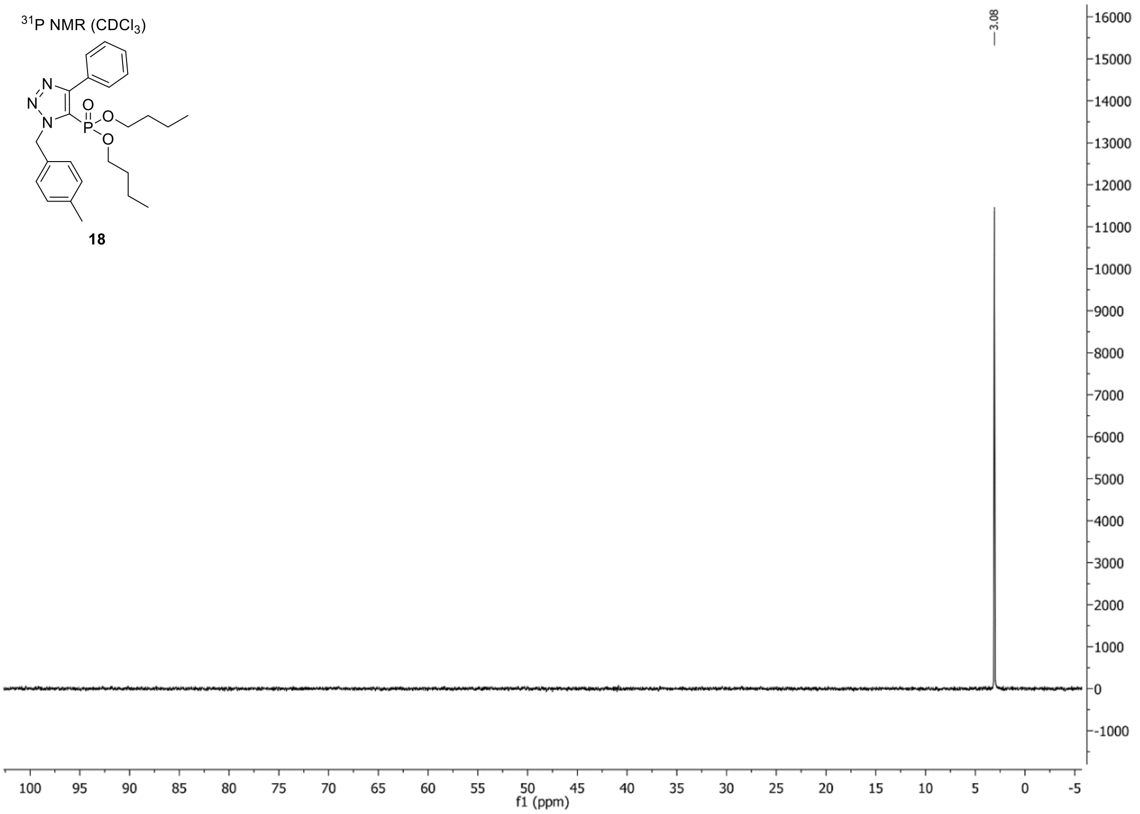

31P NMR (CDCl3)
18
3.08
f1 (ppm)

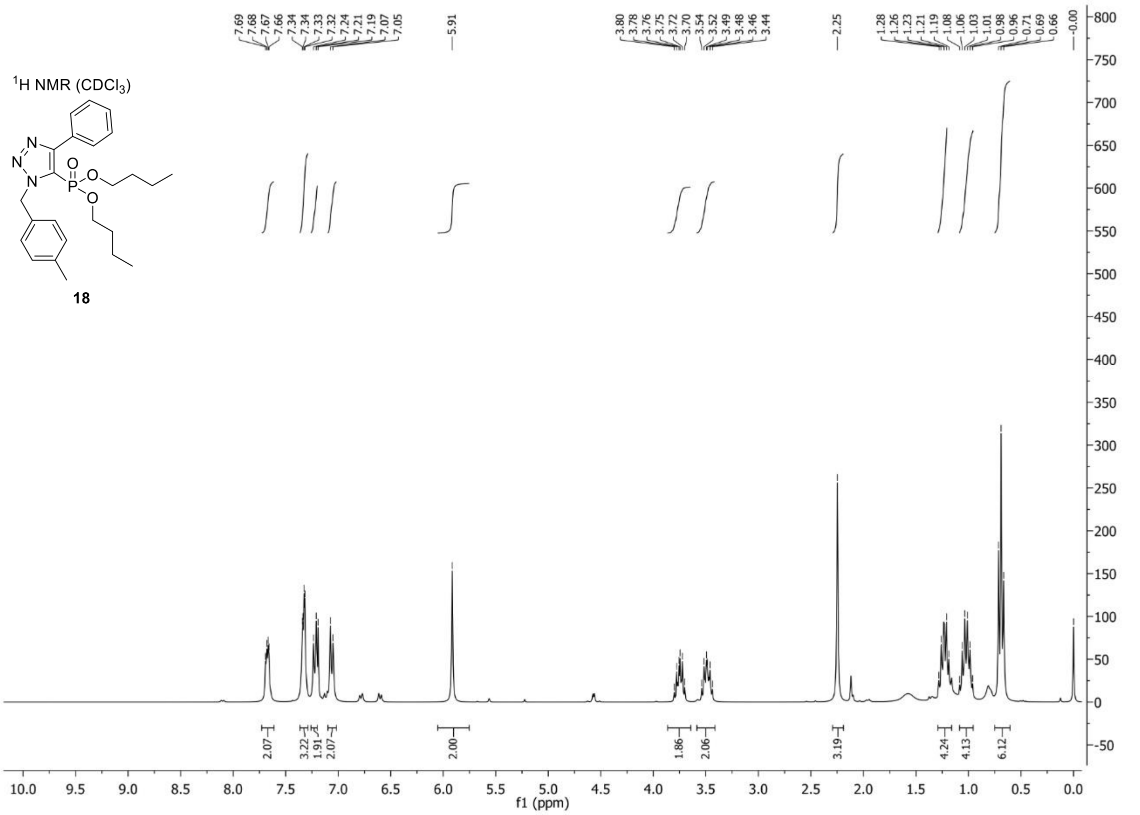

1H NMR (CDCl3)
18
f1 (ppm)

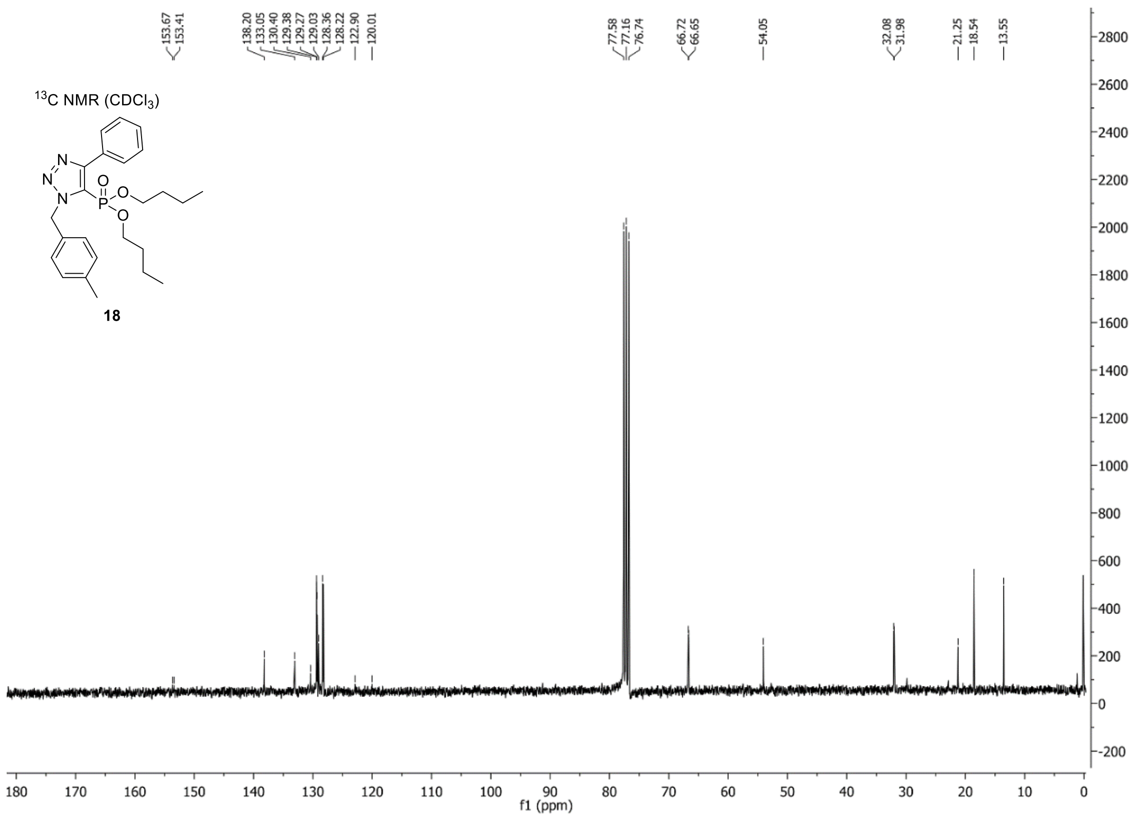
13C NMR (CDCl3)
18
153.67
153.41
138.20
133.05
130.40
129.38
129.27
129.03
128.36
128.22
122.90
120.01
77.58
77.16
76.74
66.72
66.65
54.05
32.08
31.98
21.25
18.54
13.55
f1 (ppm)

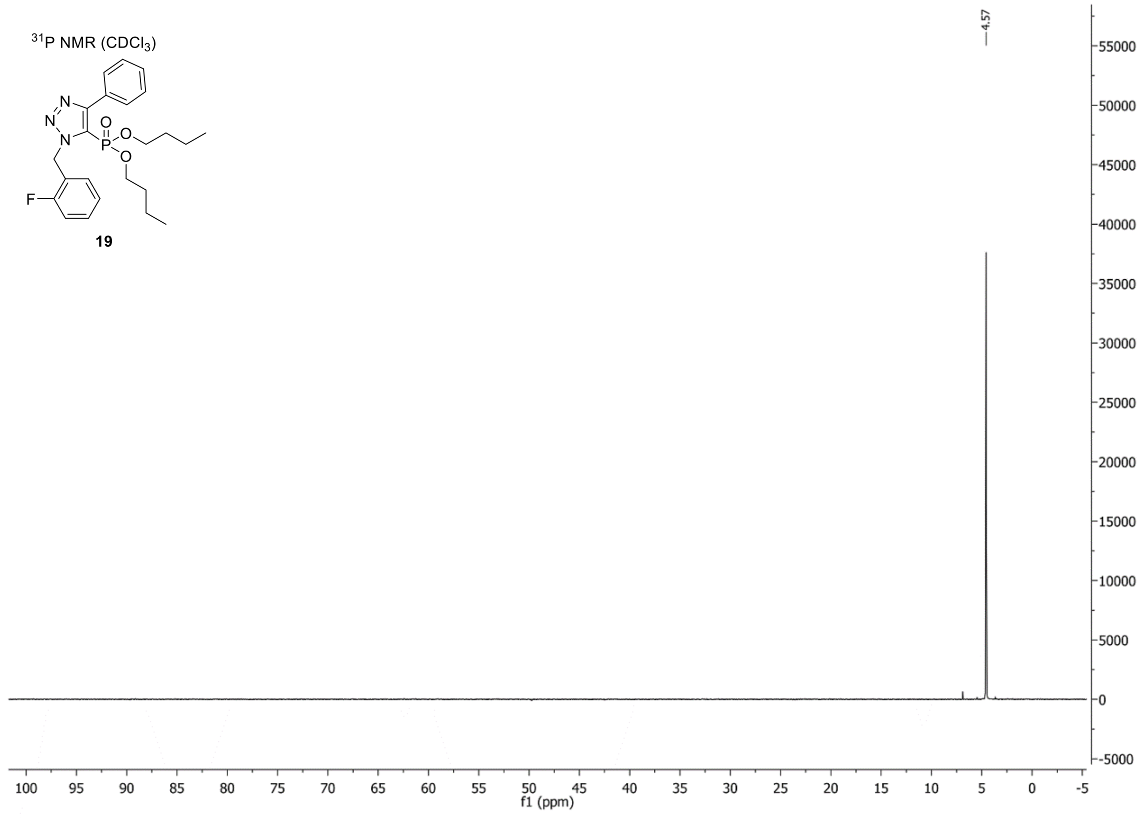
31P NMR (CDCl3)
19
4.57
f1 (ppm)

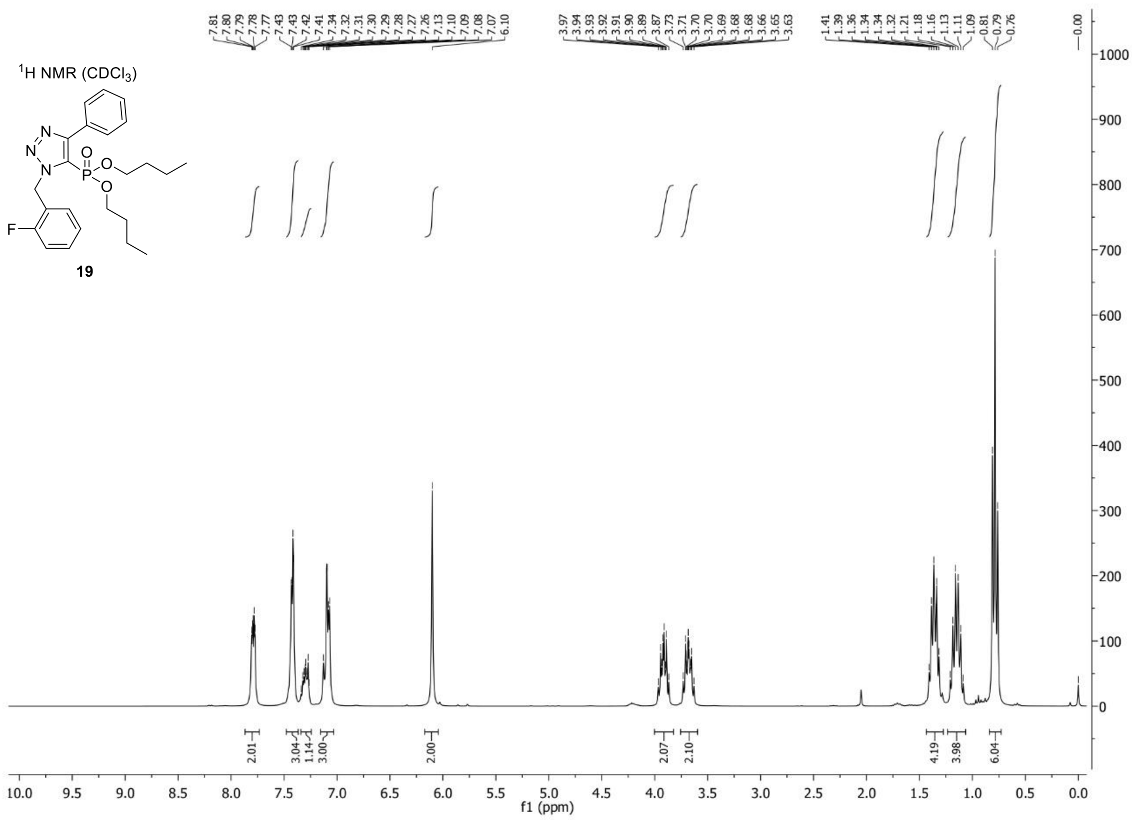
1H NMR (CDCl3)
19
7.81 7.80 7.79 7.78 7.77 7.43 7.43 7.42 7.41 7.34 7.32 7.31 7.30 7.29 7.28 7.27 7.26 7.13 7.10 7.09 7.08 7.07 6.10
3.97 3.94 3.93 3.92 3.91 3.90 3.89 3.87 3.73 3.71 3.70 3.70 3.69 3.68 3.68 3.66 3.65 3.63
1.41 1.39 1.36 1.34 1.34 1.32 1.21 1.18 1.16 1.13 1.11 1.09 0.81 0.79 0.76
0.00
2.01 3.04 1.14 3.00 2.00 2.07 2.10 4.19 3.98 6.04
f1 (ppm)

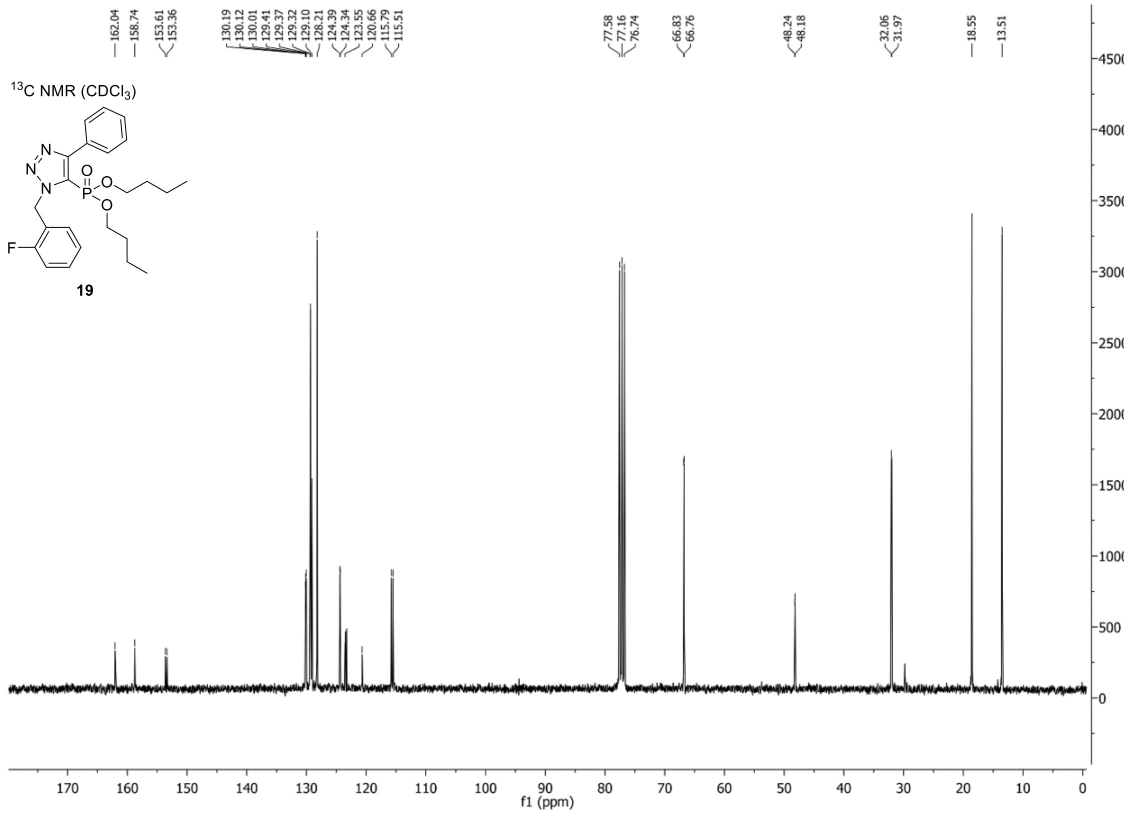
13C NMR (CDCl3)
19
162.04 158.74 153.61 153.36
130.19 130.12 130.01 129.41 129.37 129.32 129.10 128.21 124.39 124.34 123.55 120.66 115.79 115.51
77.58 77.16 76.74 66.83 66.76
48.24 48.18
32.06 31.97
18.55
13.51
f1 (ppm)

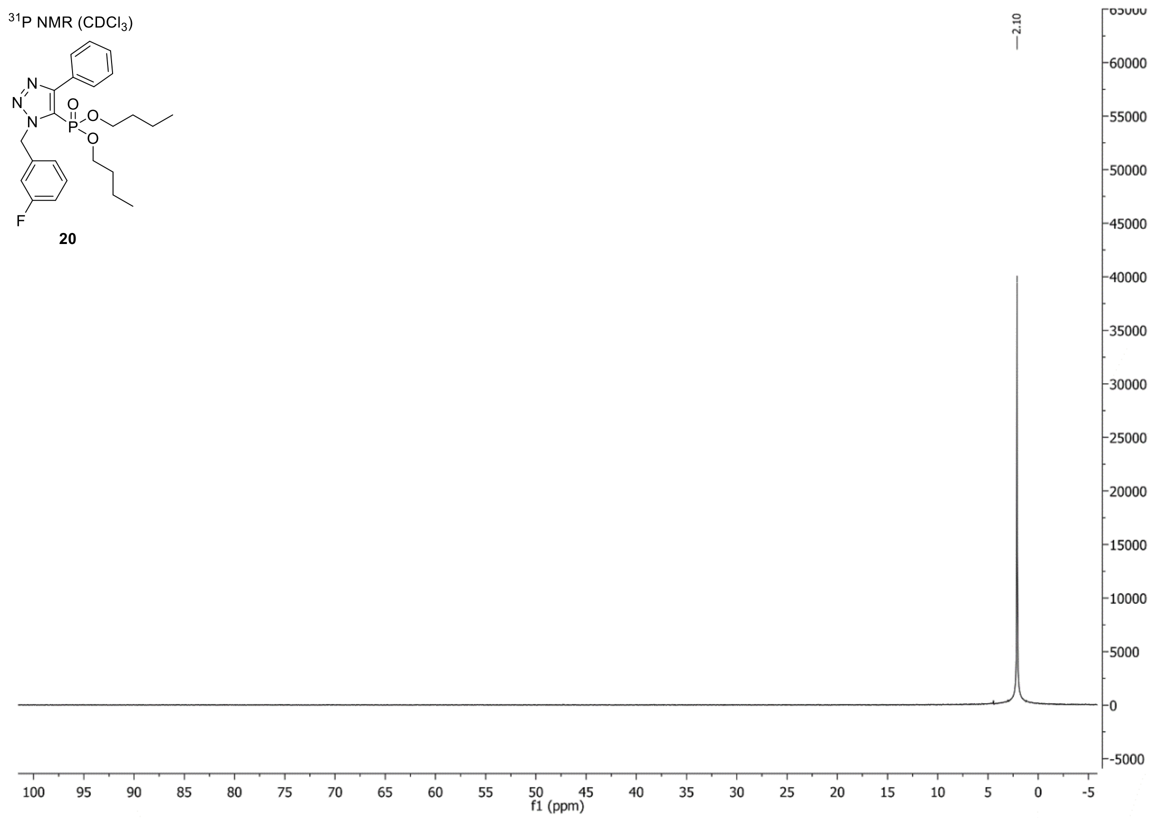

^{31}P NMR (CDCl$_3$)
20
2.10
f1 (ppm)

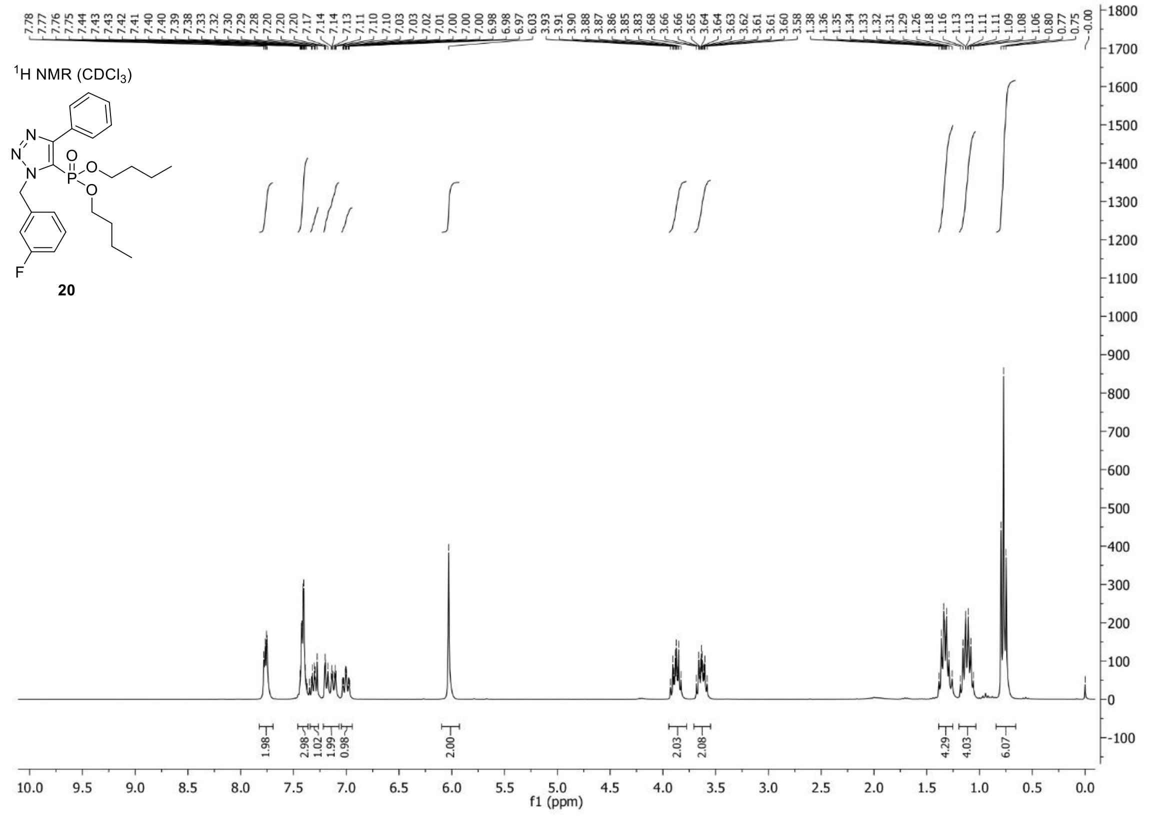

^{1}H NMR (CDCl$_3$)
20
1.98
2.98
1.02
1.99
0.98
2.00
2.03
2.08
4.29
4.03
6.07
f1 (ppm)

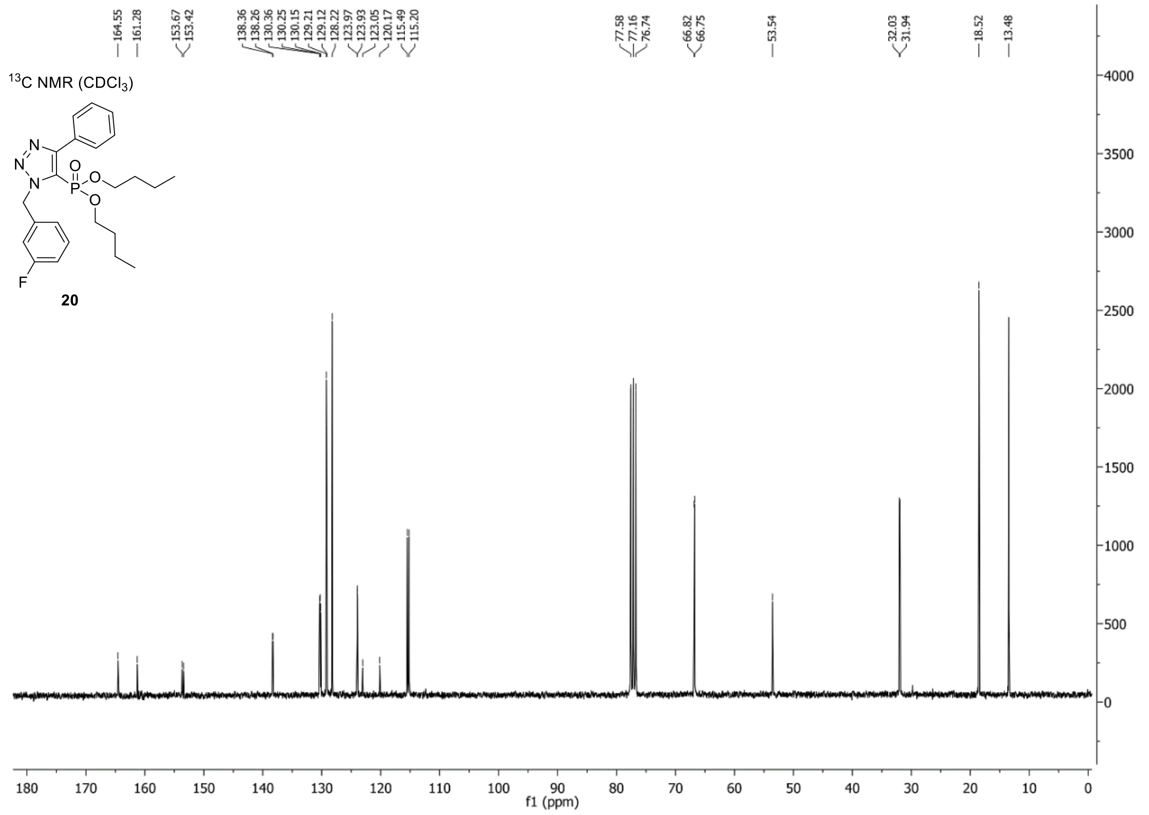
13C NMR (CDCl3)
20
164.55
161.28
153.67
153.42
138.36
138.26
130.36
130.25
130.15
129.21
129.12
128.22
123.97
123.93
123.05
120.17
115.49
115.20
77.58
77.16
76.74
66.82
66.75
53.54
32.03
31.94
18.52
13.48
f1 (ppm)

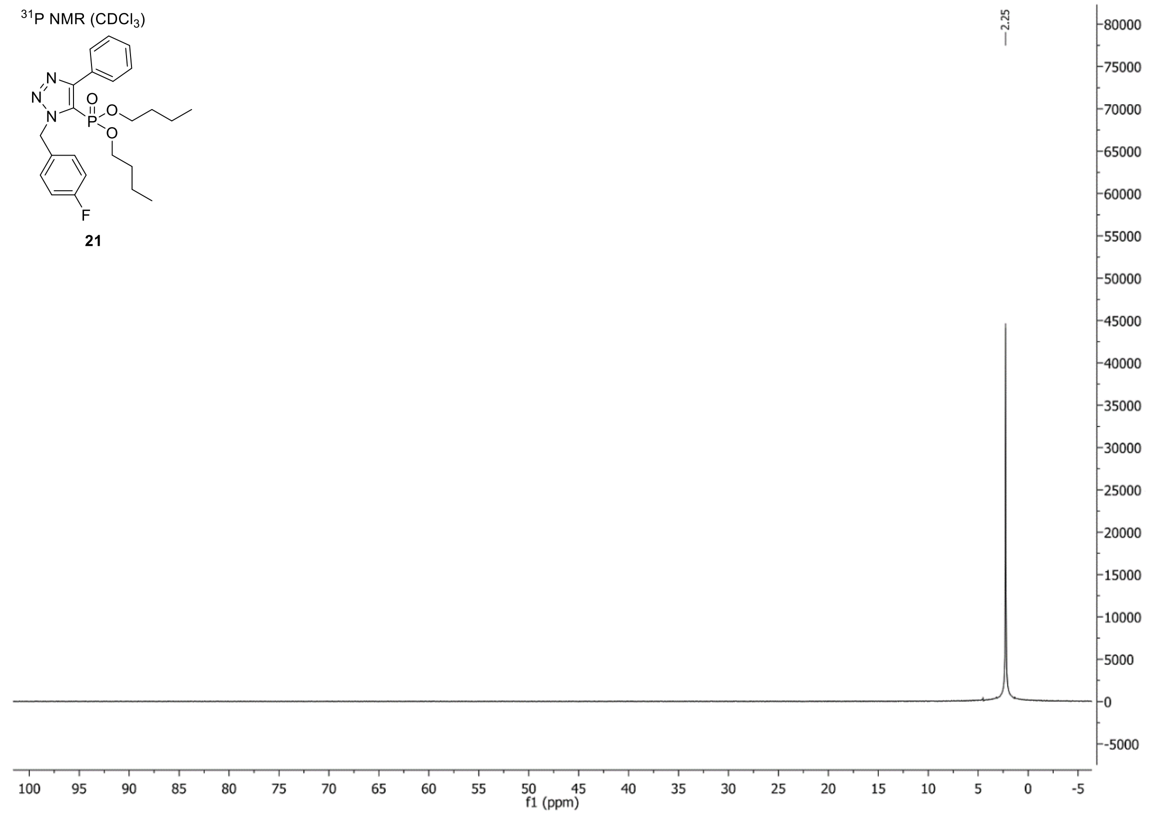
31P NMR (CDCl3)
21
2.25
f1 (ppm)

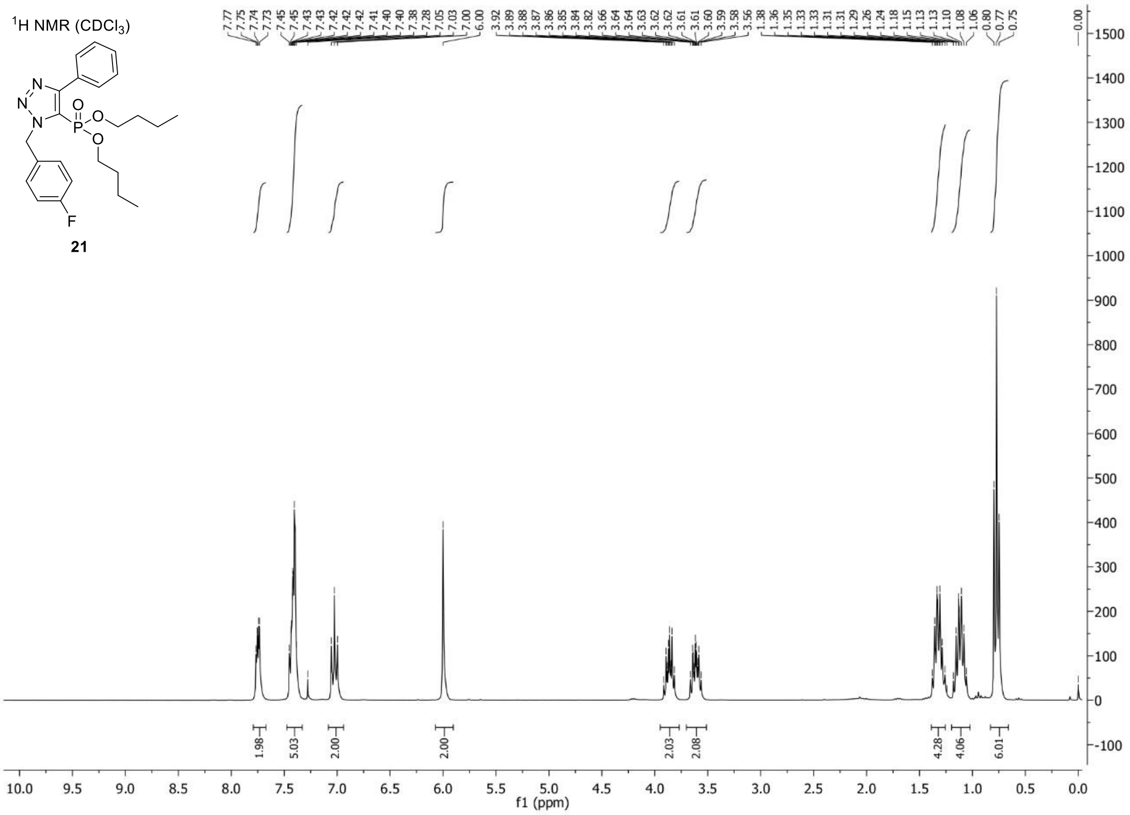
1H NMR (CDCl3)
21
7.77 7.75 7.74 7.73 7.45 7.45 7.43 7.43 7.42 7.42 7.41 7.40 7.40 7.38 7.28 7.05 7.03 7.00 6.00
3.92 3.89 3.88 3.87 3.86 3.85 3.84 3.82 3.66 3.64 3.64 3.63 3.62 3.62 3.61 3.61 3.60 3.59 3.58 3.56
1.38 1.36 1.35 1.33 1.33 1.31 1.31 1.29 1.26 1.24 1.18 1.15 1.13 1.13 1.10 1.08 1.06 0.80 0.77 0.75
0.00
1.98 5.03 2.00 2.00 2.03 2.08 4.28 4.06 6.01
f1 (ppm)

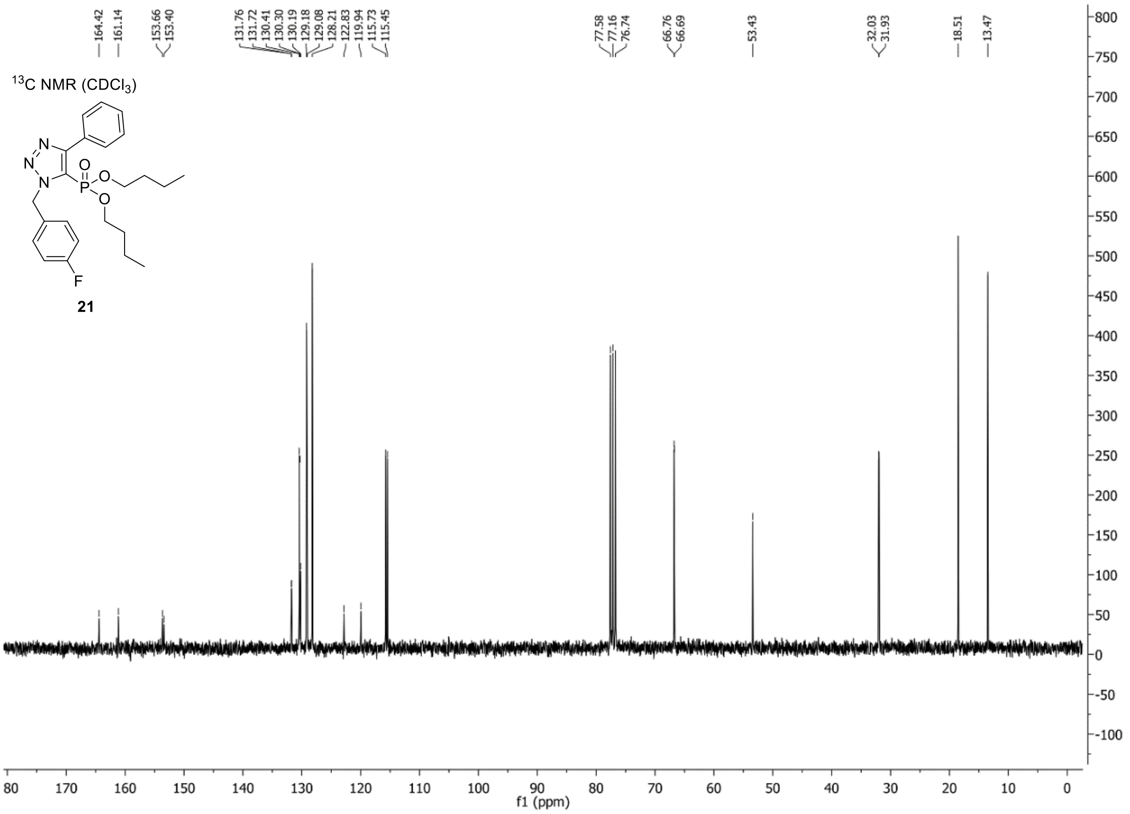
13C NMR (CDCl3)
21
164.42 161.14 153.66 153.40
131.76 131.72 130.41 130.30 130.19 129.18 129.08 128.21 122.83 119.94 115.73 115.45
77.58 77.16 76.74 66.76 66.69 53.43
32.03 31.93 18.51 13.47
f1 (ppm)

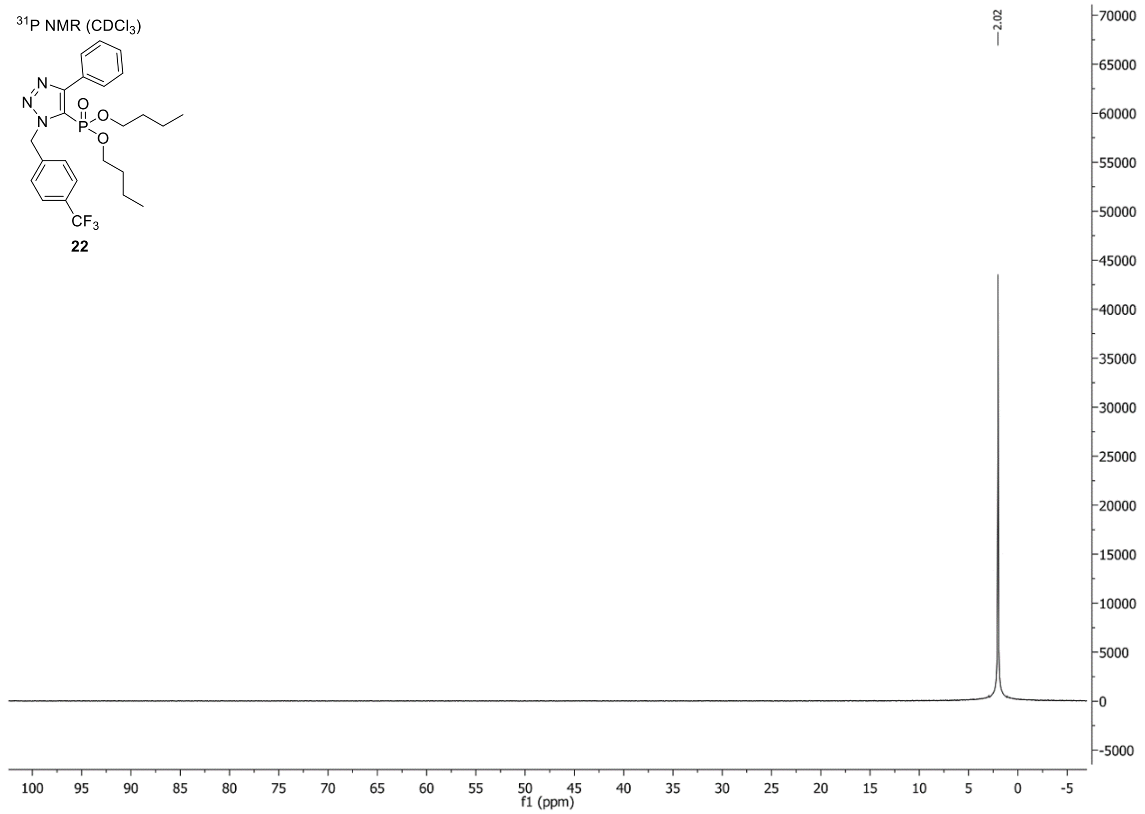

^{31}P NMR (CDCl$_3$)
22
2.02
f1 (ppm)

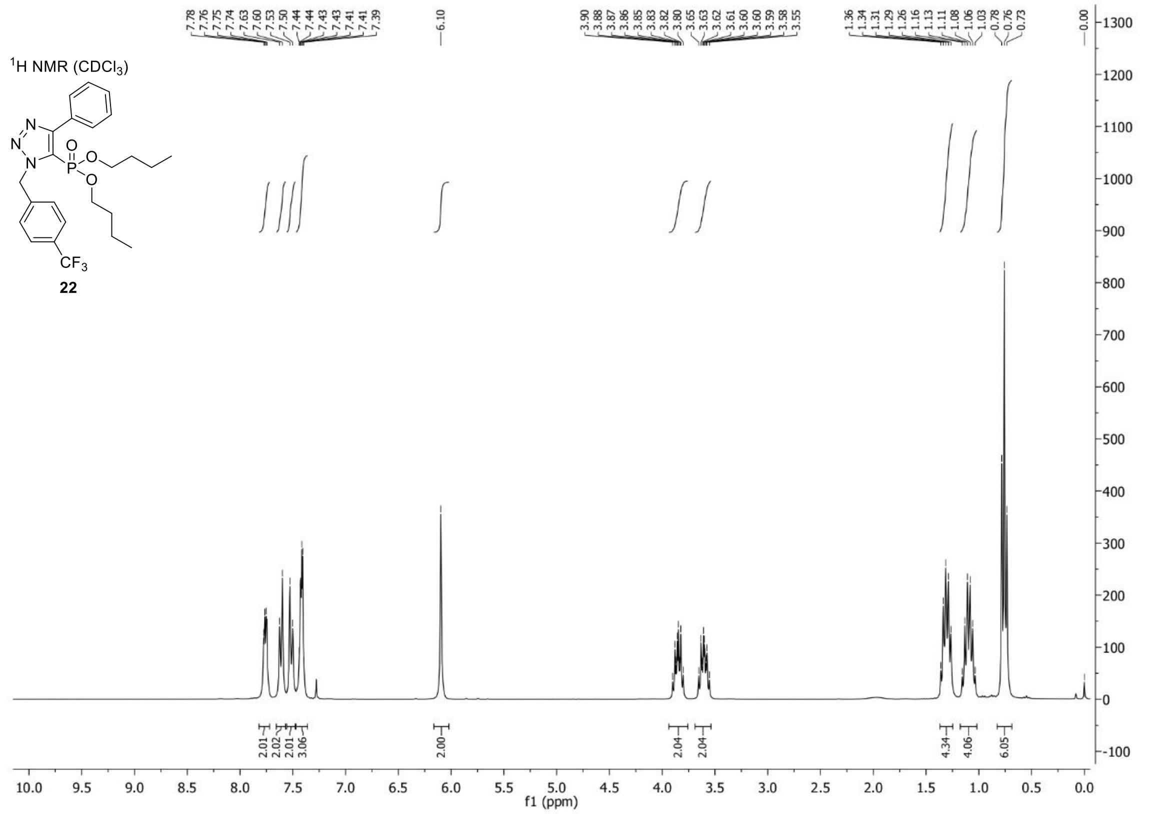

^{1}H NMR (CDCl$_3$)
22
7.78 7.76 7.75 7.74 7.63 7.60 7.53 7.50 7.44 7.44 7.43 7.43 7.41 7.39
6.10
3.90 3.88 3.87 3.86 3.85 3.83 3.82 3.80 3.65 3.63 3.62 3.61 3.60 3.60 3.59 3.58 3.55
1.36 1.34 1.31 1.29 1.26 1.16 1.13 1.11 1.08 1.06 1.03 0.78 0.76 0.73
0.00
2.01 2.02 2.01 3.06 2.00 2.04 2.04 4.34 4.06 6.05
f1 (ppm)

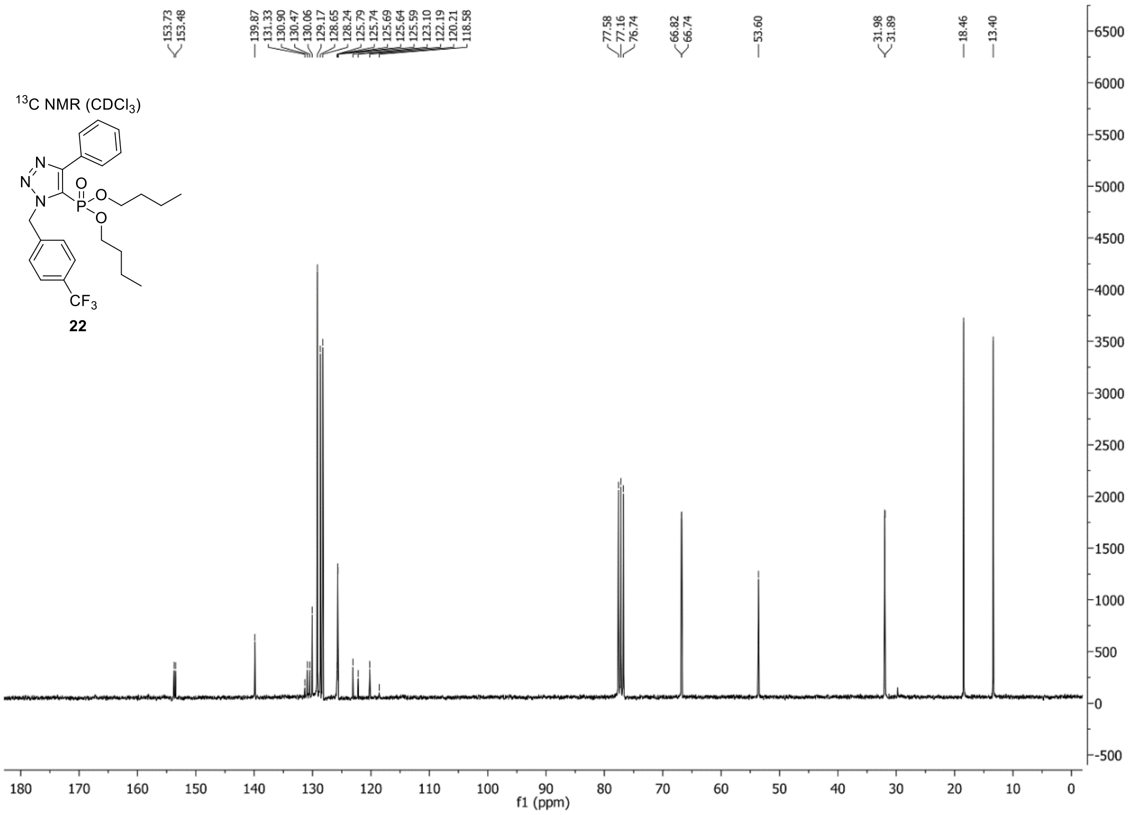

13C NMR (CDCl3)
22
153.73
153.48
139.87
131.33
130.90
130.47
130.06
129.17
128.65
128.24
125.79
125.74
125.69
125.64
125.59
123.10
122.19
120.21
118.58
77.58
77.16
76.74
66.82
66.74
53.60
31.98
31.89
18.46
13.40
f1 (ppm)

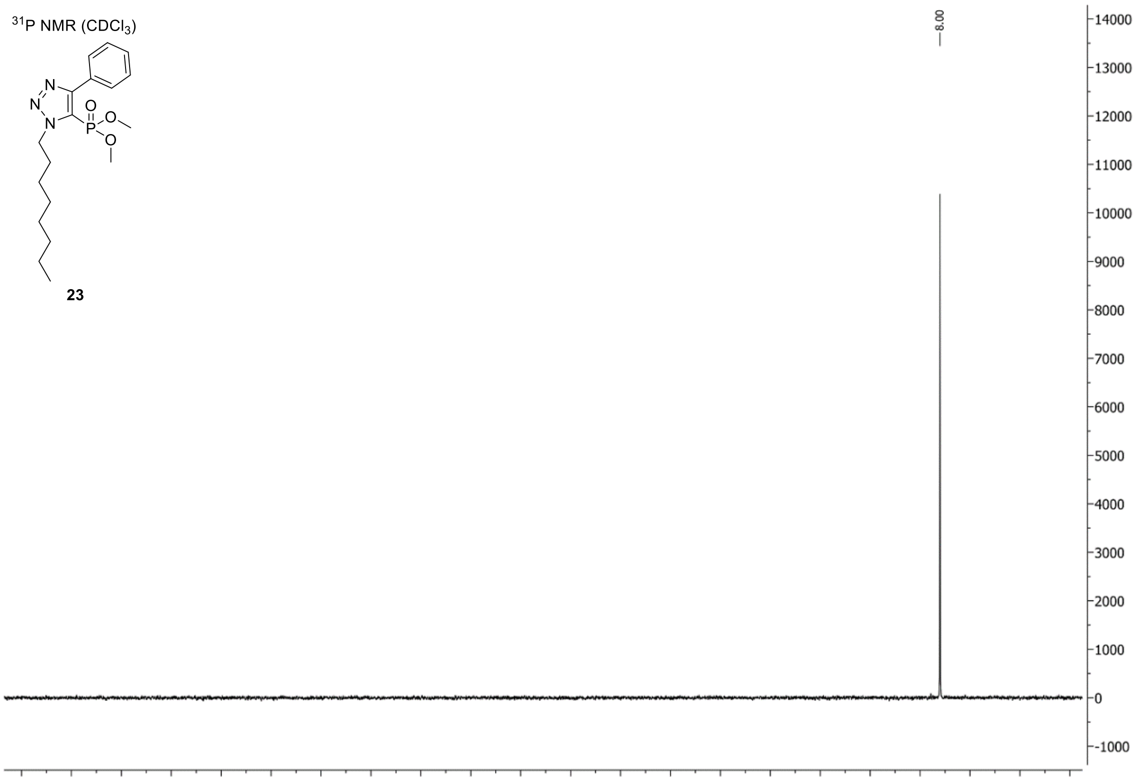

31P NMR (CDCl3)
23
8.00
f1 (ppm)

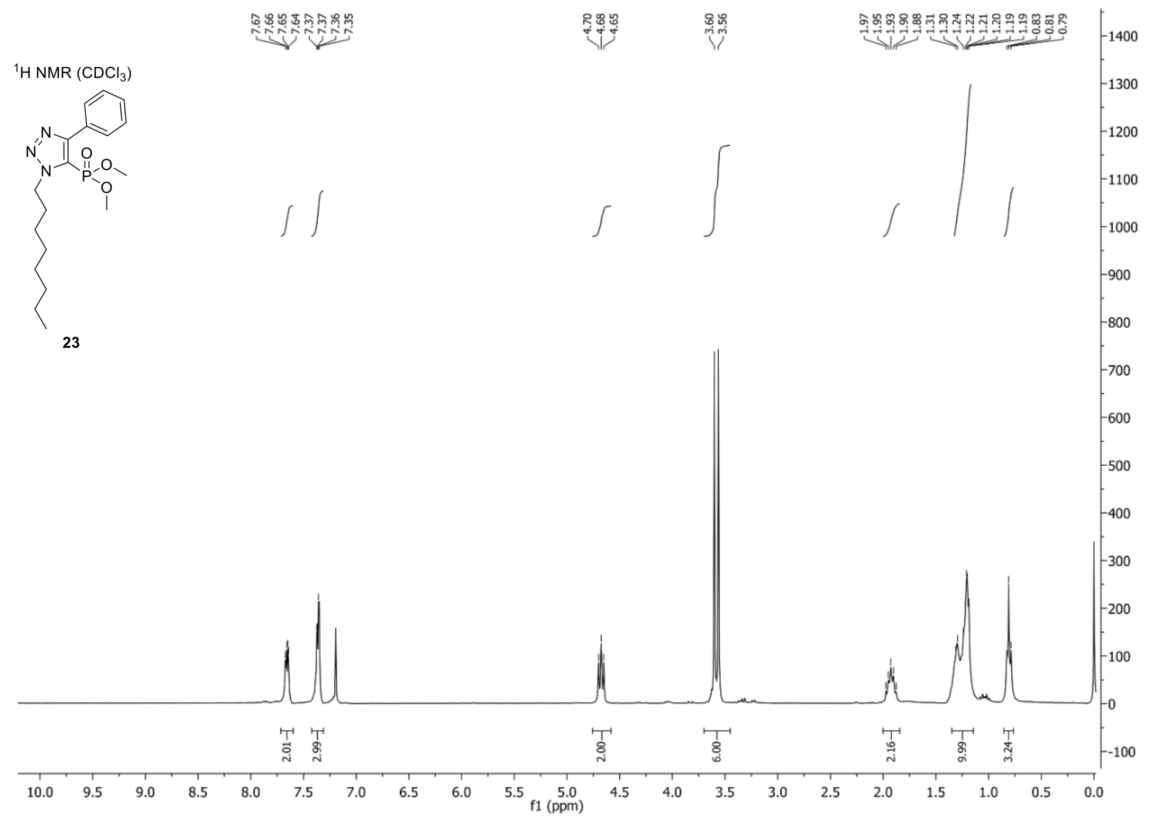
1H NMR (CDCl3)
23
7.67 7.66 7.65 7.64 7.37 7.37 7.36 7.35
4.70 4.68 4.65
3.60 3.56
1.97 1.95 1.93 1.90 1.88 1.31 1.30 1.24 1.22 1.21 1.20 1.19 1.19 0.83 0.81 0.79
2.01 2.99 2.00 6.00 2.16 9.99 3.24
f1 (ppm)

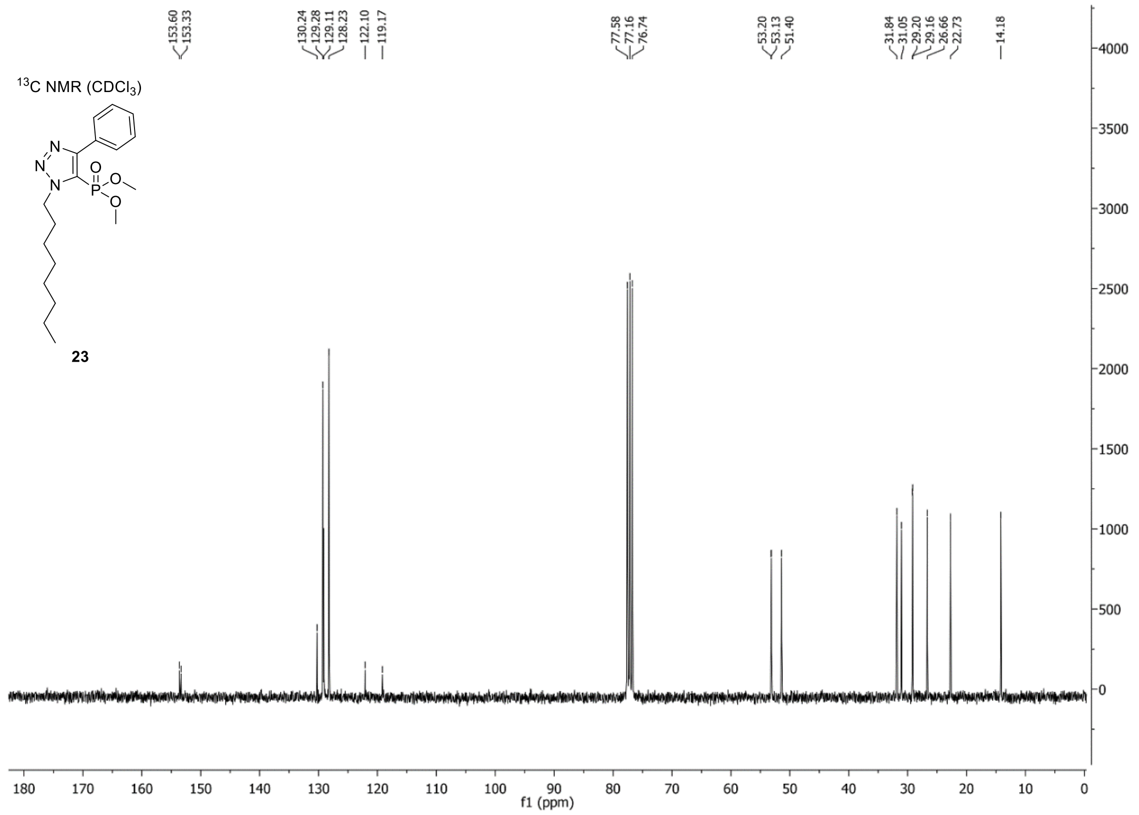
13C NMR (CDCl3)
23
153.60 153.33
130.24 129.28 129.11 128.23 122.10 119.17
77.58 77.16 76.74
53.20 53.13 51.40
31.84 31.05 29.20 29.16 26.66 22.73 14.18
f1 (ppm)

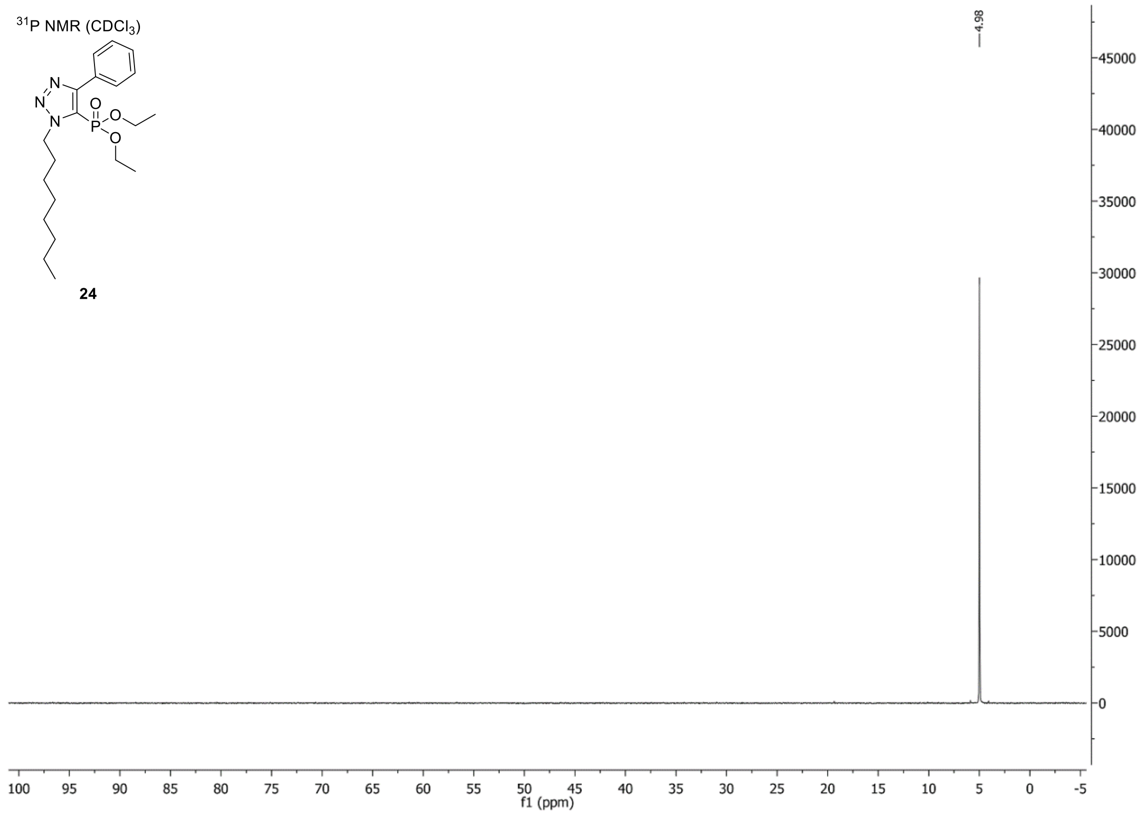
31P NMR (CDCl3)
24
4.98
f1 (ppm)

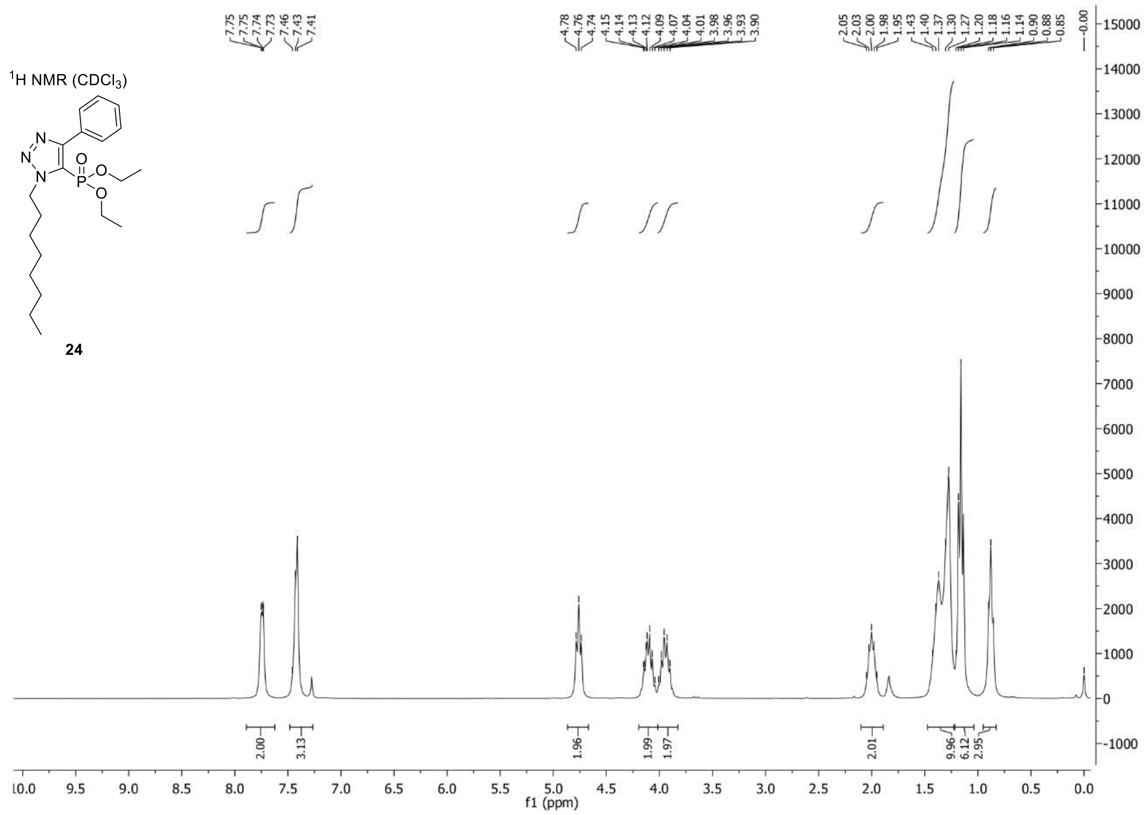
1H NMR (CDCl3)
24
7.75 7.75 7.74 7.73 7.46 7.43 7.41
4.78 4.76 4.74 4.15 4.14 4.13 4.12 4.09 4.07 4.04 4.01 3.98 3.96 3.93 3.90
2.05 2.03 2.00 1.98 1.95 1.43 1.40 1.37 1.30 1.27 1.20 1.18 1.16 1.14 0.90 0.88 0.85
-0.00
2.00 3.13 1.96 1.99 1.97 2.01 9.96 6.12 2.95
f1 (ppm)

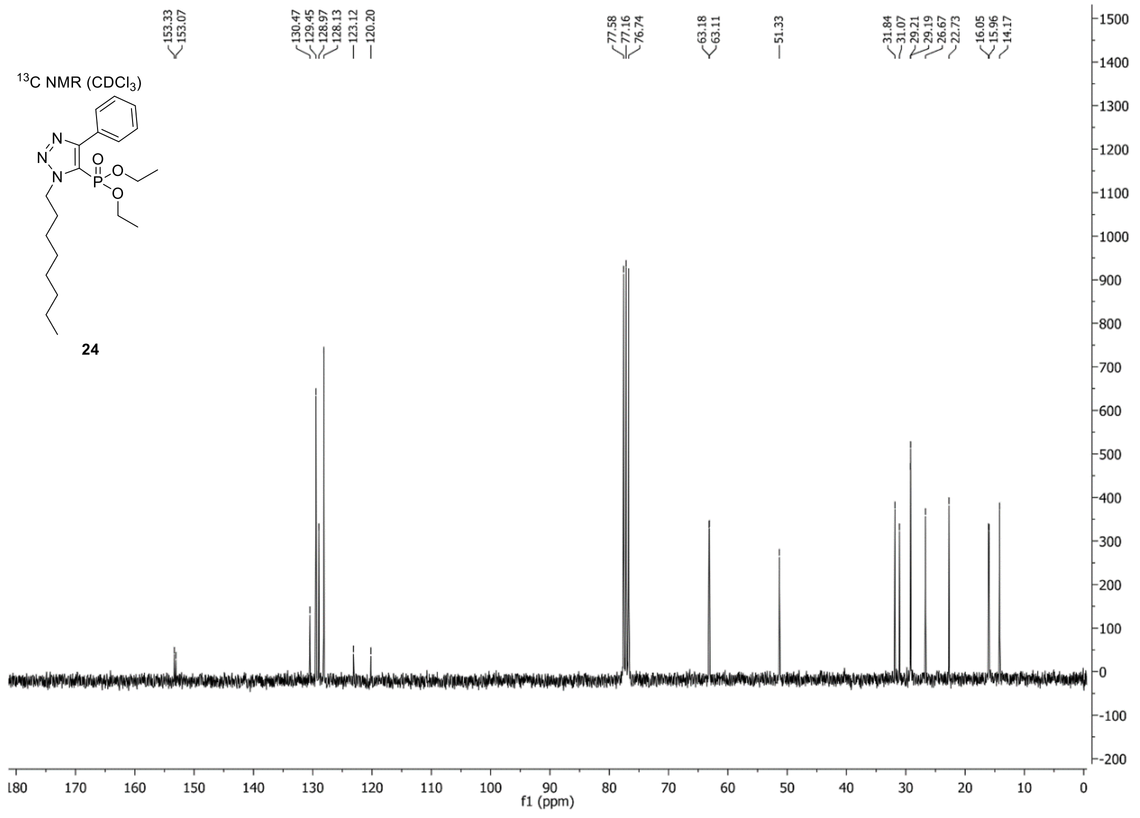

13C NMR (CDCl3)
153.33
153.07
130.47
129.45
128.97
128.13
123.12
120.20
77.58
77.16
76.74
63.18
63.11
51.33
31.84
31.07
29.21
29.19
26.67
22.73
16.05
15.96
14.17
f1 (ppm)

24

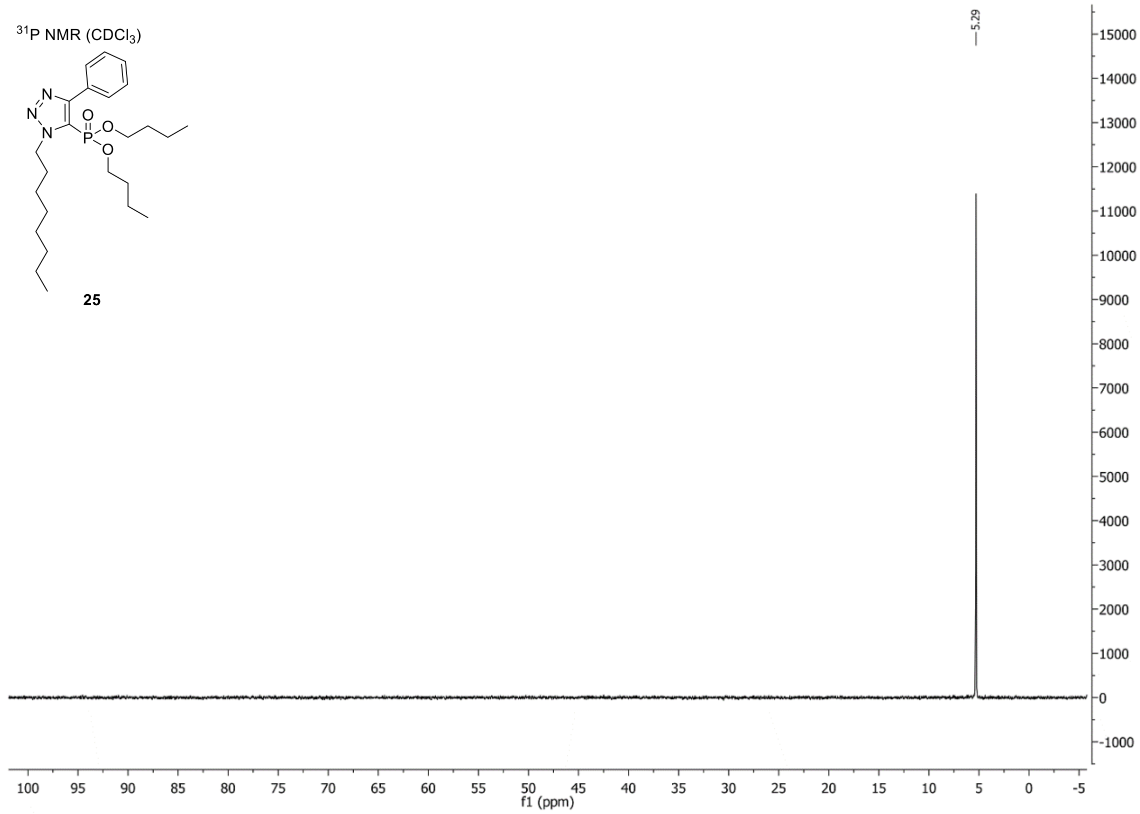

31P NMR (CDCl3)
5.29
f1 (ppm)

25

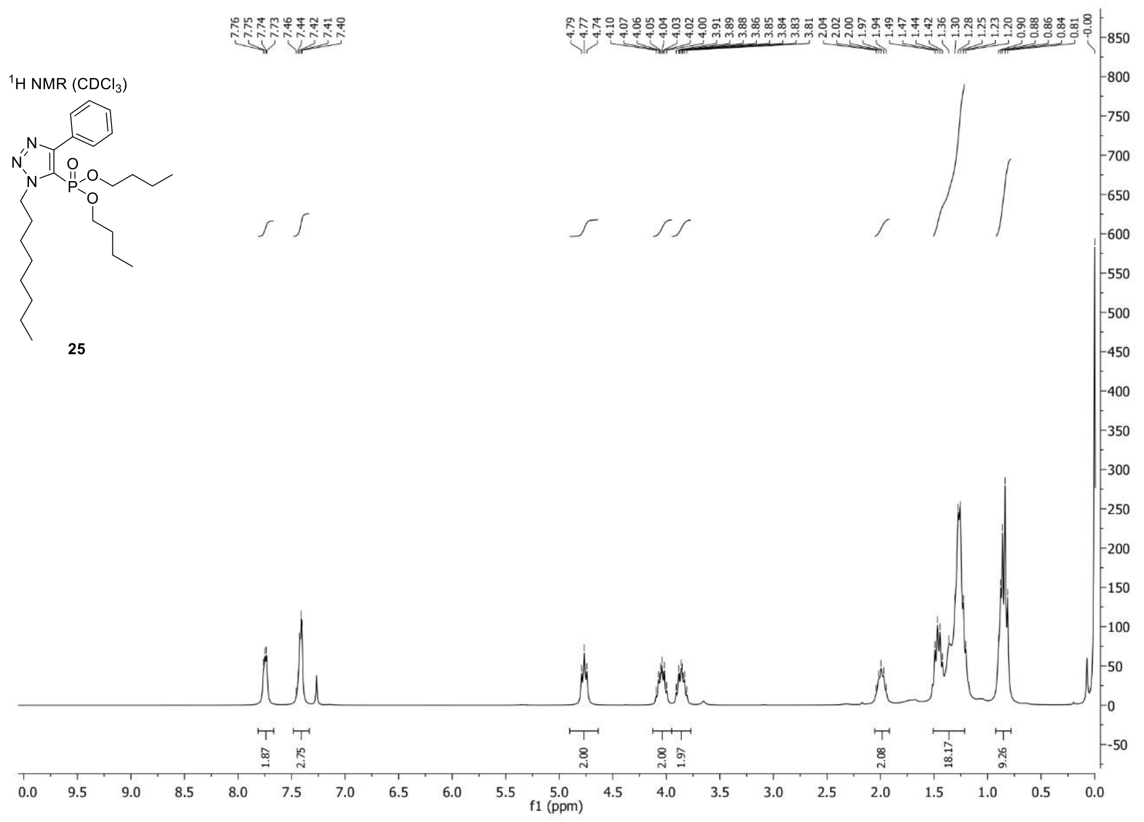
^{1}H NMR ($CDCl_3$)
25
f1 (ppm)

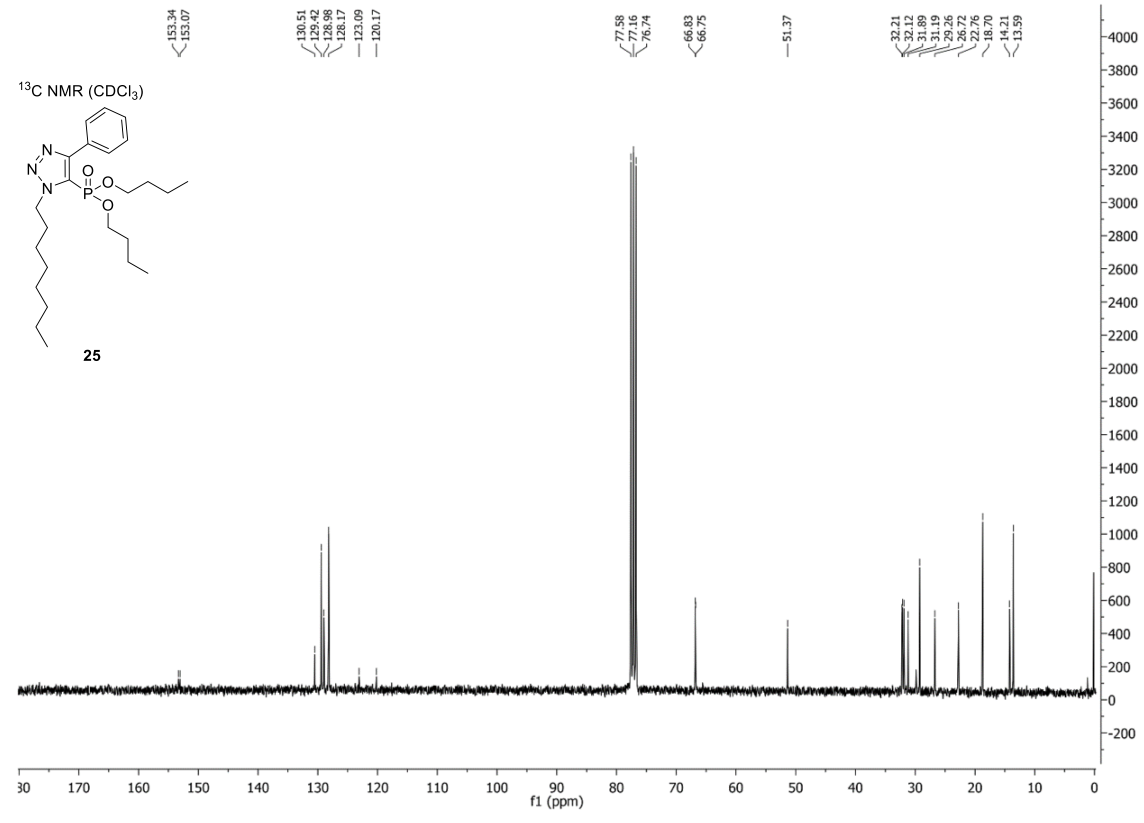
^{13}C NMR ($CDCl_3$)
25
f1 (ppm)

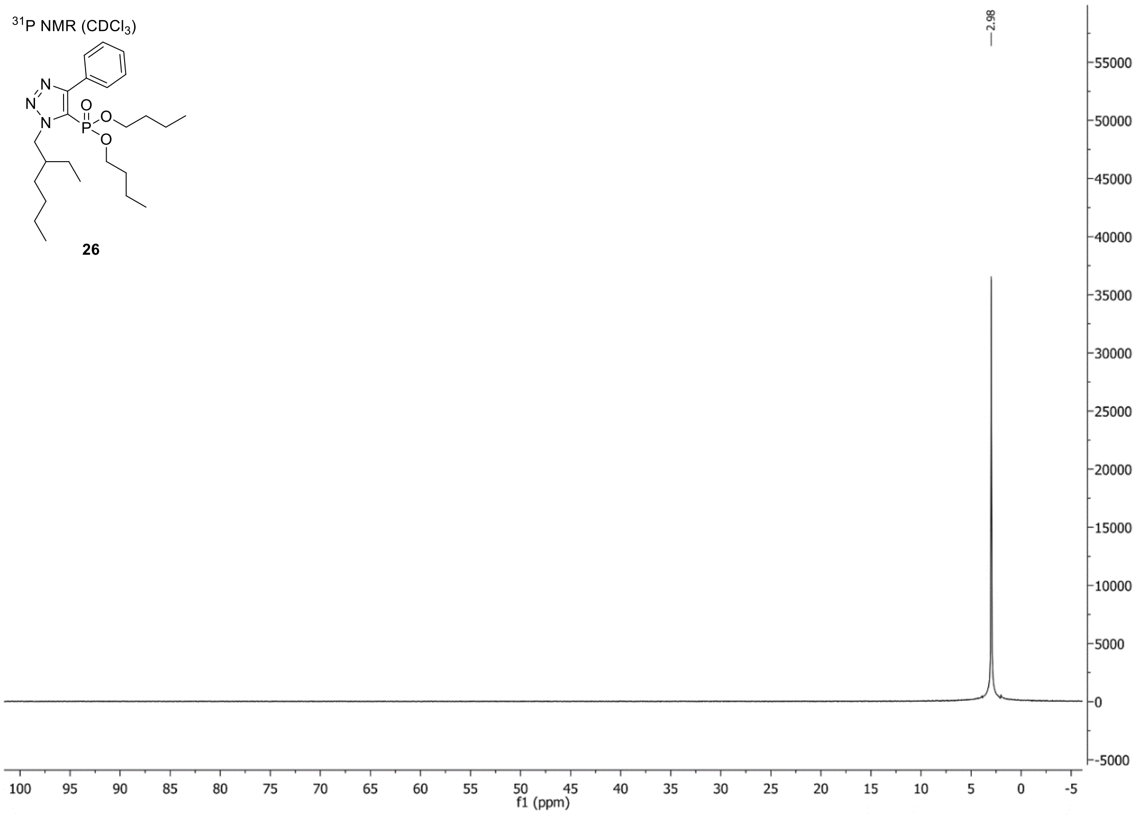
^{31}P NMR (CDCl$_3$)
26
2.98
f1 (ppm)

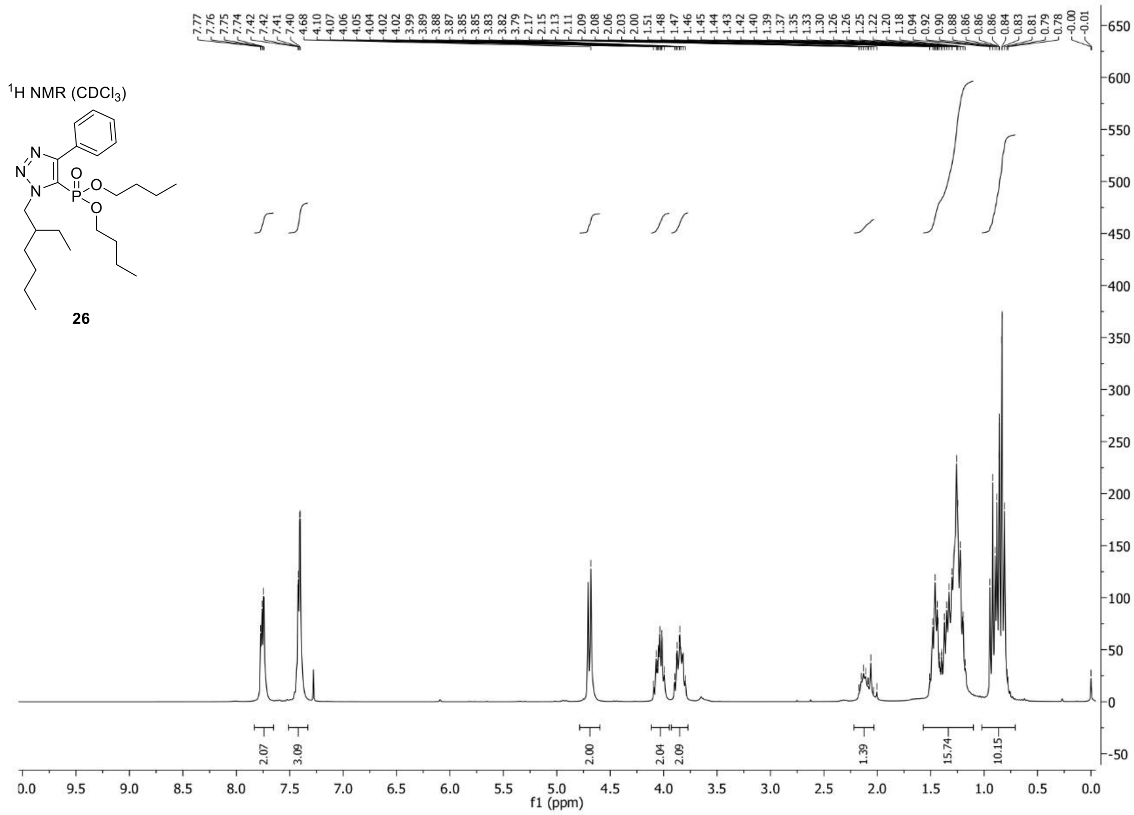
^{1}H NMR (CDCl$_3$)
26
7.77 7.76 7.75 7.74 7.42 7.42 7.41 7.40 4.68 4.10 4.07 4.06 4.05 4.04 4.02 3.99 3.89 3.88 3.87 3.85 3.83 3.82 3.79 2.17 2.15 2.13 2.11 2.09 2.08 2.06 2.03 2.00 1.51 1.48 1.47 1.46 1.45 1.44 1.43 1.42 1.40 1.39 1.37 1.35 1.33 1.30 1.26 1.25 1.22 1.20 1.18 0.94 0.92 0.90 0.88 0.86 0.86 0.84 0.83 0.81 0.79 0.78 -0.00 -0.01
2.07 3.09 2.00 2.04 2.09 1.39 15.74 10.15
f1 (ppm)

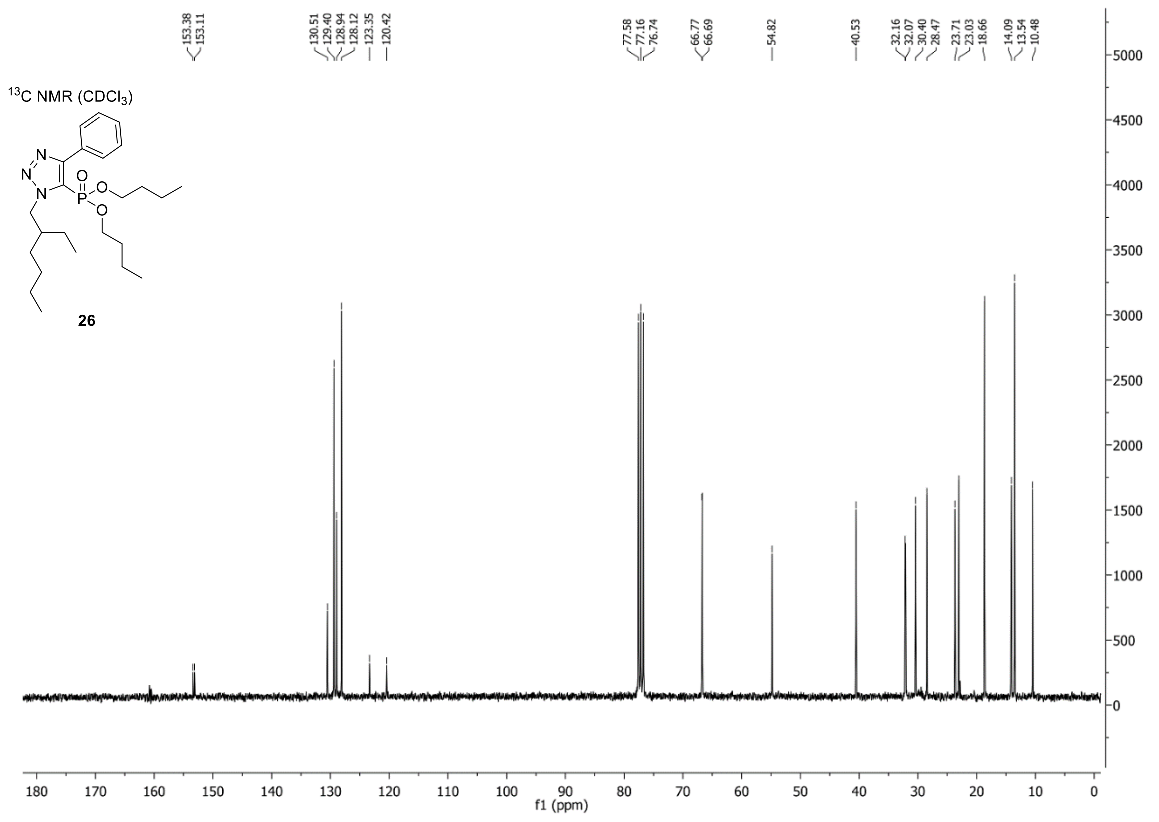
153.38
153.11
130.51
129.40
128.94
128.12
123.35
120.42
77.58
77.16
76.74
66.77
66.69
54.82
40.53
32.16
32.07
30.40
28.47
23.71
23.03
18.66
14.09
13.54
10.48
^{13}C NMR (CDCl$_3$)
26
f1 (ppm)
